# A thermo‐resistant and RNase‐sensitive cargo from *Giardia duodenalis* extracellular vesicles modifies the behaviour of enterobacteria

**DOI:** 10.1002/jex2.109

**Published:** 2023-08-30

**Authors:** Affan Siddiq, George Dong, Balu Balan, Luke G. Harrison, Aaron Jex, Martin Olivier, Thibault Allain, Andre G. Buret

**Affiliations:** ^1^ Department of Biological Sciences University of Calgary Calgary Alberta Canada; ^2^ Inflammation Research Network University of Calgary Calgary Alberta Canada; ^3^ Host‐Parasite Interactions University of Calgary Calgary Alberta Canada; ^4^ Department of Microbiology and Immunology, The Research Institute of the McGill University Health Centre, Program in Infectious Diseases and Immunology in Global Heath Montréal QC Canada; ^5^ The Walter and Eliza Hall Institute of Medical Research Melbourne Australia; ^6^ The University of Melbourne Melbourne Australia

**Keywords:** enterobacteria, exosomes, extracellular vesicles, *G. intestinalis*, *G. lamblia*, *Giardia duodenalis*, giardiasis, microbiota, proteome, small RNA, transcriptome

## Abstract

Extracellular vesicles (EVs) recently emerged as important players in the pathophysiology of parasitic infections. While the protist parasite *Giardia duodenalis* can produce EVs, their role in giardiasis remains obscure. *Giardia* can disrupt gut microbiota biofilms and transform commensal bacteria into invasive pathobionts at sites devoid of colonizing trophozoites via unknown mechanisms. We hypothesized that *Giardia* EVs could modify gut bacterial behaviour via a novel mode of trans‐kingdom communication. Our findings indicate that *Giardia* EVs exert bacteriostatic effects on *Escherichia coli* HB101 and *Enterobacter cloacae* TW1, increasing their swimming motility. *Giardia* EVs also decreased the biofilm‐forming ability of *E. coli* HB101 but not by *E. cloacae* TW1, supporting the hypothesis that these effects are, at least in part, bacteria‐selective. *E. coli* HB101 and *E. cloacae* TW1 exhibited increased adhesion/invasion onto small intestine epithelial cells when exposed to *Giardia* EVs. EVs labelled with PKH67 revealed colocalization with *E. coli* HB101 and *E. cloacae* TW1 bacterial cells. Small RNA sequencing revealed a high abundance of ribosomal RNA (rRNA)‐ and transfer RNA (tRNA)‐derived small RNAs, short‐interfering RNAs (siRNAs) and micro‐RNAs (miRNAs) within *Giardia* EVs. Proteomic analysis of EVs uncovered the presence of RNA chaperones and heat shock proteins that can facilitate the thermal stability of EVs and its sRNA cargo, as well as protein‐modifying enzymes. In vitro, RNase heat‐treatment assays showed that total RNAs in EVs, but not proteins, are responsible for modulating bacterial swimming motility and biofilm formation. *G. duodenalis* small RNAs of EVs, but not proteins, were responsible for the increased bacterial adhesion to intestinal epithelial cells induced upon exposure to *Giardia* EVs. Together, the findings indicate that *Giardia* EVs contain a heat‐stable, RNase‐sensitive cargo that can trigger the development of pathobiont characteristics in Enterobacteria, depicting a novel trans‐kingdom cross‐talk in the gut.

## 1 INTRODUCTION

*Giardia* is a non‐invasive protist parasite of the upper small intestine and a common cause of waterborne diarrheal disease in various species, including humans. The life cycle of *Giardia duodenalis* (synonymous *G. intestinalis*, *G. lamblia*) typically starts with ingesting infectious cysts via the faecal‐oral route or contaminated water or food. After ingestion, cysts undergo excystation, releasing trophozoites, which multiply by binary fission and colonize the upper small intestine (Buchel et al., 1987). The diarrhoea of giardiasis is mostly malabsorptive (Buret et al., 1992). The pathophysiology of the infection is characterized by intestinal barrier dysfunction secondary to increased intestinal epithelial cell (IEC) apoptosis and through the disruption of apical junctional complexes and epithelial villin (Amat et al., 2017; Bartelt & Sartor, 2015; Bhargava et al., 2015; Chin et al., 2002; Cotton et al., 2014; Cotton et al., 2015; Liu et al., 2018; Ortega‐Pierres et al., 2018; Yu et al., 2008). *Giardia* is also known to further disrupt gut barrier function by cleaving intestinal mucins and causing goblet cell hypersecretion and mucus depletion (Amat et al., 2017; Fekete et al., 2022). The infection develops without overt infiltration of inflammatory cells, which results at least in part from the degradation of the host's pro‐inflammatory mediators by the parasite's cysteine proteases (Allain & Buret, 2020; Allain et al., 2019; Liu et al., 2018; Ortega‐Pierres et al., 2018). Another critical aspect of *Giardia*’s pathophysiology is its interaction with the gut microbiota (Beatty et al., 2017; Fekete et al., 2020). While *Giardia* colonization can be attenuated by the host's commensal microbiota (Singer & Nash, 2000), the parasite directly causes microbiota dysbiosis (Fekete et al., 2020; Keselman et al., 2016). Indeed, *Giardia* disaggregates microbiota biofilms and modifies the phenotype of human commensal bacteria by promoting the formation of invasive pathobionts which is caused, at least in part, by trophozoites’ cysteine protease activity (Beatty et al., 2017). Intriguingly, these effects occur in the colon, a site devoid of active trophozoite colonization, *via* unknown mechanisms (Beatty et al., 2017; Halliez & Buret, 2013).

Extracellular vesicles (EVs) are important mediators of cell‐cell communication, and they have been recently implicated in host‐pathogen interactions (Buck et al., 2014; Ferreira et al., 2022; Nievas et al., 2020). EVs are a heterogenous group of 30–1000 nm lipid‐bound vesicles released by cells into the extracellular space (Zaborowski et al., 2015). *Giardia* trophozoites produce exosome‐like vesicles that modulate the endosomal‐sorting complex for transport‐associated AAA+‐ATPase Vps4a and Rab 11, required in the biogenesis processes (Moyano et al., 2019). *Giardia* also appears to secrete both small and large EVs, with different protein contents that play an essential role in adhesion to intestinal epithelial cells (IECs) (Gavinho et al., 2020). Furthermore, *Giardia* EVs can activate naive dendritic cells after internalization (Evans‐Osses et al., 2017). *Giardia* EVs can also be internalized in mouse peritoneal macrophages, activating Toll‐like receptor 2 (TLR2) and NOD‐, LRR‐ and pyrin domain‐containing protein 3 (NLRP3) inflammasome signalling pathways (Zhao et al., 2021).

Furthermore, cells infected with the Respiratory Syncytial Virus release EVs that can promote biofilm formation by *Pseudomonas aeruginosa*, supporting the hypothesis that EVs may represent a mode of trans‐kingdom communication (Hendricks et al., 2021). Our findings reveal a new role for *G. duodenalis* EVs in mediating parasite interactions with the gut *Enterobacteriaceae* species. The studies describe how *G. duodenalis* EVs alter commensal bacteria' growth, phenotype, and behaviour. The overarching hypothesis of the present study was that *G. duodenalis* EVs, *via* their nucleic acid and protein content, can trigger the formation of pathobionts, depicting a novel trans‐kingdom cross‐talk in the gut.

## 2 METHODS

### 2.1 Giardia duodenalis culture

Experiments were performed using *Giardia duodenalis* isolate NF (Assemblage A). The NF isolate was originally obtained from an epidemic outbreak of giardiasis in Newfoundland, Canada (Teoh et al., 2000). Parasites were cultured axenically in TYI‐S‐33 medium supplemented with 10% foetal bovine serum (Gibco) and 0.05% bovine bile (Sigma Aldrich, USA) at 37°C 5% CO_2_ and were used at peak culture density.

### 2.2 Bacterial strains

The present experiments involved two common gut *Enterobacteriaceae* species, *Escherichia coli* strain HB101, a laboratory strain derived from *Escherichia coli* strain K12, and *Enterobacter cloacae* TW1 isolated from a healthy human donor (faeces; IDS014101)*. E. cloacae* TW1 was grown in brain heart infusion (BHI) medium (BD Difco). *E. coli* HB101 was grown in Luria‐Bertani LB broth (BD Difco). Both bacterial cultures were grown at 37°C and 120 rpm in a shaker. The growth of bacteria was measured using optical density at 600 nanometer (nm).

### 2.3 Cell cultures

SCBN cells are non‐transformed duodenal epithelial cells with a canine genotype (Buret & Lin, 2008). SCBN cells were grown in Dulbecco's Modified Eagle's Medium (DMEM) (Sigma Aldrich), supplemented with 10% foetal bovine serum (Gibco), 100 pg/mL streptomycin, 100 U/mL penicillin and 200 mM L‐glutamine (Sigma Aldrich) (Buret & Lin, 2008). Cells were incubated at 37°C and 5% CO_2_ in 96% humidity and grown in flasks and chamber slides.

### 2.4 Isolation of extracellular vesicles

EVs were isolated using the ExoEasy Maxi Kit (Qiagen, Germany) according to the manufacturer's instructions. Briefly, *G. duodenalis* NF was grown to confluence and spent media was discarded without disrupting adherent trophozoites. Culture tubes were washed with warm PBS. Trophozoites were then incubated at 37°C 5% CO_2_ with TYI‐S‐33 media supplemented with 1 mM CaCl_2_, a known activator of *Giardia* EV formation (Evans‐Osses et al., 2017), 5 mg/mL bovine bile (Sigma Aldrich), or left untreated. Exosome‐free FBS (ThermoFisher) was used in the TYI‐S‐33 media formulation. After 60 min, the spent media were transferred to 15 mL tubes, centrifuged at 1500 × *g* for 10 min at 4°C and filtered using a 0.8 μm filter (Corning). A 1:1 volume of the binding buffer (Qiagen) was added to each sample, and the mixture was inoculated in a purification column and centrifuged at 500 × *g* for 1 min. Samples were washed with XWP buffer, and purification columns were transferred to a new collection tube. Samples were eluted with elution buffer (Qiagen) in LoBind tubes (Eppendorf). The tubes were stored in XE buffer at −80°C until further use.

### 2.5 Nanosight analysis of *Giardia* EVs

EVs were diluted 1:10 in sterile phosphate buffer saline (PBS) (Sigma Aldrich) and analysed using a NanoSight 300 (Malvern Paranalytical) and NTA software version 2.3. Each sample was run in five technical replicates. The following parameters were selected when analysing the EV samples: the screen gain was set at seven, the camera level was adjusted to five, the detection threshold was set at four, and the filter wheel was set at 535 nm. Images were captured for 60 s.

### 2.6 Negative staining for transmission electron microscopy analysis

Untreated, heat‐treated, and RNase‐treated *G. duodenalis* isolate NF EVs were analysed by transmission electron microscopy (TEM) negative staining. EV samples were placed on formvar Carbon grids. Grids were placed onto droplets of 1% glutaraldehyde for 1 min and washed three times for 1 min with drops of double‐distilled H_2_O. Next, grids were stained with 1% uranyl acetate for 1 min and dried for 30 min. This process was repeated one time. Dry grids were visualised using an FEI Tecnai G2 Spirit Twin transmission electron microscope (120 kV Cryo‐TEM) and Gatan Ultrascan 4000 4k × 4k CCD Camera System (Model 895) at the McGill University Facility for Electron Microscopy Research.

### 2.7 Protein quantification of EVs

The protein content of EVs was quantified using a Micro BCA Protein assay kit (ThermoFisher) according to the manufacturer's protocol. Briefly, 150 μL of each provided standard solution and EVs suspended in elution buffer were added into a microplate well. Then, 150 μL of the working reagent (WR) was added to each well and mixed thoroughly using a plate shaker for 30 s. The plate was then sealed and incubated at 37°C for 2 h. After incubation, the plate was cooled to room temperature. The absorbance reading was taken at 562 nm on a SpectraMax M2e microplate reader (Molecular Devices, Sunnyvale, CA). After adjusting the readings by subtracting the blank from the standard replicates from the samples, a standard curve was generated. This standard curve was used to determine *Giardia* EV samples' protein concentration.

### 2.8 Proteomic analysis of extracellular vesicles

#### 2.8.1 Protein extraction and precipitation from EVs

EV protein extraction and precipitation for proteomics analysis were performed based on a previously published method (Atayde et al., 2019). In brief, before proteomic analysis, proteins derived from purified *G. duodenalis* EVs were extracted using trichloroacetic acid (TCA)/sodium deoxycholate precipitation method (10X TE buffer, 0.3% sodium deoxycholate, and 72% TCA). The samples were then incubated on ice for one hour. Next, samples were centrifuged at 14,000 × *g* for 20 min at 4°C. Pellets were resuspended in 90% methanol and incubated overnight at −20°C. Samples were centrifuged at 14,000 × *g* for 20 min at 4°C, and the protein pellets were air‐dried for 15 min and stored at −80°C.

#### 2.8.2 Protein digestion and liquid chromatography–MS/MS (LC–MS/MS)

Liquid Chromatography with tandem mass spectrometry (LC–MS/MS) was performed at the proteomic platform of the Institut de Recherches Cliniques de Montréal (Montréal, Canada) based on previously described methods (Atayde et al., 2019). In brief, in‐solution digestion was performed after precipitation by adding trypsin at a ratio of 1:25 protease/protein ratio. After overnight incubation at 37°C, the reactions were quenched by adding formic acid to a final concentration of 0.2% and cleaned with C18 ZipTips (Millipore) before MS analysis. Extracted peptides were injected onto a Zorbax Extended‐C18 desalting column (Agilent) and subsequently chromatographically separated on a Biobasic 18 Integrafrit capillary column (Thermo Scientific) on a Nano high‐performance liquid chromatography system (1100 series unit; Agilent). Eluted peptides were electro sprayed as they exited the capillary column and were analysed on a QTRAP 4000 linear ion trap mass spectrometer (SCIEX/ABI).

#### 2.8.3 Protein identification

Samples were analysed using Mascot (Matrix Science, London, UK; version 2.3.02). Mascot was set up to search the NCBI *Giardia duodenalis* database (17505 entries). The mass tolerances for precursor and fragment ions were set to 10 ppm and 0.6 Da, respectively. Trypsin was used as the enzyme, allowing for up to one missed cleavage. Cysteine carbamidomethylation was specified as a fixed modification and methionine oxidation as variable modification. Data analysis was performed using Scaffold (version 4.11.0). Peptide identifications were accepted if they could be established with a probability greater than 80%. Protein identifications were accepted if they could be found at greater than 95% probability and contained at least two identified peptides. Protein probabilities were assigned by the Protein Prophet algorithm (Nesvizhskii et al., 2003). Proteins that contained similar peptides and could not be differentiated using MS/MS analysis alone were grouped to satisfy the principles of parsimony. Gene ontology and KEGG pathway enrichment analysis were performed using DAVID. Protein‐protein interaction networks were mapped using the string network analysis.

### 2.9 Small RNA sequencing of extracellular vesicles

#### 2.9.1 Total RNA purification, small RNA deep‐sequencing and data analysis

Isolated EVs from *Giardia* were sent for sequencing and bioinformatics analysis to LC Sciences (Houston, TX). The subsequent steps were performed there. First, total RNA from control and bile‐induced EVs was extracted using Trizol reagent (Invitrogen) following the manufacturer's procedure. Next, approximately 1 μg of total RNA was used to prepare a small RNA library according to the protocol of TruSeq Small RNA Sample Prep Kits (Illumina, San Diego, USA). And then, the single‐end sequencing 50 bp was performed on an Illumina Hiseq 2500 at the LC Sciences (Hangzhou, China) following the vendor's recommended protocol.

Read quality of the raw reads from control and bile‐treated were estimated using FastQC (Andrews, 2010). Adapters were trimmed, and low‐quality reads and contaminations were removed using Trimmomatic (Bolger et al., 2014). FastQC (Andrews, 2010) was re‐run post‐trimming on the reads to ensure the adapters were trimmed and low‐quality bases removed. Trimmed reads were then mapped to *G. duodenalis* WB genome (GiardiaDB‐54_GintestinalisAssemblageAWB_Genome.fasta) using subread (Law et al., 2016). Next, the feature count's function (Law et al., 2016) was employed on the bam files obtained from the subread. The counts data were then analysed using the edgeR pipeline (http://bioconductor.org/packages/devel/bioc/vignettes/edgeR/inst/doc/edgeRUsersGuide.pdf) to perform differential expression (Law et al., 2016). EdgeR function ‘filterByExpr’ was used to remove low abundance transcripts, and the reads were normalised using Trimmed Mean of M‐values (TMM) normalization. One biological replicate was analysed per condition. We used a common BCV (square root‐dispersion) of 0.1 in the extract test function to account for biological variability. Small RNAs were considered significantly differentially transcribed at a false discovery rate (FDR) > 0.05 and a minimum log_2_‐fold change (FC) > 1; log_2_‐FC ← 1.

### 2.10 Micro‐RNA (miRNA) and small non‐coding RNA analysis

Further analysis on mapping novel miRNAs and quantifying known miRNAs was performed using the miRDeep2 package (Friedlander et al., 2012). To identify *Giardia* miRNA seeds similar to that of miRNAs from model organisms (asu, bma, cbn, cbr, cel, crm, hco, ppc, prd, str, bpa, sra, hpo, cte, gpy, tre, hru, lgi, cla, egr, emu, sja, sma, sme, gsa, fhe, mco, mle, hma), we extracted the precursor and mature miRNAs from the miRBase database (Release 22) (http://www.miRbase.org/) (Law et al., 2016) and used them as a reference in the miRDeep2 analysis.

### 2.11 Micro‐RNA (miRNA) target gene prediction

Following the identification and quantification of the miRNAs, to understand what untranslated regions (UTRs) these miRNAs regulate, we extracted 3′UTRs of *Giardia* transcripts using GET UTR version 2.0 (Kim et al., 2015). 5′ UTRs are excluded as they are small in *Giardia* (1–14 nucleotide), and miRNAs are more preferential to 3′ UTRs than 5′ UTRs. Miranda 3.3a program was used (http://www.microrna.org/) (Enright et al., 2003) to identify miRNA‐UTR. Based on the miRanda scoring system, the top‐scored (pairing Score ≥ 150) miRNA‐UTR interactions are filtered out. The gene list corresponding to the 3′ UTRs we obtained was used to perform gene ontology (GO) and PFAM domain enrichment using DAVID (Huang et al., 2007a, 2007b). Finally, the protein‐protein network map was plotted using the STRING network mapping tool (Szklarczyk et al., 2019).

### 2.12 *Giardia* EVs small RNA target prediction in *E. coli* HB101

In an attempt to better understand the molecular mechanisms whereby *Giardia* EVs communicated with bacteria, in silico analysis of *E. coli* mRNAs targeted by *Giardia* EV‐ derived sRNAs were predicted by TargetRNA (Kery et al., 2014). In brief, the *Giardia* EV‐derived sRNAs were compared to all the bacterial replicons in Ref Seq database using BASTN. Sequences within the bacterial replicons significantly conserved compared to *Giardia* EV sRNAs were extracted and aligned using ClustalW2. The aligned file was then used to compute positional entropies that identified sRNA sequence regions that were highly conserved. Vienna RNA Package was used to assess the accessibility of the target‐interacting regions of the sRNAs, and RNAplfold package was employed to determine the accessibility of bacterial mRNA sequences. Finally, RNAduplex was used to assess the hybridization energy levels between *Giardia* EVs sRNAs and HB101 mRNA targets. *E. coli mRNA*‐*Giardia* sRNAs interactome was illustrated as significantly enriched protein‐protein interaction network clusters (*p* < 1*10^−16^).

### 2.13 Extracellular vesicle immunofluorescence assays

PKH67 dye (Sigma Aldrich, USA) was used to label EVs. First, EVs were diluted in the elution buffer from the ExoEasy maxi kit (Qiagen) in a final volume of 500 μL to get a final concentration of 108 EVs/mL. Next, the PKH67 dye (1.6 μL) was diluted in Diluent C (500 μL) to make a PKH 67 solution. Then, the PKH67 solution was mixed with the diluted EVs solution in a 1:1 ratio. The samples were then incubated at 37°C for 5 min to allow the labelling reaction to occur. The labelling reaction was stopped using 800 μL of exosome‐depleted FBS (ThermoFisher). The labelled EVs from this solution were then isolated using the ExoEasy maxi kit (Qiagen). Briefly, the solution was added to the spin columns, 10 mL of wash buffer (XWP) (Qiagen) was added to the spin columns, and the tubes were spun at 5000 × *g* for 5 min. The spin columns were then transferred to a new collection tube, and 400 μL of XE elution buffer (Qiagen) was added to the column, followed by 5 min of incubation. Next, the samples were centrifuged at 500 × *g* for 5 min, and the eluates were transferred to the Eppendorf tubes LoBind (Eppendorf). The labelled EVs were then incubated with the bacterial cultures when the optical density reached 0.4 (O.D_600nm_ = 0.4) for 1 h at 37°C in a microcentrifuge tube. After incubation, bacterial cultures were centrifuged at 5000 × *g* for 5 min and washed with warm sterile PBS. Cultures were then spotted on a microscopic slide and stained with DAPI with mounting media. Coverslips were placed on the slides, and micrographs were obtained using a Leica DMR Microscope with a Retiga 2000R camera (Q Imaging, BC).

### 2.14 Bacterial kinetics experiments

Experiments assessed the effects of *Giardia* EVs on bacterial growth. *Escherichia coli* HB101 and *Enterobacter cloacae* TW1 were cultured and grown overnight in LB and BHI broth in a shaker at 37°C and 120 rpm. The optical densities of bacterial cultures were adjusted to 0.01 before the experiment so that the lag, exponential, and stationary phases could be observed (*E. coli* HB101 O.D_600nm_ = 0.01 ⇔ 3.52 × 10^7^ CFU/mL; *E. cloacae* TW1 O.D_600nm_ = 0.01 ⇔ 5.66 × 10^7^ CFU/mL). Bacteria were then incubated with different *Giardia* EV concentrations treated with 5 mg/mL bile to maximize EV yield. Bacteria were exposed to EVs for 12 h, and the bacterial density (OD_600 nm_) was measured at one‐hour intervals to monitor the effects of *Giardia* EVs on the growth of the bacteria.

### 2.15 Bacterial swimming motility assays

Using the above protocols, *E. coli* HB101 and *E. cloacae* TW1 were incubated with *G. duodenalis* EVs for 24 h, and effects on swimming motility were assessed. After 24 h, bacterial cultures were normalised based on O.D_600nm_ = 0.1, and swimming motility was determined in low‐viscosity conditions on 0.3% agar plates. To assess motility, sizes of growth halos were measured at 24, 48 and 72 h. In addition, halo surface area (cm^2^) was measured using ImageJ software (National Institutes of Health).

### 2.16 In vitro bacterial adhesion/invasion assays

Non‐transformed duodenal epithelial SCBN cells were used to determine how exposure to *Giardia* EVs may affect bacterial adhesion/invasion to host epithelial cells. SCBN cells were seeded at 2.5 × 10^4^ cells/well in 24 well plates (Corning, USA) and grown to the confluence at 37°C and 5% CO_2_. The cells were exposed to *E. coli* HB101 or *E. cloacae* TW1 that were incubated with different EV concentrations for 24 h or left untreated at an MOI of 100 CFUs/cell for 3 h. After 3 h, the cells were washed with PBS and then permeabilised using 1% saponin (Sigma Aldrich, St. Louis, USA) for 5 min to assess cell adhesion/invasion. Bacteria were enumerated by spreading serial dilutions onto Luria broth (LB) agar for *E. coli* HB101 and Brain Hear Infusion (BHI) agar for *E. cloacae* TW1, followed by aerobic incubation overnight at 37°C.

### 2.17 Measurement of bacterial biofilm total biomass

Maintenance of microbiota biofilm integrity is critical to gut homeostasis. Previous research demonstrated that *Giardia* could disrupt these biofilms via unclear mechanisms (Beatty et al, 2017). The ability of bacteria to form biofilms was tested using a Calgary biofilm device (CBD) assay with a 96‐well base (Innovotech, Canada) (Ceri et al., 1999). *E. coli* HB101 and *E. cloacae* TW1 were incubated with *Giardia* EVs for 24 h. The optical densities of bacterial cultures were adjusted to 0.01 (*E. coli* HB101 O.D_600nm_ = 0.01 ⇔ 3.52 × 10^7^ CFU/mL; *E. cloacae* TW1 O.D_600nm_ = 0.01 ⇔ 5.66 × 10^7^ CFU/mL and bacteria were inoculated in a 96 well plate. The CBD peg lid was then placed on the microtiter base. Plates with CBD lids were incubated in the shaker (120 rpm) in humid conditions for 48 h at 37°C and 5% CO_2_. After 48 h, the CBD pegs were washed with warm PBS, dried, and stained with crystal violet for 10 min to assess biofilm mass. The pegs were then rinsed in water, dried, and placed in a 96‐well plate containing 30% acetic acid for 10 min. Finally, after the incubation, optical density was measured at 550 nm using a SpectraMax M2e microplate reader (Molecular Devices).

### 2.18 Heat and RNase A treatment of *G. duodenalis* EVs

Additional experiments further characterized the *Giardia* EVs cargo by using heat and RNAse treatments and assessing the EVs biological effects. EVs were incubated at 95°C for 15 min to denature the proteins and immediately used for further biological assays. EVs were also treated with RNase A to digest the RNA content according to the previously published method (Enderle et al., 2015). As described above, EVs were bound to a membrane affinity column during isolation using the ExoEasy maxi kit. Subsequently, 500 μL of a digestion mix containing 10 μg/mL RNase A and 1% saponin in wash buffer (XWP) was added directly to the membrane, followed by a 1‐min spin at 100 × *g* and re‐application of the flow‐through. After 30 min of incubation at room temperature, the digestion mix was removed by washing the column with 10 mL of XWP. The EVs were then eluted using the elution buffer. As described earlier, heat‐treated and RNase A‐treated EVs were used to characterise effects on bacterial swimming, adhesion/invasion, and biofilm assays.

### 2.19 Statistical analysis

The normality of the data was assessed before statistical analysis. Statistical significance was determined by one‐way ANOVA with the Kruskal‐Wallis post‐test for non‐parametric data. Comparisons between groups with normal distributions were performed using one‐way ANOVA followed by Tukey's test for multiple comparison analyses. Mann Whitney's *U* test was used to compare two non‐parametric data sets. *p* < 0.05 was considered statistically significant. Data were expressed as mean ± S.E.M. Data representation and statistical analysis were performed using GraphPad Prism 8 software for Macintosh (San Diego, USA).

## 3 RESULTS

### 3.1 Characterization of *G. duodenalis* EVs

**FIGURE 1:**
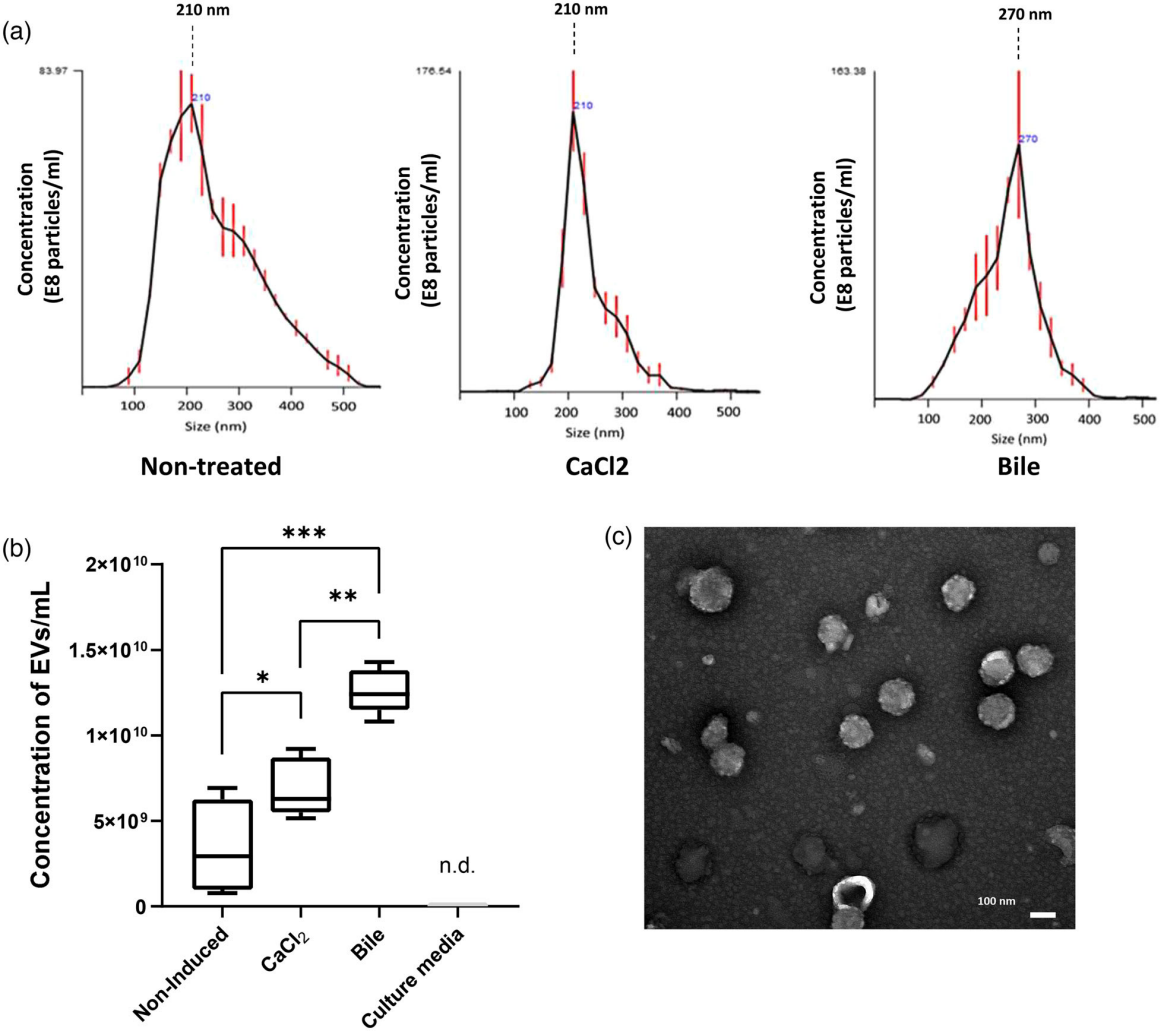
Morphological characterization of *G. duodenalis* EVs. (a) Particle size estimation (nm) and concentration (particle/ml) by Nanoparticle Tracking Analysis (NanoSight NS300). *G. duodenalis* isolate NF trophozoites were treated for one hour with CaCl_2_ (1 mM), bovine bile (5 mg/mL), or left untreated (non‐treated). (b) Concentration of *G. duodenalis* EVs (EVs/mL) measured by Nanoparticle Tracking Analysis, following treatment with CaCl_2_ (1 mM), bovine bile (5 mg/mL) or untreated. EVs were not detected (n.d.) in culture media alone. (c) TEM micrograph of *G. duodenalis* isolate NF EVs. n = 4–5 per group. Results are expressed as mean ± SEM. * *p* < 0.05, ** *p* < 0.01, *** *p* < 0 0.005.

To examine whether exposure to different stimuli may affect the production of EVs by *Giardia*, we exposed the trophozoites to calcium chloride (CaCl_2_), a common inducer of microvesicle formation (Evans‐Osses et al., 2017), and bile, to which the trophozoites are exposed during their colonisation of the upper small intestine. NTA analysis showed that the average size of EVs under CaCl_2_ exposing condition was similar to untreated controls. In contrast, bile‐treated (5 mg/mL) trophozoites produced larger EVs in size (270 nm) (Figure 1a). Quantification of EVs production using NTA revealed that the numbers of EVs produced by *Giardia* trophozoites when exposed to bile were significantly higher than EVs made when *Giardia* trophozoites were exposed to CaCl_2_ (1 mM) or when the trophozoites were left untreated (Figure 1b). Importantly, when observed under the microscope, trophozoites did not undergo encystation after 1‐h exposure to bile (5 mg/mL) or CaCl_2_ and were morphologically similar to the trophozoites from the untreated group. Further characterisation of EVs was done using transmission electron microscopy. The TEM micrographs revealed a characteristic spherical morphology with an identifiable lipid bilayer (Figure 1c). No significant size differences were observed on TEM micrographs between the groups.

### 3.2 RNA‐sequencing analyses of *G. duodenalis* EVs small RNAs

**FIGURE 2:**
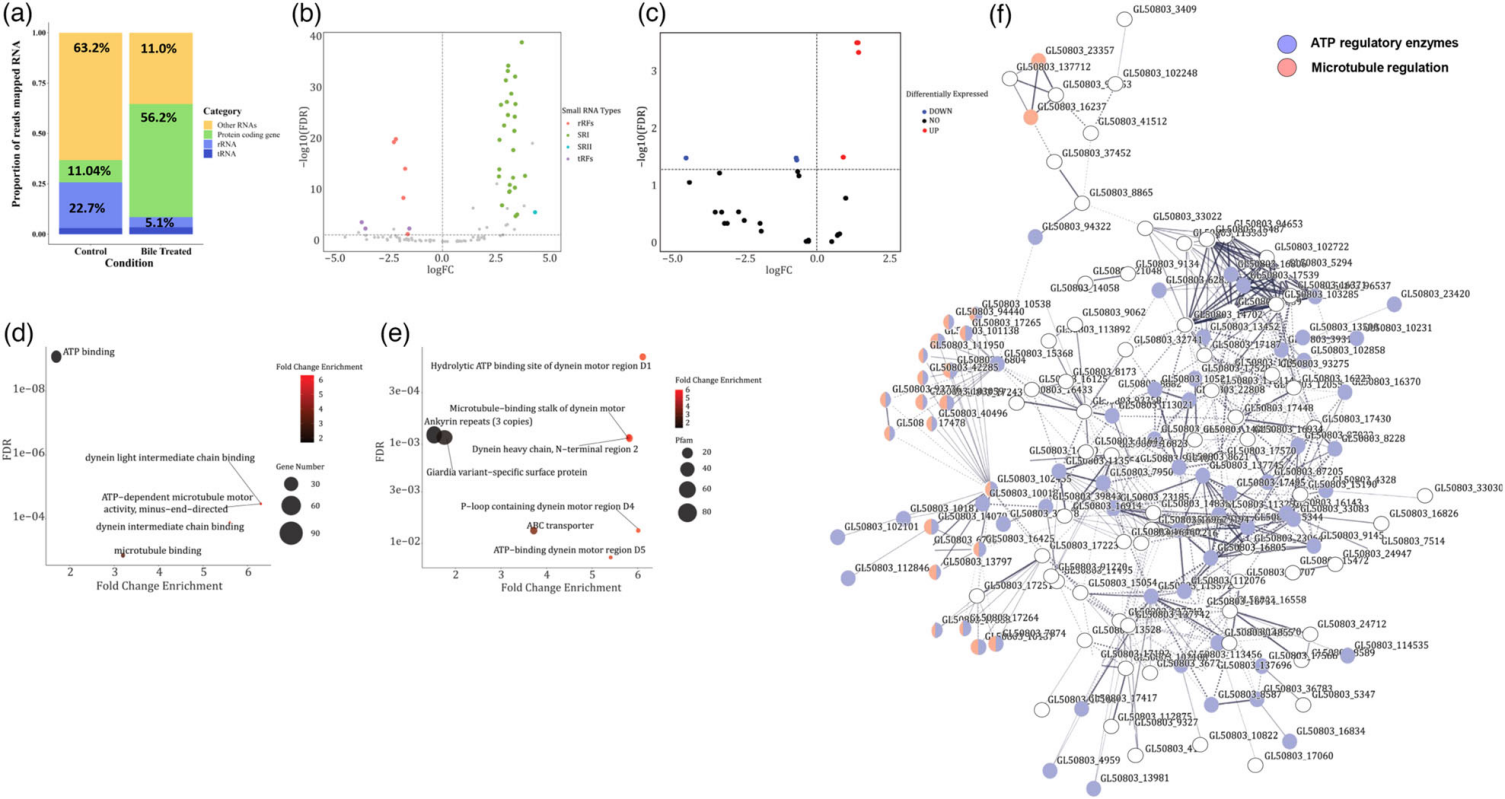
Small RNAs analysis of *G. duodenalis* EVs. *G. duodenalis* NF EVs small RNAs and miRNAs content was analyzed using deep small RNA sequencing (sRNA‐Seq). (a) relative proportion of the reads that corresponds to each of the RNA species (rRNA, mRNA/proteins coding gene, tRNA and other RNAs mapped within the total RNA reads that are mapped against the *G. duodenalis* WB genome in control and bile‐induced EVs.; (b) Volcano plot showing the significantly differentially expressed small RNAs (Log of fold change (LogFC > = 1; LogFC < = −1). FDR‐adjusted *p*‐value (‐log10(FDR > 1.3))) in the contrast control relative to bile‐induced EVs, with small rRNA‐derived fragments (rRFs), endo‐siRNAs (SRI, SRII) and tRNA‐Derived Fragments (tRFs) shown in colour. (c) Volcano plot showing the significantly differentially expressed miRNAs (Log of fold change (LogFC > = 1; LogFC < = −1). FDR‐adjusted *p*‐value (‐log10(FDR > 1.3)) that were identified in the contrast control relative to bile‐treated trophozoites EVs. (d, e) *In silico* predicted *Giardia* mRNA 3′‐UTRs (score > = 150) targets of *G. duodenalis* EVs recruited miRNAs corresponding to mRNAs that are significantly (FDR < 0.05) enriched for (d) Gene Ontology terms and (e) Pfam terms related to microtubule regulation network complexes and ATP binding proteins and ATP regulatory enzymes; (f) Protein‐protein network interaction mapping followed by functional enrichment analysis also showed majorly microtubule regulation network complexes indicated in red dots and ATP binding proteins and ATP regulatory enzymes shown in blue dots as the statistically significantly enriched (*p* < 0.05) functional networks.

Our analysis revealed that within the control EVs, the mapped sRNAs are mRNA‐derived sRNAs (11.04%), rRNA‐derived sRNA (22.69%), tRNA‐derived sRNA (3.05%), and sRNAs derived from other RNAs (63.20%) (Figure 2a). In bile‐treated EVs, the various mapped sRNAs are mRNA‐derived sRNA (56.21%), rRNA‐derived small RNA (5.09%), tRNA‐derived small RNA (3.39%), or sRNAs derived from the other RNAs (35.29%) (Figure 2a). From the small RNA (sRNA) sequencing analyses, we identified 50 sRNAs that were significantly differentially expressed (LogFC ≥ 1; LogFC ≤ 1; FDR < 0.05) when comparing control versus bile‐treated EVs (Figure 2a,b; Table S1). Of the 39 up‐regulated small RNAs (LogFC ≥ 1; FDR < 0.05) (Figure 2a,b; Table S1), we observed a specific class of small RNAs named endo‐siRNAs predominantly up‐regulated in response to bile treatment (Liao et al., 2014). Among the three subtypes of endo‐siRNAs (SRI, SRII, SRIII) (Liao et al., 2014), the most abundant type identified in *Giardia* EVs is the SRI (Figure 2b; Table S1). Among the 11 downregulated small RNAs (LogFC≤1; FDR < 0.05), we observed four tRNA‐derived small RNA and five rRNA‐derived small RNAs (Figure 2b; Table S1). While endo‐siRNAs were previously reported to be highly expressed in *Giardia* trophozoites during encystation (Liao et al., 2014), this is the first time that endo‐siRNAs are identified in *Giardia* EVs in response to bile treatment. Mapping of miRNAs in *Giardia* EVs revealed 108 miRNAs in the control (six novel miRNAs and 102 known miRNAs) and 70 miRNAs in the bile‐treated group (four novel miRNAs and 66 known miRNAs) (Table S2; Table S3). Comparison between EVs derived from bile‐treated trophozoites and control EVs revealed that 13 miRNAs were up‐regulated (LogFC > 0, adjusted *p* < 0.05) and three miRNAs were down‐regulated (Log2FC < 0, adjusted *p* < 0.05) (Figure 2c; Table S4). miRanda analysis was then performed using the miRNAs significantly up‐regulated (LogFC > = 0, adjusted *p* < 0.05) against the 3′UTRs of *Giardia* annotated transcripts. A total of 2843 potential binding interactions were identified between *Giardia* miRNAs and predicted 3′‐UTRs (score ≥ 150), identifying 624 *Giardia* genes (Table S5). Gene Ontology and PFAM analysis enrichment analysis (FDR < 0.05) showed microtubule complex and microtubule‐dependent movement and ATP binding proteins as the regulatory niche of these miRNAs (Figure 2d,e; Table S6). This was re‐confirmed by the protein interaction network analysis using the string pathway enrichment analysis (Figure 2f). Together the analyses revealed that *Giardia* miRNAs contained in EVs can regulate the microtubule network complex.

### 3.3 Proteomic analysis of *G. duodenalis* EVs

**FIGURE 3:**
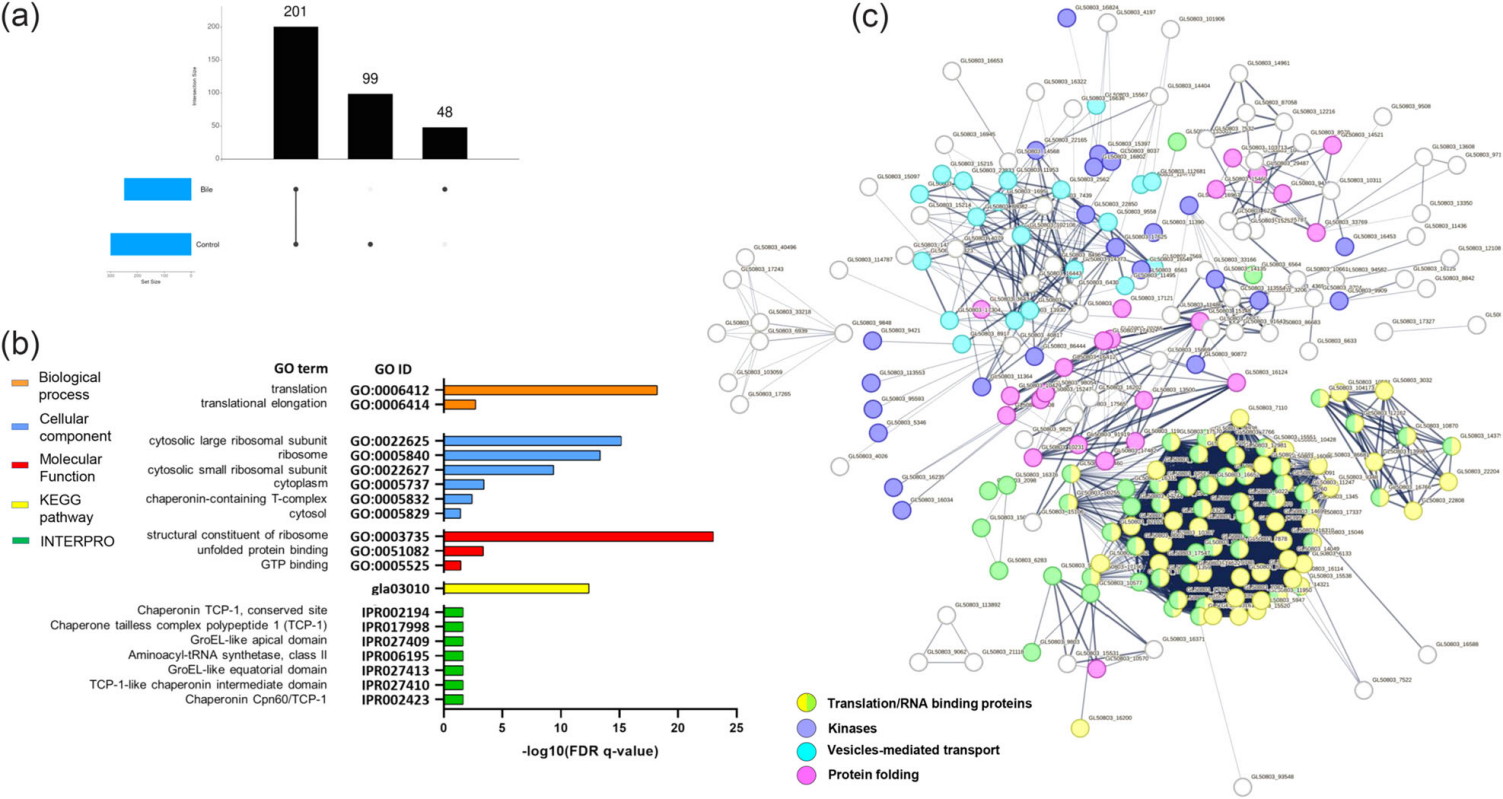
Proteomic analysis of *G. duodenalis* EVs. *G. duodenalis* isolate NF EVs protein content was analyzed using liquid chromatography with tandem mass spectrometry (LC–MS/MS). (a) Upset plot representation of *Giardia* EVs protein in Control and bile‐induced EVs; (b) GO, INTERPRO and KEGG pathway term enrichments of *Giardia* EVs proteins (‐log10(FDR q‐value)) are shown as bar graphs; (c) Protein‐protein interaction network analysis of *Giardia* EVs proteins showed statistically significant (*P* < 0.05) enrichment of functional networks related to translation/RNA binding proteins, kinases, vesicle mediated transport and protein folding interaction clusters enriched.

To characterize the proteomic content of EVs, we conducted LC‐MS/MS analyses of EVs isolated from *Giardia* trophozoites. We identified a total of 348 proteins (≥2 unique peptides) present in EVs across both control and bile conditions (Table S7). Upset plot representation showed 201 proteins shared hits between control and bile conditions, 99 unique protein hits in the control condition and 48 unique protein hits in the bile condition, respectively (Figure 3a). Canonical markers of EV, including 14‐3‐3 (GL50803_6430), Heat shock protein 70 (GL50803_17432) and 90 (GL50803_98054), Vacuolar sorting proteins (GL50803_14961, GL50803_23833, GL50803_114776, GL50803_112681), Rab11 (GL50803_1695) and Rab2a (GL50803_15567) were identified within the proteome. Gene ontology enrichment analysis of the EV proteome revealed elements involved in translation, unfolded protein binding, and GTP‐binding; KEGG pathway enrichment analysis revealed enrichments in Ribosome and Aminoacyl‐tRNA biosynthesis (Figure 3b). Protein‐protein interaction network analysis of the proteins within the EV proteome also reconfirmed enrichment in translation, tRNA ‐aminoacylation, chaperone complex and protein folding interaction clusters (Figure 3c). Interestingly, the analyses identified seven DEAD Box helicases (GL50803_16376, GL50803_2098, GL50803_34684, GL50803_6283, GL50803_10255), which are ATP‐dependent RNA chaperones that, along with the RNA they bind to, can phase‐separate into membrane‐less granules and stabilize the RNA in response to stress such as heat shock in model organisms. Analyses of the EV contents also identified previously characterized *Giardia* virulence factors such as cathepsin B cysteine proteases, arginine deaminase, tenascins, and variant surface proteins (Table S7). We also observed protein‐modifying enzymes, such as protein kinases (Ser/Thr and NEK).

### 3.4 *G. duodenalis* EVs increase bacterial swimming motility and alter biofilm formation

**FIGURE 4:**
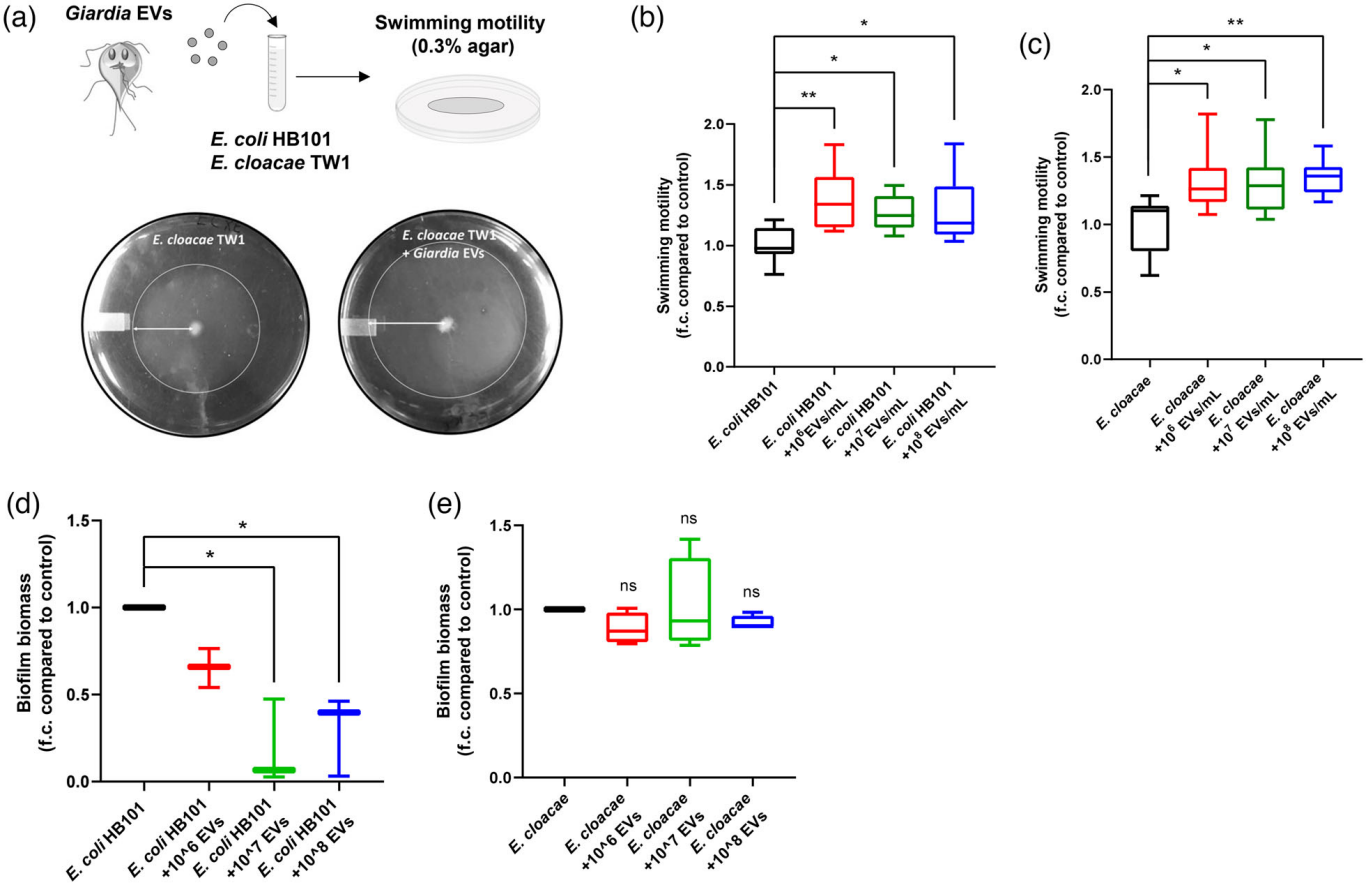
*G. duodenalis* EVs increase bacterial swimming motility and alter biofilm formation. (a) Bacteria were incubated for 24 h at 37°C with different concentrations of bile‐induced *Giardia* EVs (10^6^, 10^7^ and 10^8^ EVs/mL). Control group received vehicle (XE buffer). Swimming motility assays were conducted on 0.3% agar plate. Measurements of halo sized were done at Day 3 using Image J software. (b) Swimming motility of *E. coli* strain HB101 and (c) *E. cloacae* TW1 following exposure to *G. duodenalis* EVs. (c, d) Biofilm formation of *E. coli* strain HB101 (c) and *E. cloacae* TW1 (d) following exposure to *G. duodenalis* EVs. Bacteria were incubated with different concentrations of bile induced *Giardia* EVs (10^6^, 10^7^ and 10^8^ EVs/mL) for 24 h at 37°C. Control group received vehicle (XE buffer). After 24 h exposure, bacteria were inoculated into 96 well plate with Calgary Biofilm Device to assess their biofilm formation. After 48 h incubation, biofilm biomass was measured using crystal violet staining; O.D. was measured at 550 nm. Results are expressed as mean ± SEM. *n* = 4–6 per group. * *p* < 0.05, ** *p* < 0.01.

Additional experiments then assessed whether exposure to *Giardia* EVs could alter the behaviour or growth of bacteria. *E. coli* HB101 and *E. cloacae* TW1 were incubated for 24 h with increasing EV concentrations, and bacterial swimming motility was assessed on low‐viscosity agar plates (0.3% agar) (Figure 4a). The swimming motility of *E. coli* HB101 was significantly increased when treated with 10^6^ EVs/mL (*p* < 0.01), 10^7^ EVs/mL (*p* < 0.05), and 10^8^ EVs/mL (*p* < 0.05), respectively (Figure 4b). Similarly, the swimming motility of *E. cloacae* TW1 was increased with 10^6^ EVs/mL (*p* < 0.05), 10^7^ EVs/mL (*p* < 0.05), and 10^8^ EVs/mL (*p* < 0.01) (Figure 4c). The biofilm‐forming ability of *E. coli* HB101 and *E. cloacae* TW1 was then assessed using the Calgary Biofilm Device (CBD) with increased concentrations of *G. duodenalis* EVs. *E. coli* HB101 *and E. cloacae* TW1 were seeded in the CBD system and grown in aerobic conditions for 48 h at 37°C. *G. duodenalis* EVs at 10^7^ EVs/mL (*p* < 0.05) and 10^8^ EVs/mL (*p* < 0.05) significantly decreased the biofilm‐forming ability of *E. coli* HB101 (Figure 4d). In contrast, increasing concentrations of *Giardia* EVs (10^6^ EVs/mL, 10^7^ EVs/mL, and 10^8^ EVs/mL) did not change biofilm formation by *E. cloacae* TW1 (Figure 4e).

Further experiments also demonstrated that *Giardia* EVs inhibit the growth of bacteria by exhibiting bacteriostatic effects against *E. coli* HB101 at 10^6^ EVs/mL (*p* < 0.01), 10^7^ EVs/mL (*p* < 0.001), and 10^8^ EVs/mL (*p* < 0.001) concentrations. Higher concentrations (10^8^ EVs/mL) of *G. duodenalis* EVs also induced bacteriostasis in *E. cloacae* TW1 (*p* < 0.01), while no effects were observed with 10^6^ EVs/mL and 10^7^ EVs/mL (Figure S1). Bacteriostatic effects of *G. duodenalis* EVs were observed at the end of the exponential phase for each isolate.

### 3.5 *Giardia* EVs co‐localize with *E. coli* HB101 and *E. cloacae* TW1

**FIGURE 5:**
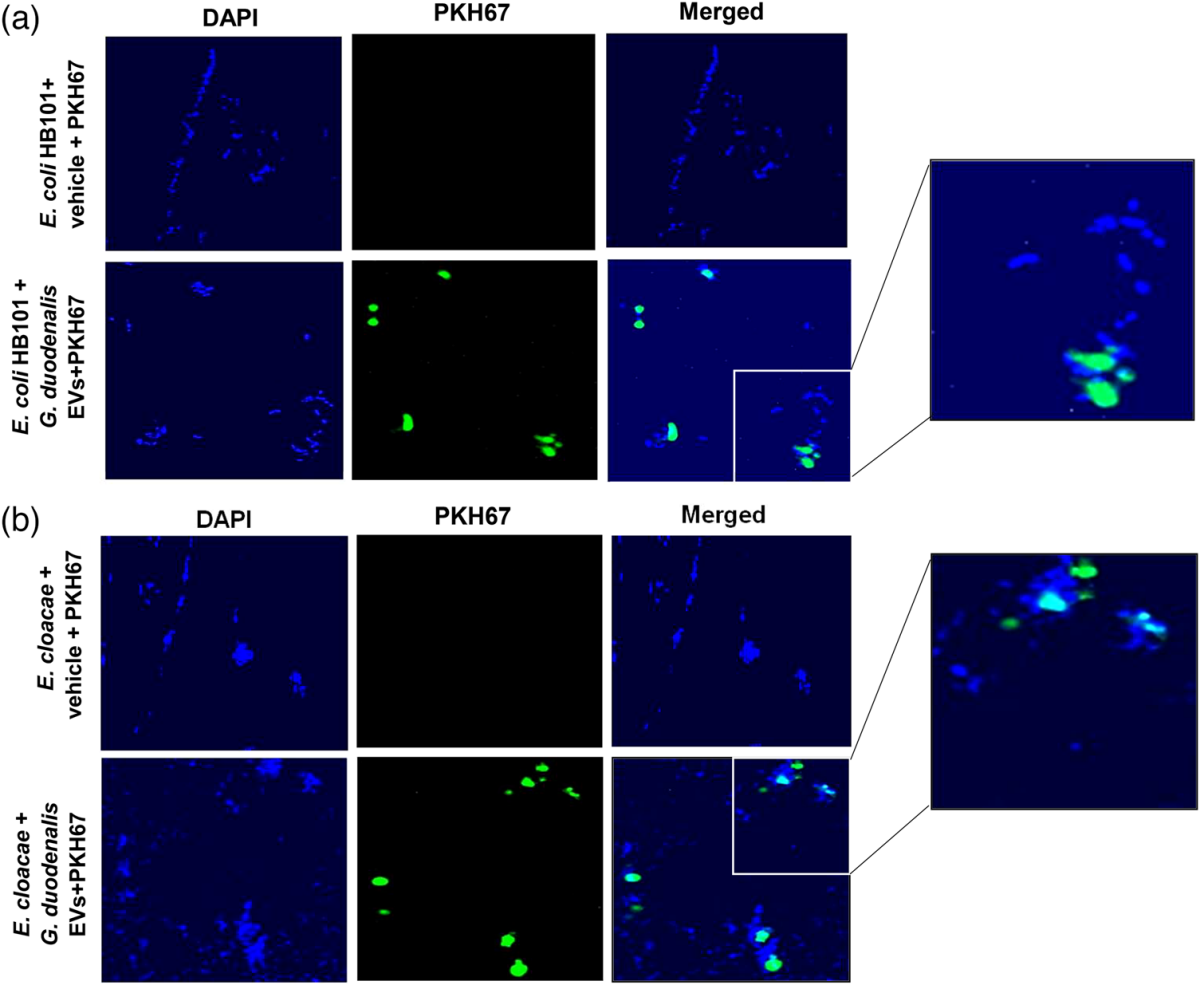
*G*. *duodenalis* EVs co‐localize with *E. coli* HB101 and *E. cloacae* TW1. *G. duodenalis* EVs were labelled using PKH67 dye. Pre‐labelled EVs were co‐cultured with either (a) *E. coli* strain HB101 and (b) *E. cloacae* TW1 for 1 hour. Bacteria were identified by DNA staining using DAPI (blue). Bacteria were probed for immunofluorescence staining of *Giardia* EVs (green). The PKH67‐vehicle control groups represent *E*. *coli* strain HB101, or *E. cloacae* TW1 pre‐treated with PKH67‐stained vehicle (XE buffer).

**FIGURE 6:**
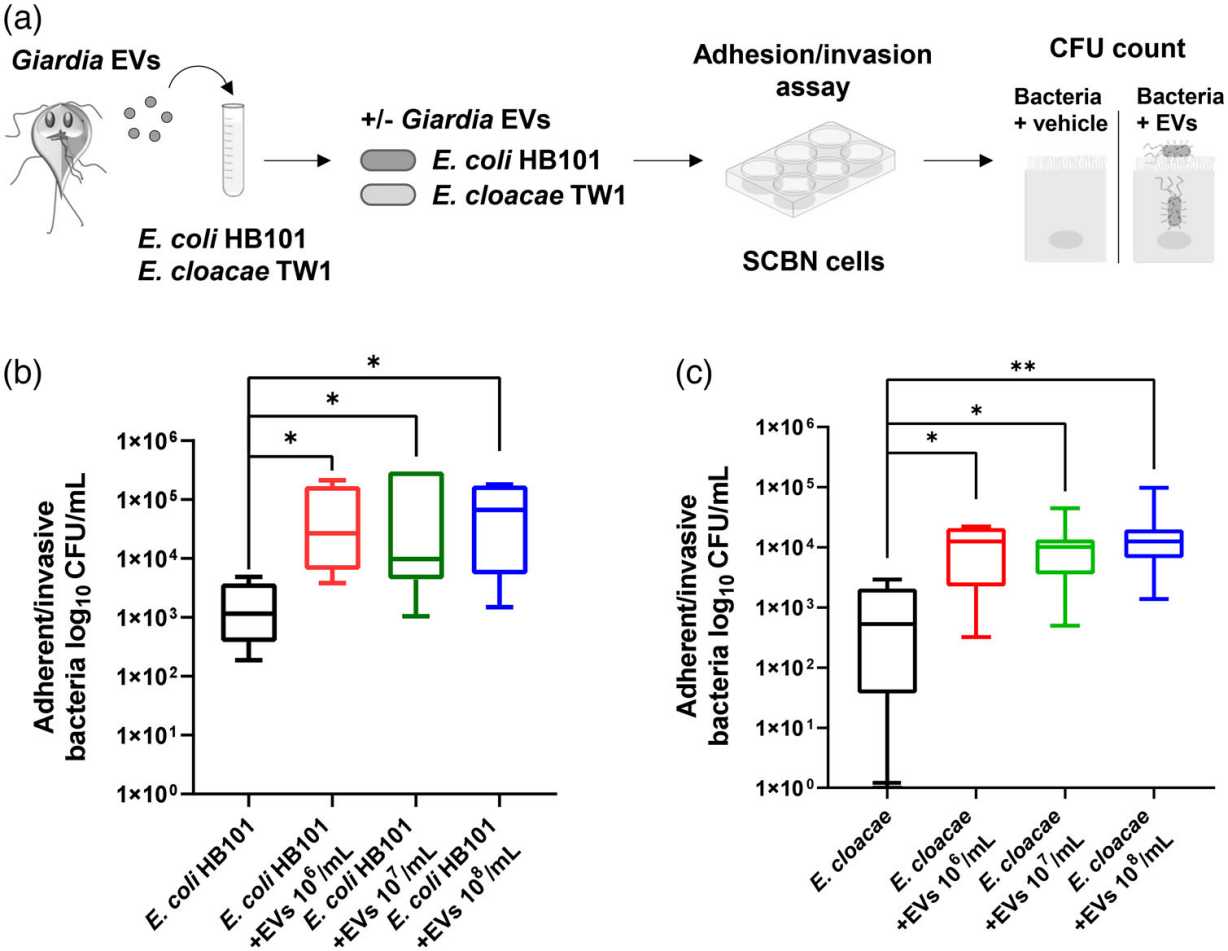
Bacterial adhesion to intestinal epithelial cells is enhanced by *G. duodenalis* EVs. (a) *E. coli* strain HB101 and *E. cloacae* TW1 were incubated with different concentrations of bile induced *G. duodenalis* EVs (10^6^, 10^7^ and 10^8^ EVs/mL) for 24 h at 37°C. Control groups received vehicle (XE buffer). Intestinal epithelial cells (SCBN) were infected with EV‐treated *E. coli* strain HB101 and *E. cloacae* TW1 (MOI = 100:1) for 3 h. Cells were washed and permeabilize with saponin. Adhering/invading bacteria were enumerated on LB and BHI agar plates, for (b) *E. coli* HB101 and (c) *E. cloacae* TW1, respectively. *n* = 4–6 per group. Results are expressed as mean ± SEM. *n* = 4–6 per group. * *p* < 0.05, ** *p* < 0.01 compared with the control group (vehicle).

To determine whether EVs could directly interact with the bacterial outer membrane, *G. duodenalis* EVs were pre‐labelled with a lipophilic fluorescent dye PKH67, which emits fluorescence only when combined with a cell membrane. In addition, bacterial genomic DNA was stained using DAPI. Merged images of PKH67 fluorescence and DAPI provided clear evidence that *G. duodenalis* EVs co‐localize with *E. coli* HB101, while no signal was detected in untreated bacterial cells (Figure 5a). *G. duodenalis* EVs co‐localized with *E. cloacae* TW1 in a similar fashion (Figure 6b). Bacteria treated with either vehicle or PKH67 alone did not show fluorescence (Figure 5a,b).

### 3.6 *G. duodenalis* EVs increase *E. coli* HB101 and *E. cloacae* TW1 adhesion/invasion to intestinal epithelial cells

**FIGURE 7:**
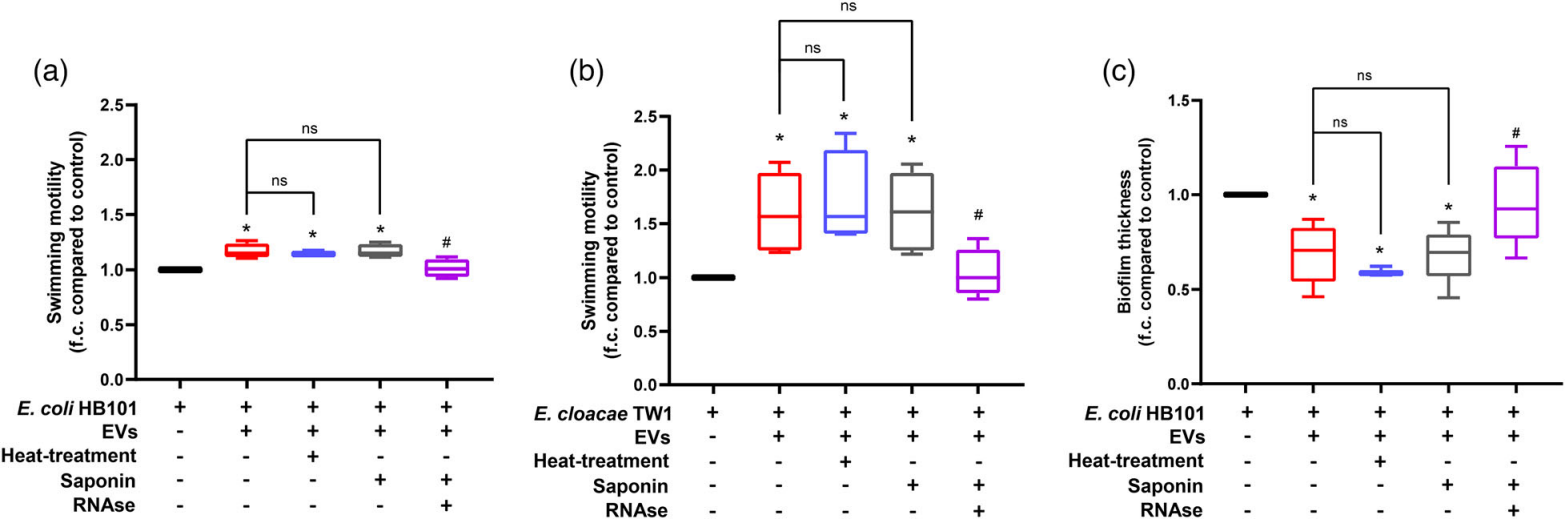
*G. duodenalis* EVs total RNAs but not proteins modulate bacterial swimming motility and biofilm formation. (a, b) Swimming motility of *E. coli* strain HB101 (a) and *E. cloacae* TW1 (b) following exposure to *G. duodenalis* EVs under various conditions. Bacteria were incubated for 24 h at 37°C with *G. duodenalis* EVs (10^8^ EVs/mL), saponin‐treated EVs (10^8^ EVs/mL), RNase‐treated EVs (10^8^ EVs/mL; pre‐treated with saponin) or heat‐treated EVs (10^8^ EVs/mL). Control group received vehicle (XE buffer). Swimming motility assays were conducted on 0.3% agar plate. Measurements of halo sized were done at Day 3 using Image J software. Results are expressed as mean ± SEM. *n* = 3–4 per group. * *p* < 0.05 compared with control group (vehicle); # *p* < 0.05 compared with RNase‐treated group. (c) Biofilm formation of *E. coli* strain HB101 following exposure to *G. duodenalis* EVs under various conditions. Bacteria were incubated for 24 h at 37°C with *G. duodenalis* EVs (10^8^ EVs/mL), RNase‐treated EVs (10^8^ EVs/mL) or heat‐treated EVs (10^8^ EVs/mL). Control group received vehicle (XE buffer). After 24 h exposure, bacteria were inoculated into 96 well plate with Calgary Biofilm Device to assess their biofilm formation. After 48 h of incubation, biofilm biomass was measured using crystal violet staining; O.D. was measured at 550 nm. Results are expressed as mean ± SEM. *n* = 3–4 per group. * *p* < 0.05 compared with the control group (vehicle); # *p* < 0.05 compared with *G. duodenalis* EVs, heat‐treated EVs, and Saponin‐treated EVs groups; ns = nonsignificant.

Bacterial adhesion/invasion to host cells represents an early stage of pathophysiology. As *Giardia* induces commensal microbiota bacteria to attach to and invade intestinal epithelia, additional experiments assessed whether EVs could be responsible for such a transformation. To assess bacterial adhesion/invasion to SCBN duodenal epithelial cells, *E. coli* HB101 and *E. cloacae* TW1 were incubated with EVs isolated from *Giardia* trophozoites for 24 h (Figure 6a). Bacteria were incubated with SCBN cells for 3 h, and adhesion/invasion was assessed using CFU counts on agar plates (Figure 6a). Adhesion/invasion of *E. coli* HB101 to SCBN cells was significantly increased when pre‐incubated with 10^6^ EVs/mL (*p* < 0.05), 10^7^ EVs/mL (*p* < 0.05), and 10^8^ EVs/mL (*p* < 0.05) concentrations, respectively, compared with unstimulated control bacteria (Figure 7b). Similarly, adhesion/invasion of *E. cloacae* TW1 to SCBN cells was also increased with 10^6^ EVs/mL (*p* < 0.05), 10^7^ EVs/mL (*p* < 0.05), and 10^8^ EVs/mL (*p* < 0.01) concentrations (Figure 6c).

### 3.7 The modulation of bacterial swimming motility and biofilm formation by *G. duodenalis* EVs is due to a thermoresistant and RNase‐sensitive cargo

To assess the mechanism underlying the effects of EVs on bacterial swimming motility, EVs were treated with saponin and RNase A. Saponin permeabilizes the membrane of EVs and allows the RNase A to enter the vesicles and digest the RNA content. In other studies, EVs were heated at 95°C for 15 min to denature proteins. Bacterial swimming motility was assessed as above. *G. duodenalis* EVs‐ increased swimming motility in bacteria was attenuated when EVs were pre‐treated with RNase A for both *E. coli* HB101 (*p* < 0.05) and *E. cloacae* TW1 (*p* < 0.05) (Figure 7A,B). In contrast, activation of bacterial swimming motility by EVs remained intact when *E. coli* HB101 and *E. cloacae* TW1 were exposed to heat‐treated *Giardia* EVs (Figure 7a,b).

Additional experiments assessed the effects of EVs RNAs and protein contents on bacterial biofilm formation. *Giardia* EVs ability to decrease *E. coli* HB101 biofilms formation was abolished when EVs were treated with RNase (Figure 7c). Heat treatment of *G. duodenalis* EVs did not reverse the effects of EVs on *E. coli* HB101 biofilm formation (Figure 7c). Together, these data indicate that *Giardia* EV RNA content may be responsible, at least in part, for the modulation of the swimming behaviour and biofilm formation of enteric commensal bacteria.

Further characterization of *G. duodenalis* EVs upon heat and RNase treatments was done using transmission electron microscopy. The TEM micrographs revealed that EVs heated at 95°C for 15 min showed similar spherical morphology and lipid bilayer compared with untreated EVs (Figure S2). No noticeable change in size range was observed. The effect of RNase on EVs integrity was then observed. EVs were permeabilized with saponin and treated with RNase for 30 min before column purification. TEM micrographs revealed a change in the morphology and size distribution of the EVs following saponin+RNase A treatment. In particular, the overall abundance of EVs larger than 100 nm in diameter was reduced compared to untreated and heat‐treated groups, confirming the RNase‐sensitive nature of *Giardia* EVs (Figure S2).

### 3.8 The increased bacterial adherence/invasion to SCBN cells induced by *G. duodenalis* EVs is due to a thermoresistant and RNase‐sensitive cargo

**FIGURE 8:**
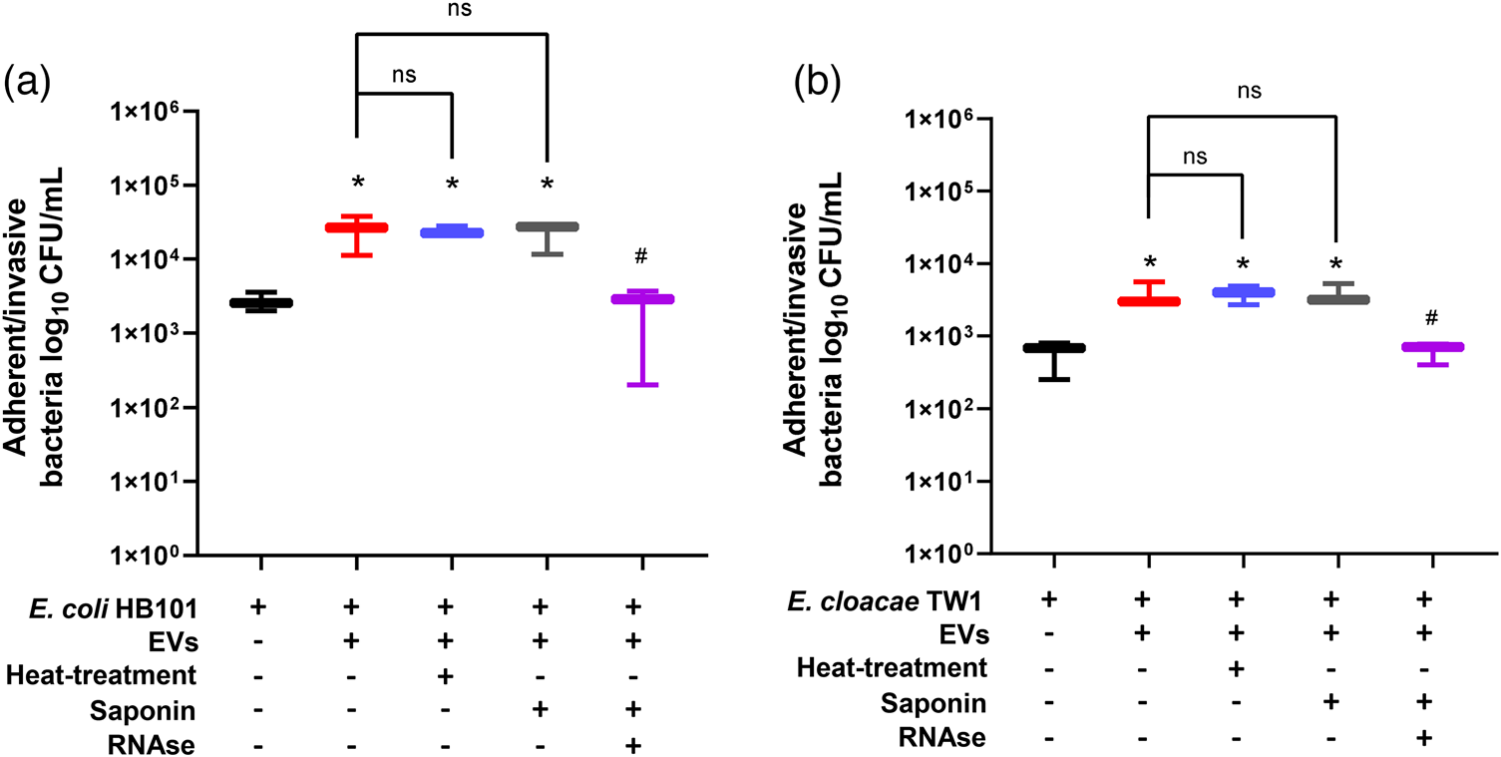
*G. duodenalis* EVs total RNAs but not proteins promote bacterial adhesion to intestinal epithelial cells. *E. coli* strain HB101 (a) and *E. cloacae* TW1 (b) were incubated with bile‐induced *G.duodenalis* EVs (10^8^ EVs/mL), saponin‐treated EVs (10^8^ EVs/mL), RNase‐treated EVs (10^8^ EVs/mL; pre‐treated with saponin) or heat‐treated EVs (10^8^ EVs/mL) for 24 h at 37°C. Control groups received vehicle (XE buffer). Intestinal epithelial cells (SCBN) were infected with EV‐treated *E. coli* strain HB101 (a) and *E. cloacae* TW1 (b) (MOI = 100:1) for 3 hours. Cells were washed and permeabilize with saponin. Adhering/invading bacteria were enumerated on LB and BHI agar plates, for *E. coli* HB101 and *E. cloacae* TW1, respectively. *n* = 4–6 per group. Results are expressed as mean ± SEM. *n* = 3 per group. * *p* < 0.05 compared with the control group (vehicle); # *p* < 0.05 compared with *G. duodenalis* EVs, heat‐treated EVs, and Saponin‐treated EVs groups; ns = non significant.

Pre‐treatment of *G. duodenalis* EVs with RNase abolished the increased cell adhesion/invasion to SCBN cells by both *E. coli* HB101 (*p* < 0.05) (Figure 8a) and *E. cloacae* TW1 (*p* < 0.05) (Figure 8b). Conversely, heat treatment and saponin treatment alone did not reverse the pro‐adhesion and pro‐invasion effects of EVs (Figure 8a,b). These data indicate that *G. duodenalis* EVs RNA contents increase bacterial adherence to intestinal epithelial cells.

### 3.9 *E. coli* mRNA‐*Giardia* sRNAs interaction network analysis

**FIGURE 9:**
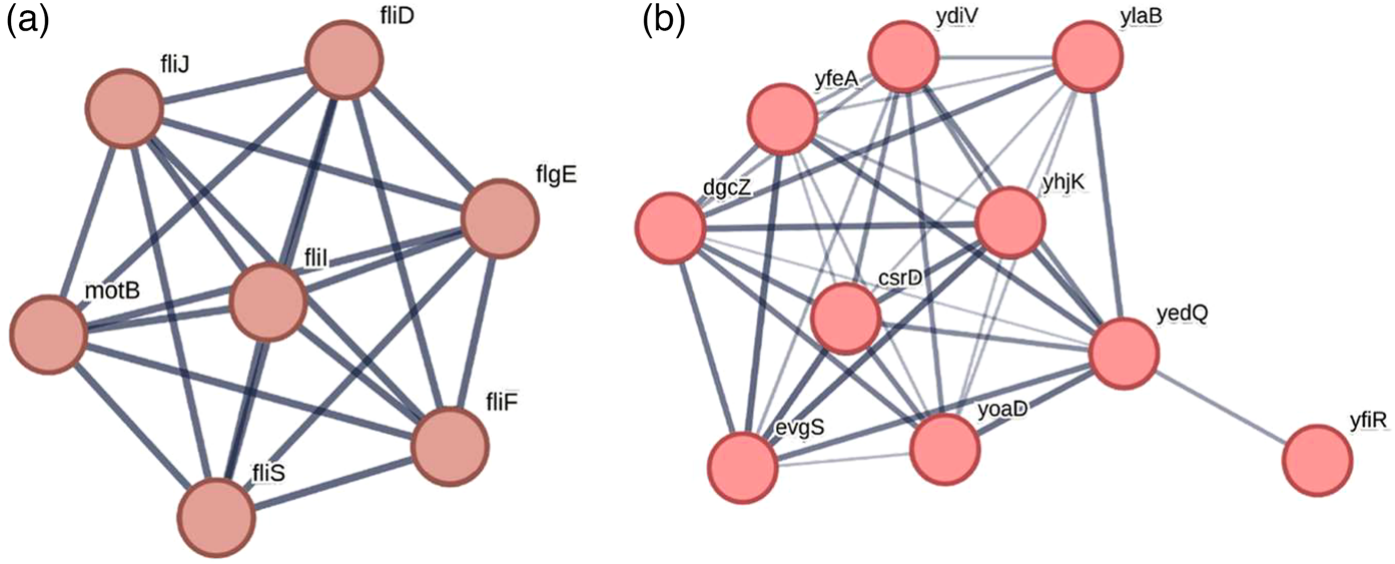
*E. coli* HB101 mRNA‐*Giardia* EVs sRNAs interaction network analysis. Protein–protein interaction network corresponding to (a) flagellar motility enriched and (b) biofilm formation enriched clusters (*p* < 1*10^−16^) within *E. coli* mRNA interactome of *Giardia* sRNAs.

Given the absence of DICER in *E. coli* and *E. cloacae* TW1, we hypothesized that the *Giardia* EVs small RNAs act as post‐transcriptional regulators of mRNAs in *Enterobacteriaceae* species. *In silico* predicted mRNA targets of the sRNAs identified in bile‐induced *Giardia* EVs showed significant enrichment of mRNAs that encode proteins involved in flagellar biosynthesis and swimming motility FliD, FlgE, MotB, FliS, FliF, FliI and FliJ (*p* < 10^16^) (Figure 9a) as well as cyclic‐diGMP signalling and biofilm formation such as YedQ, YfeA, YlaB, DgcZ, YedQ, YhjK and YfiR (Sanchez‐Torres et al., 2011), and metal ions uptake (i.e., iron, zinc) and bacterial invasion such as YdiV and YoaD (*p* < 10^16^) (Figure 9b) (Zhang et al., 2020).

## 4 DISCUSSION

*G. duodenalis* causes disruptions in the gut microbiota that are key contributors to the pathophysiology of giardiasis (Allain & Buret, 2020; Beatty et al., 2017). *Giardia*‐induced microbiota disruptions also occur at sites devoid of colonizing trophozoites. However, the mechanisms responsible for leading to these abnormalities remain obscure. The present studies examined whether extracellular vesicles (EVs) released by *G. duodenalis* may mediate pathogenic effects on commensal bacteria using *E. coli* and *E. cloacae* TW1 as target models, both well‐recognized opportunistic pathogens of the gut microbiota. Recent studies have examined the biogenesis of *G. duodenalis* EVs and their role in modulating various aspects of host‐parasite interactions (Evans‐Osses et al., 2017; Gavinho et al., 2020; Moyano et al., 2019; Natali et al., 2023; Zhao et al., 2021). However, the role of EVs in parasite‐microbiota interactions has yet to be explored. Our studies demonstrate that *Giardia* EVs interact with bacterial membranes, increase bacterial swimming motility, inhibit bacterial growth, and alter biofilm formation. Consistent with the species‐selective disruptions observed in dysbiotic microbiota, the effects of EVs were partly isolate‐dependent. The effects were sensitive to RNAse but not to heat treatment. The data then revealed that the RNA cargo of *G. duodenalis* EVs was also responsible for increased bacterial adherence to intestinal epithelial cells, a well‐established factor in the pathogenesis of a variety of intestinal disorders and a known virulence factor of enteric pathobionts (Backhed et al., 2012; Buret et al., 2019; Sartor & Wu, 2017).

Using state‐of‐the‐art methodologies, including nanoparticles track analysis (NTA) and transmission electron microscopy (TEM), our first goal was to characterize the morphology of *Giardia* EVs. Our findings suggest that *G. duodenalis* isolate NF trophozoites secrete lipid bilayer‐coated large vesicles (200–270 nm; macrovesicles‐like particles) which are slightly larger than previously observed EVs from other *Giardia* isolates (Gavinho et al., 2020; Moyano et al., 2019; Natali et al., 2023). We then assessed whether EV production is altered upon exposure to chemical stimuli. Exposure to bile (5 mg/mL) dramatically augmented the production of EVs by *Giardia*. CaCl_2_ (1 mM), a known activator of EV production (Evans‐Osses et al., 2017), also increased EVs production to a lesser extent. No morphological alterations of *Giardia* trophozoites were observed in bile‐ and CaCl_2_‐treated groups. The exact mechanism by which bile enhances EVs production warrants further investigation. Nonetheless, this finding is physiologically important as trophozoites are exposed to large quantities of bile when they colonize the upper small intestine of the host upon excystation.

The possible implication of EVs in cross‐kingdom crosstalk has been articulated recently, albeit never within the gut microbiome (Cai et al., 2019; Lee, 2019). *E. coli* HB101 is a well‐known laboratory strain derived from *E. coli* K12 that has minimal pathogenic effects on host cells (Kuhnert et al., 1995). *E. cloacae* is a commensal bacterium in the human gut microbiome and can be an opportunistic pathogen (Keller et al., 1998). Previous reports have shown that EVs derived from milk can be taken up by bacteria and promote their growth (Yu et al., 2019). In contrast, urinary exosomes exhibit bacteriostatic effects on bacteria and can result in their lysis (Hiemstra et al., 2014). Our data demonstrate for the first time that *G. duodenalis* EVs can alter the growth, behaviour, and virulence of *Enterobacteriaceae* species. *G. duodenalis* EVs also inhibited the biofilm‐forming ability of *E. coli* HB101 but not of *E. cloacae* TW1. The results highlight an evident interplay between *Giardia* and members of gut commensal bacteria. These interactions are at least in part bacteria‐specific, consistent with the alterations in relative bacterial abundance observed during *Giardia*‐induced microbiota dysbiosis (Allain et al., 2021; Beatty et al., 2017; Fekete et al., 2020). In the gut, commensal bacterial communities form poly‐microbial structures called biofilms, which help microbes withstand shear forces and protect them from luminal contents (Buret et al., 2019). This microenvironment allows for cooperative interactions within the biofilm via quorum sensing (Dunne, 2002). By decreasing the ability of *Enterobacteriaceae* species to form biofilms, *Giardia* EVs may destabilize this commensal population, as fragmentation of the biofilm structure is known to facilitate the release of adherent, invasive pathobionts (Beatty et al., 2017; Buret & Allain, 2023; Buret et al., 2019; Motta et al., 2018). Our findings support the hypothesis that essential components of the microbiota dysbiosis associated with *Giardia* infection are induced via its EVs.

The present findings also demonstrate that *G. duodenalis* EVs promote the adhesion and invasion of *E. coli* and *E. cloacae* TW1 to intestinal epithelial cells. Exposure to *Campylobacter jejuni*, another common enteropathogen, also increases adherence and transcellular translocation of otherwise commensal bacteria (Kalischuk et al., 2009; Reti et al., 2015). *C. jejuni* induces flagellar and fimbrial genes in *E. coli* HB101, causing an augmented adhesion force to epithelial cells measurable by atomic force microscopy (Reti et al., 2015). These mechanisms are critical contributors to disease pathogenesis in the gut (Backhed et al., 2012; Buret & Allain, 2023; Buret et al., 2019; Sartor & Wu, 2017). Ingestion of naïve *E. coli* HB 101 and human microbiota from healthy donors does not affect *Caenorhabditis elegans* survival, but upon prior exposure to *Giardia*, they become lethal to the nematode (Gerbaba et al., 2015). The mechanisms whereby *Giardia* EVs transform commensal bacteria into pathogenic pathobionts deserve further investigation.

Our analyses revealed that various types of sRNAs, namely (1) miRNAs, (2) siRNAs (specifically endo‐siRNAs), (3) tRNA‐derived small RNAs (tRFs), and (4) rRNA‐derived small RNAs (rRFS) are packaged in *Giardia* EVs. The findings also showed that sRNAs and miRNAs are recruited differently in *Giardia* EVs in response to bile. Hence, our findings suggest that distinct small RNA species may be recruited into *Giardia* EVs in response to diverse physiological stimuli. Furthermore, our work suggests that *Giardia* EVs may participate in small RNA (sRNA)‐mediated gene regulation, a conserved regulatory mechanism for gene expression in both prokaryotes and eukaryotes. This is consistent with the results obtained in studies investigating the sRNA content of other prominent protists like *Trypanosoma* and *Leishmania*, where EVs are enriched in rRNA and tRNA‐derived sRNAs (Bayer‐Santos et al., 2014; Lambertz et al., 2015).

Studies of parasites like *Trichomonas vaginalis*, *Plasmodium* spp., *Trypanosoma* sp. and *Leishmania* spp. have shown that these protists use EVs as a cell‐cell communication system (Cheng et al., 2019; Marcilla et al., 2014; Nievas et al., 2020; Wu et al., 2018). The cargo of these EVs can modulate motility, tissue tropism, drug resistance and differentiation within these organisms (Cheng et al., 2019; Marcilla et al., 2014; Nievas et al., 2020; Wu et al., 2018). Using small RNA Sequencing and *in silico* miRNA‐ 3′UTR interaction network mapping analyses, the findings described here point to the possibility that the *Giardia* EV cargo may regulate *Giardia* differentiation and motility. Several endo‐siRNAs significantly differentially transcribed within EVs derived from bile‐treated trophozoites were previously identified in encysting *Giardia* trophozoites (Liao et al., 2014). Similarly, *in silico* miRNA‐ 3′UTR interaction network mapping showed that microtubule network complexes and ATP‐binding proteins are a putative regulatory niche for *Giardia* EVs miRNAs. These novel findings hint that *Giardia* EVs may modulate gene expression networks in *Giardia*. To our knowledge, this study is the first study to present an analysis of the miRNAs packaged in EVs with their potential target genes in *Giardia*. More research, including *Giardia‐*derived EV‐exposed single‐cell transcriptomics of *Giardia* sub‐populations, will give us clues on gene expression networks changing within *Giardia* trophozoites in response to EVs. Northern blot and AGO‐CLASH experiments must also be employed to assess whether and how these miRNAs regulate the parasite's microtubule‐dependent cellular motility.

A key observation from this study is that the sRNAs cargo in *Giardia* EVs can be delivered to other organisms in a novel type of trans‐kingdom cross‐talk. Predicted bacterial targets for sRNA suggest that EVs can interfere with the expression of genes involved in flagellar biosynthesis, uptake of metal ions such as iron, motility, cyclic‐di‐GMP signalling and biofilm formation. Whether bacterial gene expression patterns are re‐wired post‐fusion with *Giardia* EVs through sRNA‐mediated post‐transcriptional regulation remains to be explored. Taken together, our findings illustrate the importance of RNA contents in *Giardia* EVs and highlight their potential as mediators of microbial‐microbial and host‐parasite interactions. Furthermore, as miRNAs are well conserved across species, these observations carry biological significance far beyond the study of giardiasis.

Small RNA sequencing was further complemented by proteomic analysis of *Giardia* EVs using LC‐MS/MS. The findings reveal that *Giardia* EVs contain primarily translational proteins such as ribosomes, amino‐acyl tRNA synthetases, and ATP‐dependent RNA chaperones, namely DEAD Box helicases, as well as protein‐folding chaperones (e.g., Dna J, Heat shock proteins and proteasome subunits). Interestingly, the proportion of translational proteins and protein‐folding chaperones was increased in EVs derived from bile‐treated trophozoites. High‐resolution mass spectrometry has provided quantifiable evidence at protein level for these protein families in EVs and showed their differential expression during bile‐mediated *Giardia* differentiation (Balan et al., 2021). Gene Ontology analysis revealed that most of these were translational proteins involved in RNA binding. Past observations suggest that these proteins can end up in EVs with RNA molecules as RNA–ribonucleoprotein complexes (Statello et al., 2018). These complexes could also be essential in transporting RNAs into EVs and maintaining RNAs inside EVs, which can be safely transported into a recipient cell (Statello et al., 2018). In addition, the proteome data identified known regulators of the thermal stability of both RNA and proteins recruited into the EV, suggesting enhanced protection of the EV cargo. Our proteomic analyses also identified major *Giardia* virulence factors in EVs, including cathepsin B cysteine proteases, arginine deaminases, tenascins, and variant surface proteins.

The effects of *Giardia* EVs on bacterial motility, biofilm formation and epithelial adhesion/invasion were heat‐resistant but reversed upon treatment with saponin RNase A. Saponin is a surfactant molecule that forms complexes with cholesterol in EV cell membranes and generates pores, increasing membrane permeability (Podolak et al., 2010). Treatment with saponin alone (1%) did not result in a loss of the biological effect of the EVs. This permeabilization allows RNase A to enter EVs and digest most RNA content. Incubation of EVs with RNAse without saponin does not result in RNA degradation (Chiou et al., 2018; Enderle et al., 2015). Treatment with RNase A and saponin has been shown to digest numerous small RNAs, including rRNA‐derived sRNAs and miRNAs (Chiou et al., 2018). Similar observations showed that miRNAs from colonocyte‐derived exosomes are protected from digestion by RNase, while free fecal miRNAs are rapidly degraded (Koga et al., 2011).

Though heat treatment denatures most proteins' secondary, tertiary, and quaternary structures, the effect of *G. duodenalis* EVs on enterobacteria was not reversed by heating at 95°C. While the role of heat‐stable proteins and peptides cannot be excluded, the RNA content of *G. duodenalis* EVs appears to be resistant to high temperatures, consistent with previous observations that thermo‐treated EVs could still exert biological activity (Rodriguez & Kuehn, 2020; Salomon et al., 2016, 2014). Moreover, miRNAs isolated from degraded RNA preparations from human tissues and cell samples show robust stability even when incubated at 80°C (Jung et al., 2010). These observations demonstrate that EVs RNA cargo, particularly miRNAs, has high stability.

In summary, our results indicate that *G. duodenalis* EVs‐mediated effects on *Enterobacteriaceae* species are due, at least in part, to the thermoresistant RNA cargo of these EVs. Recent research on EVs RNA contents has focused on miRNAs because of their potential to modulate gene expression in recipient cells. The present study illustrates how protozoan EVs may also affect bacterial behaviour and virulence in a manner similar to a pioneering observation that showed how miRNAs from a human host could enter bacteria, regulate their growth, and modulate the expression of various genes (Liu et al., 2016). Our analyses identified potential miRNAs and sRNAs differentially recruited into *G. duodenalis* EVs, and *in silico* studies suggest miRNAs are involved in targeting cytoskeletal organisations within *Giardia*. These studies also identified other RNAs in *Giardia* EVs, such as rRNA and tRNA‐derived small RNAs, as well as endo‐siRNAs, which at the *in silico* level target bacterial genes regulating motility and pathogenicity. Hence, this study is the first to establish the probable role of EV sRNA and miRNA in the post‐transcriptional regulation of *Giardia*‐microbiota interactions. Previous studies in mammals and yeast have revealed that rRNA‐derived small RNAs are functional molecules that play important roles in transcriptional regulation (Cam et al., 2005). Similarly, tRNA‐derived sRNAs have been implicated in mRNA destabilization and translation and can act as retro‐elements of reverse transcriptional and post‐transcriptional processes (Cao et al., 2020). Our findings highlight for the first time the importance of the RNA content in *G. duodenalis* EVs in modifying the phenotype of commensal bacteria and implicating EVs in a novel trans‐kingdom cross‐talk in the gut. This cross‐talk also appears to be protected from environmental stressors *via* its chaperones‐like contents. The findings pave the way towards future research into the fundamental biology of cell‐cell interactions and gene regulation during parasitism and microbiota dysbiosis, and point to new mechanisms that may play a key role in gastrointestinal pathophysiology.

## AUTHOR CONTRIBUTIONS

**Affan Siddiq**: Conceptualization; data curation; formal analysis; methodology; visualization; writing—original draft; writing—review & editing. **George Dong**: Data curation; formal analysis; methodology; writing—original draft; writing—review & editing. **Balu Balan**: Data curation; formal analysis; methodology; software; visualization; writing—original draft; writing—review & editing. **Luke G. Harrison**: Conceptualization; data curation; methodology. **Aaron Jex**: Conceptualization; formal analysis; investigation; methodology; validation; writing—review & editing. **Martin Olivier**: Conceptualization; data curation; formal analysis; investigation; methodology; writing—original draft; writing—review & editing. **Thibault Allain**: Conceptualization; data curation; formal analysis; investigation; methodology; supervision; visualization; writing—original draft; writing—review & editing. **Andre G. Buret**: Conceptualization; formal analysis; funding acquisition; investigation; methodology; project administration; supervision; validation; writing—original draft; writing—review & editing.

## CONFLICT OF INTEREST STATEMENT

All co‐authors have seen and agree with the contents of the manuscript and there is no conflict of interest.

## Supporting information

Supplementary Information

Supplementary Information

Supplementary Information

Supplementary Information

Supplementary Information

Supplementary Information

Supplementary Information

Supplementary Information

Supplementary Information

## References

[jex2109-bib-0001] Allain, T. , & Buret, A. G. (2020). Pathogenesis and post‐infectious complications in giardiasis. Advances in Parasitology, 107, 173–199. 32122529 10.1016/bs.apar.2019.12.001

[jex2109-bib-0002] Allain, T. , Fekete, E. , & Buret, A. G. (2019). Giardia cysteine proteases: The teeth behind the smile. Trends in Parasitology, 35, 636–648. 31279655 10.1016/j.pt.2019.06.003

[jex2109-bib-0003] Allain, T. , Fekete, E. , Sosnowski, O. , Desmonts De Lamache, D. , Motta, J. P. , Leger, D. , Feener, T. , Reimer, R. A. , & Buret, A. G. (2021). High‐fat diet increases the severity of Giardia infection in association with low‐grade inflammation and gut microbiota dysbiosis. Scientific Reports, 11, 18842. 34552170 10.1038/s41598-021-98262-8 PMC8458452

[jex2109-bib-0004] Amat, C. B. , Motta, J. P. , Fekete, E. , Moreau, F. , Chadee, K. , & Buret, A. G. (2017). Cysteine protease‐dependent mucous disruptions and differential mucin gene expression in Giardia duodenalis infection. American Journal of Pathology, 187, 2486–2498. 28823873 10.1016/j.ajpath.2017.07.009

[jex2109-bib-0005] Andrews, S. (2010). “FastQC: A Quality Control Tool for High Throughput Sequence Data [Online] ”.).

[jex2109-bib-0006] Atayde, V. D. , Da Silva Lira Filho, A. , Chaparro, V. , Zimmermann, A. , Martel, C. , Jaramillo, M. , & Olivier, M. (2019). Exploitation of the Leishmania exosomal pathway by Leishmania RNA virus 1. Nature Microbiology, 4, 714–723. 10.1038/s41564-018-0352-y 30692670

[jex2109-bib-0007] Backhed, F. , Fraser, C. M. , Ringel, Y. , Sanders, M. E. , Sartor, R. B. , Sherman, P. M. , Versalovic, J. , Young, V. , & Finlay, B. B. (2012). Defining a healthy human gut microbiome: current concepts, future directions, and clinical applications. Cell Host & Microbe, 12, 611–622. 23159051 10.1016/j.chom.2012.10.012

[jex2109-bib-0008] Balan, B. , Emery‐Corbin, S. J. , Sandow, J. J. , Ansell, B. R. E. , Tichkule, S. , Webb, A. I. , Svard, S. G. , & Jex, A. R. (2021). Multimodal regulation of encystation in *Giardia duodenalis* revealed by deep proteomics. International Journal of Parasitology, 51, 809–824. 34331939 10.1016/j.ijpara.2021.01.008

[jex2109-bib-0009] Bartelt, L. A. , & Sartor, R. B. (2015). Advances in understanding Giardia: Determinants and mechanisms of chronic sequelae. F1000Prime Report, 7, 62. 10.12703/P7-62 PMC4447054 26097735

[jex2109-bib-0010] Bayer‐Santos, E. , Lima, F. M. , Ruiz, J. C. , Almeida, I. C. , & Da Silveira, J. F. (2014). Characterization of the small RNA content of *Trypanosoma cruzi* extracellular vesicles. Molecular and Biochemical Parasitology, 193, 71—74. 24583081 10.1016/j.molbiopara.2014.02.004 PMC4058860

[jex2109-bib-0011] Beatty, J. K. , Akierman, S. V. , Motta, J. P. , Muise, S. , Workentine, M. L. , Harrison, J. J. , Bhargava, A. , Beck, P. L. , Rioux, K. P. , Mcknight, G. W. , Wallace, J. L. , & Buret, A. G. (2017). Giardia duodenalis induces pathogenic dysbiosis of human intestinal microbiota biofilms. International Journal of Parasitology, 47, 311–326. 28237889 10.1016/j.ijpara.2016.11.010

[jex2109-bib-0012] Bhargava, A. , Cotton, J. A. , Dixon, B. R. , Gedamu, L. , Yates, R. M. , & Buret, A. G. (2015). Giardia duodenalis surface cysteine proteases induce cleavage of the intestinal epithelial cytoskeletal protein villin via myosin light chain kinase. PLoS One, 10, e0136102. 26334299 10.1371/journal.pone.0136102 PMC4559405

[jex2109-bib-0013] Bolger, A. M. , Lohse, M. , & Usadel, B. (2014). Trimmomatic: A flexible trimmer for Illumina sequence data. Bioinformatics, 30, 2114–2120. 24695404 10.1093/bioinformatics/btu170 PMC4103590

[jex2109-bib-0014] Buchel, L. A. , Gorenflot, A. , Chochillon, C. , Savel, J. , & Gobert, J. G. (1987). In vitro excystation of Giardia from humans: A scanning electron microscopy study. Journal of Parasitology, 73, 487–493. 3598798

[jex2109-bib-0015] Buck, A. H. , Coakley, G. , Simbari, F. , Mcsorley, H. J. , Quintana, J. F. , Le Bihan, T. , Kumar, S. , Abreu‐Goodger, C. , Lear, M. , Harcus, Y. , Ceroni, A. , Babayan, S. A. , Blaxter, M. , Ivens, A. , & Maizels, R. M. (2014). Exosomes secreted by nematode parasites transfer small RNAs to mammalian cells and modulate innate immunity. Nature Communications, 5, 5488. 10.1038/ncomms6488 PMC4263141 25421927

[jex2109-bib-0016] Buret, A. , Hardin, J. A. , Olson, M. E. , & Gall, D. G. (1992). Pathophysiology of small intestinal malabsorption in gerbils infected with Giardia lamblia. Gastroenterology, 103, 506–513. 1634068 10.1016/0016-5085(92)90840-u

[jex2109-bib-0017] Buret, A. , & Lin, Y. C. (2008). Genotypic characterization of an epithelial cell line for the study of parasite‐epithelial interactions. Journal of Parasitology, 94, 545–548. 18564760 10.1645/GE-1395.1

[jex2109-bib-0018] Buret, A. G. , & Allain, T. (2023). Gut microbiota biofilms: From regulatory mechanisms to therapeutic targets. Journal of Experimental Medicine, 220, e20221743. 36688957 10.1084/jem.20221743 PMC9884580

[jex2109-bib-0019] Buret, A. G. , Motta, J. P. , Allain, T. , Ferraz, J. , & Wallace, J. L. (2019). Pathobiont release from dysbiotic gut microbiota biofilms in intestinal inflammatory diseases: A role for iron? Journal of Biomedical Science, 26, 1. 30602371 10.1186/s12929-018-0495-4 PMC6317250

[jex2109-bib-0020] Cai, Q. , He, B. , Weiberg, A. , Buck, A. H. , & Jin, H. (2019). Small RNAs and extracellular vesicles: New mechanisms of cross‐species communication and innovative tools for disease control. PLoS Pathogy, 15, e1008090. 10.1371/journal.ppat.1008090 PMC6936782 31887135

[jex2109-bib-0021] Cam, H. P. , Sugiyama, T. , Chen, E. S. , Chen, X. , Fitzgerald, P. C. , & Grewal, S. I. (2005). Comprehensive analysis of heterochromatin‐ and RNAi‐mediated epigenetic control of the fission yeast genome. Nature Genetics, 37, 809–819. 15976807 10.1038/ng1602

[jex2109-bib-0022] Cao, J. , Cowan, D. B. , & Wang, D. Z. (2020). tRNA‐derived small RNAs and their potential roles in cardiac hypertrophy. Frontiers in Pharmacology, 11, 572941. 33041815 10.3389/fphar.2020.572941 PMC7527594

[jex2109-bib-0023] Ceri, H. , Olson, M. E. , Stremick, C. , Read, R. R. , Morck, D. , & Buret, A. (1999). The Calgary biofilm device: New technology for rapid determination of antibiotic susceptibilities of bacterial biofilms. Journal of Clinical Microbiology, 37, 1771–1776. 10325322 10.1128/jcm.37.6.1771-1776.1999 PMC84946

[jex2109-bib-0024] Cheng, W. J. , Jiang, H. , Dong, H. F. , & Liu, R. (2019). Advances in researches of exosomes and other extracellular vesicles in parasites and parasitic diseases. Zhongguo Xue Xi Chong Bing Fang Zhi Za Zhi, 31, 555—559. 31713395 10.16250/j.32.1374.2018162

[jex2109-bib-0025] Chin, A. C. , Teoh, D. A. , Scott, K. G. , Meddings, J. B. , Macnaughton, W. K. , & Buret, A. G. (2002). Strain‐dependent induction of enterocyte apoptosis by Giardia lamblia disrupts epithelial barrier function in a caspase‐3‐dependent manner. Infection and Immunigy, 70, 3673–3680. 10.1128/IAI.70.7.3673-3680.2002 PMC128105 12065509

[jex2109-bib-0026] Chiou, N. T. , Kageyama, R. , & Ansel, K. M. (2018). Selective export into extracellular vesicles and function of tRNA fragments during T cell activation. Cell Reports, 25, 3356–3370. e3354. 30566862 10.1016/j.celrep.2018.11.073 PMC6392044

[jex2109-bib-0027] Cotton, J. A. , Amat, C. B. , & Buret, A. G. (2015). Disruptions of host immunity and inflammation by *Giardia Duodenalis*: Potential consequences for co‐infections in the gastro‐intestinal tract. Pathogens, 4, 764–792. 26569316 10.3390/pathogens4040764 PMC4693164

[jex2109-bib-0028] Cotton, J. A. , Motta, J. P. , Schenck, L. P. , Hirota, S. A. , Beck, P. L. , & Buret, A. G. (2014). Giardia duodenalis infection reduces granulocyte infiltration in an in vivo model of bacterial toxin‐induced colitis and attenuates inflammation in human intestinal tissue. PLoS One, 9, e109087. 25289678 10.1371/journal.pone.0109087 PMC4188619

[jex2109-bib-0029] Dunne, W. M., Jr. (2002). Bacterial adhesion: Seen any good biofilms lately? Clinical Microbiology Reviews, 15, 155–166. 11932228 10.1128/CMR.15.2.155-166.2002 PMC118072

[jex2109-bib-0030] Enderle, D. , Spiel, A. , Coticchia, C. M. , Berghoff, E. , Mueller, R. , Schlumpberger, M. , Sprenger‐Haussels, M. , Shaffer, J. M. , Lader, E. , Skog, J. , & Noerholm, M. (2015). Characterization of RNA from exosomes and other extracellular vesicles isolated by a novel spin column‐based method. PLoS One, 10, e0136133. 26317354 10.1371/journal.pone.0136133 PMC4552735

[jex2109-bib-0031] Enright, A. J. , John, B. , Gaul, U. , Tuschl, T. , Sander, C. , & Marks, D. S. (2003). MicroRNA targets in Drosophila. Genome Biology, 5, R1. 14709173 10.1186/gb-2003-5-1-r1 PMC395733

[jex2109-bib-0032] Evans‐Osses, I. , Mojoli, A. , Monguio‐Tortajada, M. , Marcilla, A. , Aran, V. , Amorim, M. , Inal, J. , Borras, F. E. , & Ramirez, M. I. (2017). Microvesicles released from Giardia intestinalis disturb host‐pathogen response in vitro. European Journal of Cell Biology, 96, 131–142. 28236495 10.1016/j.ejcb.2017.01.005

[jex2109-bib-0033] Fekete, E. , Allain, T. , Amat, C. B. , Mihara, K. , Saifeddine, M. , Hollenberg, M. D. , Chadee, K. , & Buret, A. G. (2022). Giardia duodenalis cysteine proteases cleave proteinase‐activated receptor‐2 to regulate intestinal goblet cell mucin gene expression. International Journal of Parasitology, 52, 285–292. 35077730 10.1016/j.ijpara.2021.11.011

[jex2109-bib-0034] Fekete, E. , Allain, T. , Siddiq, A. , Sosnowski, O. , & Buret, A. G. (2020). Giardia spp. and the gut microbiota: Dangerous liaisons. Fronties in Microbiology, 11, 618106. 10.3389/fmicb.2020.618106 PMC7835142 33510729

[jex2109-bib-0035] Ferreira, B. , Lourenco, A. , & Sousa, M. D. C. (2022). Protozoa‐derived extracellular vesicles on intercellular communication with special emphasis on Giardia lamblia. Microorganisms, 10, 2422. 36557675 10.3390/microorganisms10122422 PMC9788250

[jex2109-bib-0036] Friedlander, M. R. , Mackowiak, S. D. , Li, N. , Chen, W. , & Rajewsky, N. (2012). miRDeep2 accurately identifies known and hundreds of novel microRNA genes in seven animal clades. Nucleic Acids Research, 40, 37–52. 21911355 10.1093/nar/gkr688 PMC3245920

[jex2109-bib-0037] Gavinho, B. , Sabatke, B. , Feijoli, V. , Rossi, I. V. , Da Silva, J. M. , Evans‐Osses, I. , Palmisano, G. , Lange, S. , & Ramirez, M. I. (2020). Peptidylarginine deiminase inhibition abolishes the production of large extracellular vesicles from giardia intestinalis, affecting host‐pathogen interactions by hindering adhesion to host cells. Frontiers in Cellular and Infection Microbiology, 10, 417. 33072615 10.3389/fcimb.2020.00417 PMC7539837

[jex2109-bib-0038] Gerbaba, T. K. , Gupta, P. , Rioux, K. , Hansen, D. , & Buret, A. G. (2015). Giardia duodenalis‐induced alterations of commensal bacteria kill Caenorhabditis elegans: A new model to study microbial‐microbial interactions in the gut. American Journal of Physiology‐Gastrointestinal and Liver Physiology, 308, G550–G561. 25573177 10.1152/ajpgi.00335.2014 PMC4360045

[jex2109-bib-0039] Halliez, M. C. , & Buret, A. G. (2013). Extra‐intestinal and long term consequences of Giardia duodenalis infections. World Journal of Gastroenterology, 19, 8974–8985. 24379622 10.3748/wjg.v19.i47.8974 PMC3870550

[jex2109-bib-0040] Hendricks, M. R. , Lane, S. , Melvin, J. A. , Ouyang, Y. , Stolz, D. B. , Williams, J. V. , Sadovsky, Y. , & Bomberger, J. M. (2021). Extracellular vesicles promote transkingdom nutrient transfer during viral‐bacterial co‐infection. Cell Report, 34, 108672. 10.1016/j.celrep.2020.108672 PMC7918795 33503419

[jex2109-bib-0041] Hiemstra, T. F. , Charles, P. D. , Gracia, T. , Hester, S. S. , Gatto, L. , Al‐Lamki, R. , Floto, R. A. , Su, Y. , Skepper, J. N. , Lilley, K. S. , & Karet Frankl, F. E (2014). Human urinary exosomes as innate immune effectors. Journal of American Society of Nephrology, 25, 2017–2027. 10.1681/ASN.2013101066 PMC4147985 24700864

[jex2109-bib-0042] Huang, D. W. , Sherman, B. T. , Tan, Q. , Collins, J. R. , Alvord, W. G. , Roayaei, J. , Stephens, R. , Baseler, M. W. , Lane, H. C. , & Lempicki, R. A. (2007a). The DAVID gene functional classification tool: A novel biological module‐centric algorithm to functionally analyze large gene lists. Genome Biology, 8, R183. 17784955 10.1186/gb-2007-8-9-r183 PMC2375021

[jex2109-bib-0043] Huang, D. W. , Sherman, B. T. , Tan, Q. , Kir, J. , Liu, D. , Bryant, D. , Guo, Y. , Stephens, R. , Baseler, M. W. , Lane, H. C. , & Lempicki, R. A. (2007b). DAVID Bioinformatics Resources: Expanded annotation database and novel algorithms to better extract biology from large gene lists. Nucleic Acids Research, 35, W169–W175. 17576678 10.1093/nar/gkm415 PMC1933169

[jex2109-bib-0044] Jung, M. , Schaefer, A. , Steiner, I. , Kempkensteffen, C. , Stephan, C. , Erbersdobler, A. , & Jung, K. (2010). Robust microRNA stability in degraded RNA preparations from human tissue and cell samples. Clinical Chemisty, 56, 998–1006. 10.1373/clinchem.2009.141580 20378769

[jex2109-bib-0045] Kalischuk, L. D. , Inglis, G. D. , & Buret, A. G. (2009). Campylobacter jejuni induces transcellular translocation of commensal bacteria via lipid rafts. Gut Pathogens, 1, 2. 19338680 10.1186/1757-4749-1-2 PMC2653720

[jex2109-bib-0046] Keller, R. , Pedroso, M. Z. , Ritchmann, R. , & Silva, R. M. (1998). Occurrence of virulence‐associated properties in Enterobacter cloacae. Infection and Immunity, 66, 645—649. 9453621 10.1128/iai.66.2.645-649.1998 PMC113501

[jex2109-bib-0047] Kery, M. B. , Feldman, M. , Livny, J. , & Tjaden, B. (2014). TargetRNA2: identifying targets of small regulatory RNAs in bacteria. Nucleic Acids Research, 42, W124–W129. 24753424 10.1093/nar/gku317 PMC4086111

[jex2109-bib-0048] Keselman, A. , Li, E. , Maloney, J. , & Singer, S. M. (2016). The microbiota contributes to CD8+ T cell activation and nutrient malabsorption following intestinal infection with Giardia duodenalis. Infection and Immunity, 84, 2853–2860. 27456829 10.1128/IAI.00348-16 PMC5038064

[jex2109-bib-0049] Kim, M. , You, B. H. , & Nam, J. W. (2015). Global estimation of the 3' untranslated region landscape using RNA sequencing. Methods, 83, 111–117. 25899044 10.1016/j.ymeth.2015.04.011

[jex2109-bib-0050] Koga, Y. , Yasunaga, M. , Moriya, Y. , Akasu, T. , Fujita, S. , Yamamoto, S. , & Matsumura, Y. (2011). Exosome can prevent RNase from degrading microRNA in feces. Journal of Gastrointestinal Oncology, 2, 215–222. 22811855 10.3978/j.issn.2078-6891.2011.015 PMC3397623

[jex2109-bib-0051] Kuhnert, P. , Nicolet, J. , & Frey, J. (1995). Rapid and accurate identification of Escherichia coli K‐12 strains. Applied Environmental Microbiology, 61, 4135–4139. 8526531 10.1128/aem.61.11.4135-4139.1995 PMC167724

[jex2109-bib-0052] Lambertz, U. , Oviedo Ovando, M. E. , Vasconcelos, E. J. , Unrau, P. J. , Myler, P. J. , & Reiner, N. E. (2015). Small RNAs derived from tRNAs and rRNAs are highly enriched in exosomes from both old and new world Leishmania providing evidence for conserved exosomal RNA Packaging. BMC Genomics, 16, 151. 25764986 10.1186/s12864-015-1260-7 PMC4352550

[jex2109-bib-0053] Law, C. W. , Alhamdoosh, M. , Su, S. , Dong, X. , Tian, L. , Smyth, G. K. , & Ritchie, M. E. (2016). RNA‐seq analysis is easy as 1‐2‐3 with limma, Glimma and edgeR. F1000Research, 5, 1408. 10.12688/f1000research.9005.1 PMC4937821 27441086

[jex2109-bib-0054] Lee, H. J. (2019). Microbe‐host communication by small RNAs in extracellular vesicles: Vehicles for transkingdom RNA transportation. International Journal of Molecular Science, 20, 1487. 10.3390/ijms20061487 PMC6472211 30934547

[jex2109-bib-0055] Liao, J. Y. , Guo, Y. H. , Zheng, L. L. , Li, Y. , Xu, W. L. , Zhang, Y. C. , Zhou, H. , Lun, Z. R. , Ayala, F. J. , & Qu, L. H. (2014). Both endo‐siRNAs and tRNA‐derived small RNAs are involved in the differentiation of primitive eukaryote Giardia lamblia. Proceedings of the National Academy of Sciences U S A, 111, 14159–14164. 10.1073/pnas.1414394111 PMC4191773 25225396

[jex2109-bib-0056] Liu, J. , Ma'ayeh, S. , Peirasmaki, D. , Lundstrom‐Stadelmann, B. , Hellman, L. , & Svard, S. G. (2018). Secreted Giardia intestinalis cysteine proteases disrupt intestinal epithelial cell junctional complexes and degrade chemokines. Virulence, 9, 879–894. 29726306 10.1080/21505594.2018.1451284 PMC5955458

[jex2109-bib-0057] Liu, S. , Da Cunha, A. P. , Rezende, R. M. , Cialic, R. , Wei, Z. , Bry, L. , Comstock, L. E. , Gandhi, R. , & Weiner, H. L. (2016). The Host Shapes the Gut Microbiota via Fecal MicroRNA. Cell Host & Microbe, 19, 32–43. 26764595 10.1016/j.chom.2015.12.005 PMC4847146

[jex2109-bib-0058] Marcilla, A. , Martin‐Jaular, L. , Trelis, M. , De Menezes‐Neto, A. , Osuna, A. , Bernal, D. , Fernandez‐Becerra, C. , Almeida, I. C. , & Del Portillo, H. A. (2014). Extracellular vesicles in parasitic diseases. Journal of Extracellular Vesicles, 3, 25040. 25536932 10.3402/jev.v3.25040 PMC4275648

[jex2109-bib-0059] Motta, J. P. , Allain, T. , Green‐Harrison, L. E. , Groves, R. A. , Feener, T. , Ramay, H. , Beck, P. L. , Lewis, I. A. , Wallace, J. L. , & Buret, A. G. (2018). Iron sequestration in microbiota biofilms as a novel strategy for treating inflammatory bowel disease. Inflammatory Bowel Disease, 24, 1493–1502. 10.1093/ibd/izy116 PMC5995063 29788224

[jex2109-bib-0060] Moyano, S. , Musso, J. , Feliziani, C. , Zamponi, N. , Frontera, L. S. , Ropolo, A. S. , Lanfredi‐Rangel, A. , Lalle, M. , & Touz, M. (2019). Exosome biogenesis in the protozoa parasite Giardia lamblia: A model of reduced interorganellar crosstalk. Cells, 8, 1600. 31835439 10.3390/cells8121600 PMC6953089

[jex2109-bib-0061] Natali, L. , Luna Pizarro, G. , Moyano, S. , De La Cruz‐Thea, B. , Musso, J. , Ropolo, A. S. , Eichner, N. , Meister, G. , Musri, M. M. , Feliziani, C. , & Touz, M. C. (2023). The exosome‐like vesicles of Giardia assemblages A, B, and E are involved in the delivering of distinct small RNA from parasite to parasite. International Journal of Molecular Science, 24, 9559. 10.3390/ijms24119559 PMC10253879 37298511

[jex2109-bib-0062] Nesvizhskii, A. I. , Keller, A. , Kolker, E. , & Aebersold, R. (2003). A statistical model for identifying proteins by tandem mass spectrometry. Analytical Chemistry, 75, 4646–4658. 14632076 10.1021/ac0341261

[jex2109-bib-0063] Nievas, Y. R. , Lizarraga, A. , Salas, N. , Coceres, V. M. , & De Miguel, N. (2020). Extracellular vesicles released by anaerobic protozoan parasites: Current situation. Cellular Microbiology, 22, e13257. 32858768 10.1111/cmi.13257

[jex2109-bib-0064] Ortega‐Pierres, G. , Arguello‐Garcia, R. , Laredo‐Cisneros, M. S. , Fonseca‐Linan, R. , Gomez‐Mondragon, M. , Inzunza‐Arroyo, R. , Flores‐Benitez, D. , Raya‐Sandino, A. , Chavez‐Munguia, B. , Ventura‐Gallegos, J. L. , Zentella‐Dehesa, A. , Bermudez‐Cruz, R. M. , & Gonzalez‐Mariscal, L. (2018). Giardipain‐1, a protease secreted by Giardia duodenalis trophozoites, causes junctional, barrier and apoptotic damage in epithelial cell monolayers. International Journal of Parasitology, 48, 621–639. 29571981 10.1016/j.ijpara.2018.01.006

[jex2109-bib-0065] Podolak, I. , Galanty, A. , & Sobolewska, D. (2010). Saponins as cytotoxic agents: A review. Phytochemistry Reviews, 9, 425–474. 20835386 10.1007/s11101-010-9183-z PMC2928447

[jex2109-bib-0066] Reti, K. L. , Tymensen, L. D. , Davis, S. P. , Amrein, M. W. , & Buret, A. G. (2015). Campylobacter jejuni increases flagellar expression and adhesion of noninvasive Escherichia coli: effects on enterocytic Toll‐like receptor 4 and CXCL‐8 expression. Infection and Immunity, 83, 4571–4581. 26371123 10.1128/IAI.00970-15 PMC4645401

[jex2109-bib-0067] Rodriguez, B. V. , & Kuehn, M. J. (2020). Staphylococcus aureus secretes immunomodulatory RNA and DNA via membrane vesicles. Scintific Reports, 10, 18293. 10.1038/s41598-020-75108-3 PMC7589478 33106559

[jex2109-bib-0068] Salomon, C. , Scholz‐Romero, K. , Sarker, S. , Sweeney, E. , Kobayashi, M. , Correa, P. , Longo, S. , Duncombe, G. , Mitchell, M. D. , Rice, G. E. , & Illanes, S. E. (2016). Gestational diabetes mellitus is associated with changes in the concentration and bioactivity of placenta‐derived exosomes in maternal circulation across gestation. Diabetes, 65, 598–609. 26718504 10.2337/db15-0966

[jex2109-bib-0069] Salomon, C. , Yee, S. , Scholz‐Romero, K. , Kobayashi, M. , Vaswani, K. , Kvaskoff, D. , Illanes, S. E. , Mitchell, M. D. , & Rice, G. E. (2014). Extravillous trophoblast cells‐derived exosomes promote vascular smooth muscle cell migration. Frontiers in Pharmacology, 5, 175. 25157233 10.3389/fphar.2014.00175 PMC4128075

[jex2109-bib-0070] Sanchez‐Torres, V. , Hu, H. , & Wood, T. K. (2011). GGDEF proteins YeaI, YedQ, and YfiN reduce early biofilm formation and swimming motility in Escherichia coli. Applied Microbiology Biotechnology, 90, 651–658. 21181144 10.1007/s00253-010-3074-5 PMC3158426

[jex2109-bib-0071] Sartor, R. B. , & Wu, G. D. (2017). Roles for intestinal bacteria, viruses, and fungi in pathogenesis of inflammatory bowel diseases and therapeutic approaches. Gastroenterology, 152, 327–339. e324. 27769810 10.1053/j.gastro.2016.10.012 PMC5511756

[jex2109-bib-0072] Singer, S. M. , & Nash, T. E. (2000). The role of normal flora in Giardia lamblia infections in mice. Journal of Infectious Disease, 181, 1510–1512. 10.1086/315409 10751141

[jex2109-bib-0073] Statello, L. , Maugeri, M. , Garre, E. , Nawaz, M. , Wahlgren, J. , Papadimitriou, A. , Lundqvist, C. , Lindfors, L. , Collen, A. , Sunnerhagen, P. , Ragusa, M. , Purrello, M. , Di Pietro, C. , Tigue, N. , & Valadi, H. (2018). Identification of RNA‐binding proteins in exosomes capable of interacting with different types of RNA: RBP‐facilitated transport of RNAs into exosomes. PLoS One, 13, e0195969. 29689087 10.1371/journal.pone.0195969 PMC5918169

[jex2109-bib-0074] Szklarczyk, D. , Gable, A. L. , Lyon, D. , Junge, A. , Wyder, S. , Huerta‐Cepas, J. , Simonovic, M. , Doncheva, N. T. , Morris, J. H. , Bork, P. , Jensen, L. J. , & Mering, C. V. (2019). STRING v11: Protein‐protein association networks with increased coverage, supporting functional discovery in genome‐wide experimental datasets. Nucleic Acids Research, 47, D607–D613. 30476243 10.1093/nar/gky1131 PMC6323986

[jex2109-bib-0075] Teoh, D. A. , Kamieniecki, D. , Pang, G. , & Buret, A. G. (2000). Giardia lamblia rearranges F‐actin and alpha‐actinin in human colonic and duodenal monolayers and reduces transepithelial electrical resistance. Journal of Parasitology, 86, 800–806. 10958459 10.1645/0022-3395(2000)086[0800:GLRFAA]2.0.CO;2

[jex2109-bib-0076] Wu, Z. , Wang, L. , Li, J. , Wang, L. , Wu, Z. , & Sun, X. (2018). Extracellular vesicle‐mediated communication within host‐parasite interactions. Frontiers in Immunology, 9, 3066. 30697211 10.3389/fimmu.2018.03066 PMC6340962

[jex2109-bib-0077] Yu, L. C. , Huang, C. Y. , Kuo, W. T. , Sayer, H. , Turner, J. R. , & Buret, A. G. (2008). SGLT‐1‐mediated glucose uptake protects human intestinal epithelial cells against Giardia duodenalis‐induced apoptosis. International Journal of Parasitology, 38, 923–934. 18281046 10.1016/j.ijpara.2007.12.004 PMC2693066

[jex2109-bib-0078] Yu, S. , Zhao, Z. , Xu, X. , Li, M. , & Li, P. (2019). Characterization of three different types of extracellular vesicles and their impact on bacterial growth. Food Chemistry, 272, 372–378. 30309557 10.1016/j.foodchem.2018.08.059

[jex2109-bib-0079] Zaborowski, M. P. , Balaj, L. , Breakefield, X. O. , & Lai, C. P. (2015). Extracellular vesicles: Composition, biological relevance, and methods of study. Bioscience, 65, 783—797. 26955082 10.1093/biosci/biv084 PMC4776721

[jex2109-bib-0080] Zhang, F. , Li, B. , Dong, H. , Chen, M. , Yao, S. , Li, J. , Zhang, H. , Liu, X. , Wang, H. , Song, N. , Zhang, K. , Du, N. , Xu, S. , & Gu, L. (2020). YdiV regulates Escherichia coli ferric uptake by manipulating the DNA‐binding ability of Fur in a SlyD‐dependent manner. Nucleic Acids Research, 48, 9571—9588. 32813023 10.1093/nar/gkaa696 PMC7515728

[jex2109-bib-0081] Zhao, P. , Cao, L. , Wang, X. , Dong, J. , Zhang, N. , Li, X. , Li, J. , Zhang, X. , & Gong, P. (2021). Extracellular vesicles secreted by Giardia duodenalis regulate host cell innate immunity via TLR2 and NLRP3 inflammasome signaling pathways. PloS Neglected Tropical Disease, 15, e0009304. 10.1371/journal.pntd.0009304 PMC8046354 33798196

