# Supplementary figures and images for: A thermo‐resistant and RNase‐sensitive cargo from Giardia duodenalis extracellular vesicles modifies the behaviour of enterobacteria
Source: J Extracell Biol. 2023 Aug 30;2(9):e109. doi: 10.1002/jex2.109 (PMC11080815; doi:10.1002/jex2.109)

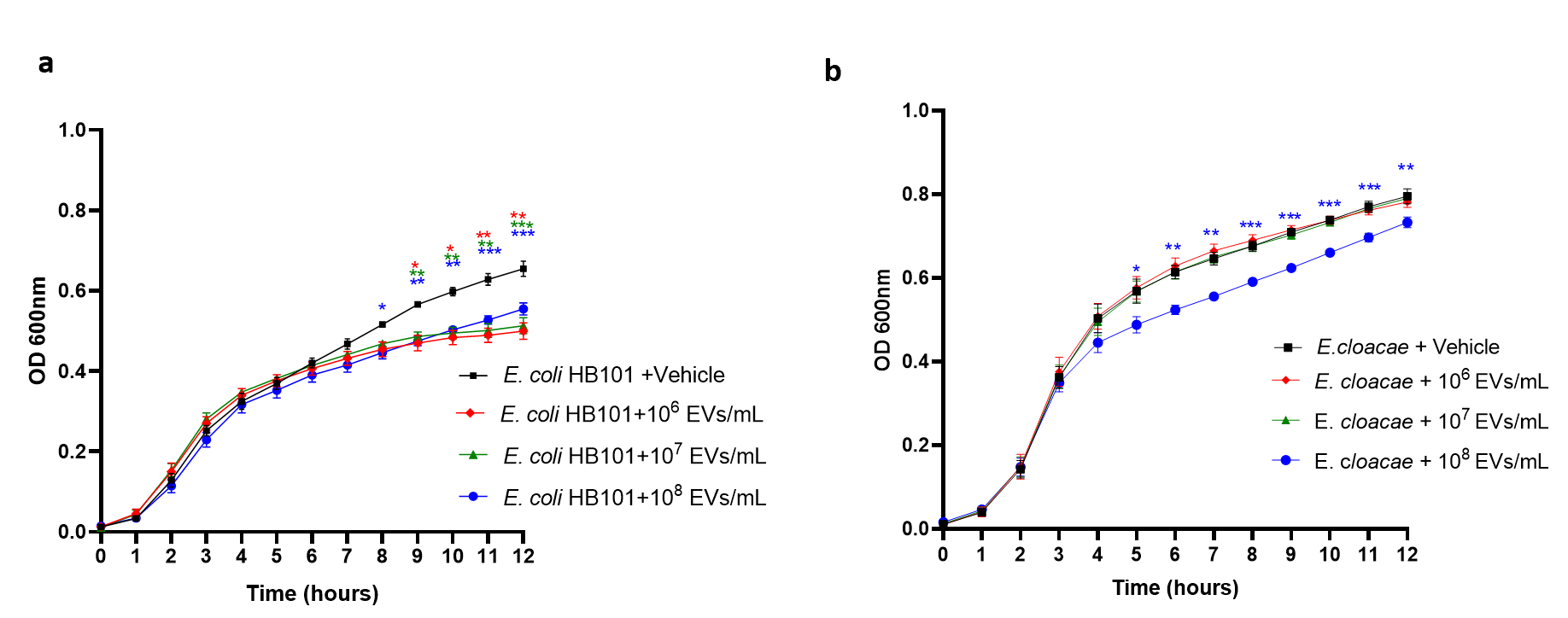

Supplement: Supplementary file 8 — Supplementary Information [file JEX2-2-e109-s007.tif]

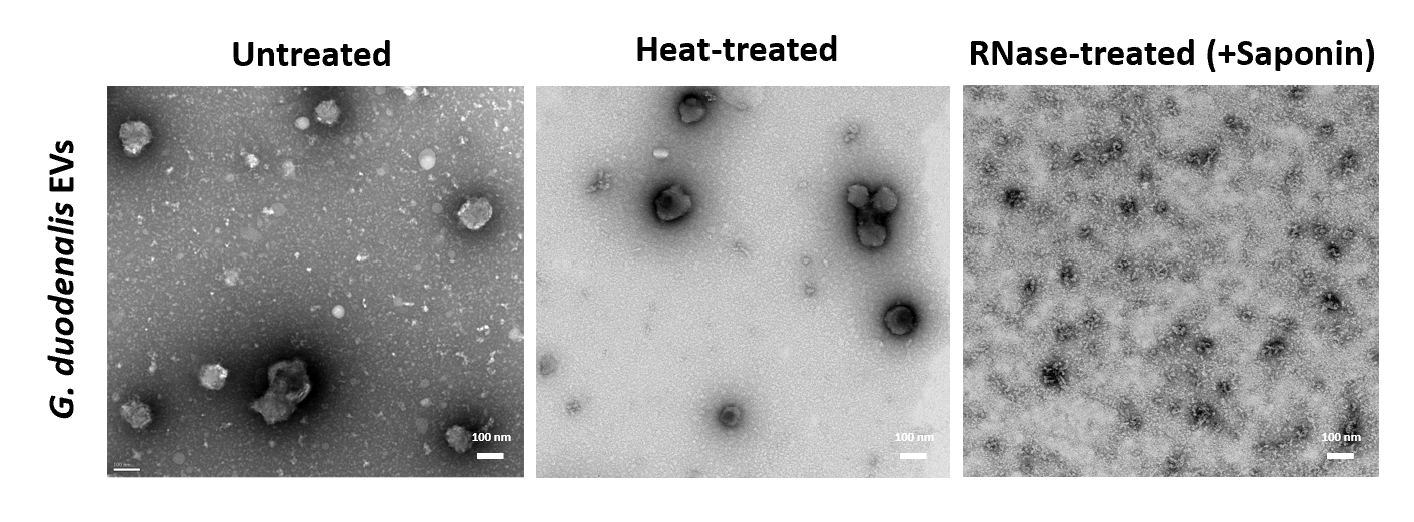

Supplement: Supplementary file 9 — Supplementary Information [file JEX2-2-e109-s004.tif]
