# Supplementary material for: A thermo‐resistant and RNase‐sensitive cargo from Giardia duodenalis extracellular vesicles modifies the behaviour of enterobacteria
Source: J Extracell Biol. 2023 Aug 30;2(9):e109. doi: 10.1002/jex2.109 (PMC11080815; doi:10.1002/jex2.109)
Supplement: Supplementary file 2 — Supplementary Information [file JEX2-2-e109-s003.pdf]

miRDeep2

Parameters used

miRDeep2 version	2.0.1.3
Program call	/home/users/allstaff/balan.b/.conda/envs/miRNA_New/bin/miRDeep2.pl ./LCS8251_AT_Control_RawData_trim_collapsed.fa ./GiardiaDB-54_GintestinalisAssemblageAWB_Genome_Thibult_nowhitespace.fasta ./Control_vs_genome.arf Mature.fa none Hairpin.fa
Reads	./LCS8251_AT_Control_RawData_trim_collapsed.fa
Genome	./GiardiaDB-54_GintestinalisAssemblageAWB_Genome_Thibult_nowhitespace.fasta
Mappings	./Control_vs_genome.arf
Reference mature miRNAs	Mature.fa
Other mature miRNAs	none

Survey of miRDeep2 performance for score cut-offs -10 to 10

	novel miRNAs			known miRBase miRNAs				
miRDeep2 score	predicted by miRDeep2	estimated false positives	estimated true positives	in species	in data	detected by miRDeep2	estimated signal-to-noise	excision gearing
10	3	1 ± 1	2 ± 1 (69 ± 32%)	4574	0	0 (0%)	3.1	1
9	3	1 ± 1	2 ± 1 (67 ± 32%)	4574	0	0 (0%)	2.9	1
8	3	1 ± 1	2 ± 1 (67 ± 32%)	4574	0	0 (0%)	2.9	1
7	3	1 ± 1	2 ± 1 (66 ± 33%)	4574	0	0 (0%)	2.8	1
6	3	1 ± 1	2 ± 1 (66 ± 33%)	4574	0	0 (0%)	2.8	1
5	3	1 ± 1	2 ± 1 (64 ± 33%)	4574	0	0 (0%)	2.7	1
4	3	1 ± 1	2 ± 1 (61 ± 35%)	4574	0	0 (0%)	2.4	1
3	3	1 ± 1	2 ± 1 (59 ± 35%)	4574	0	0 (0%)	2.3	1
2	9	2 ± 1	7 ± 1 (79 ± 16%)	4574	0	0 (0%)	4.8	1
1	9	4 ± 2	5 ± 2 (58 ± 21%)	4574	0	0 (0%)	2.4	1
0	9	13 ± 4	0 ± 1 (2 ± 7%)	4574	0	0 (0%)	0.7	1
-1	10	20 ± 4	0 ± 0 (0 ± 1%)	4574	0	0 (0%)	0.5	1
-2	18	26 ± 5	0 ± 0 (0 ± 2%)	4574	0	0 (0%)	0.7	1
-3	48	39 ± 6	10 ± 6 (20 ± 11%)	4574	0	0 (0%)	1.2	1
-4	125	107 ± 11	18 ± 10 (14 ± 8%)	4574	0	0 (0%)	1.2	1
-5	165	557 ± 19	0 ± 0 (0 ± 0%)	4574	0	0 (0%)	0.3	1
-6	194	1025 ± 24	0 ± 0 (0 ± 0%)	4574	0	0 (0%)	0.2	1
-7	448	1325 ± 25	0 ± 0 (0 ± 0%)	4574	0	0 (0%)	0.3	1
-8	963	1540 ± 27	0 ± 0 (0 ± 0%)	4574	0	0 (0%)	0.6	1
-9	1293	1669 ± 29	0 ± 0 (0 ± 0%)	4574	0	0 (0%)	0.8	1
-10	1512	1742 ± 30	0 ± 0 (0 ± 0%)	4574	0	0 (0%)	0.9	1

novel miRNAs predicted by miRDeep2

provisional id	miRDeep2 score	estimated probability that the miRNA candidate is a true positive	rfam alert	total read count	mature read count	loop read count	star read count	significant randfold p-value	miRBase miRNA	example miRBase miRNA with the same seed	UCSC browser	NCBI blastn	consensus mature sequence	consensus star sequence	consensus precursor sequence	precursor coordinate
CH991838_22062	1.5e+3	0.69 ± 0.32		3101	3096	0	5	no				blast	aggggggacccccgacacggc	cggaggggggcggcccccgugc	aggggggacccccgacacggcaugggcacggaggggggcggcccccgugc	CH991838:1061..1110:+
GLCHR01_1162	1.5e+3	0.69 ± 0.32		3101	3096	0	5	no				blast	aggggggacccccgacacggc	cggaggggggcggcccccgugc	aggggggacccccgacacggcaugggcacggaggggggcggcccccgugc	GLCHR01:1469000..1469049:+
AACB02000118_21135	1.5e+3	0.69 ± 0.32		3101	3096	0	5	no				blast	aggggggacccccgacacggc	cggaggggggcggcccccgugc	aggggggacccccgacacggcaugggcacggaggggggcggcccccgugc	AACB02000118:654..703:-
GLCHR04_12081	2.7	0.79 ± 0.16		12	12	0	0	yes				blast	ucggggaggacggggaaguggacc	uucaccagcuucagcggugggcuccgacc	ucggggaggacggggaaguggaccgccgggaugaggggcggcggugggcuucucaaagcugggguucaccagcuucagcggugggcuccgacc	GLCHR04:2202817..2202908:-
GLCHR04_11919	2.6	0.79 ± 0.16		12	12	0	0	yes				blast	ucggggaggacggggaaguggacc	uucaccagcuucagcggugggcuccgacc	ucggggaggacggggaaguggaccgccgggaugaggggcggcggugggcuuuucaaagcugggguucaccagcuucagcggugggcuccgacc	GLCHR04:2073560..2073651:-
GLCHR05_19494	2.4	0.79 ± 0.16		77	77	0	0	yes				blast	aggaggaggaugaggaaggu	cugucuuucuagcugcuug	cugucuuucuagcugcuugucgcgcuucagcucgcagcaguggagacugauugugacgaggaggaggaugaggaaggu	GLCHR05:3997811..3997889:-
GLCHR04_8723	2.3	0.79 ± 0.16		77	77	0	0	yes				blast	aggaggaggaugaggaaggu	cugucuuucuagcugcuug	cugucuuucuagcugcuugucgcgcuucagcucgcagcaguggaggcugauugugacgaggaggaggaugaggaaggu	GLCHR04:889134..889212:+
GLCHR05_15271	2.2	0.79 ± 0.16		77	77	0	0	yes				blast	gagaaagaaaaggaggacuucgcc	cgaguguccucugcgucgc	cgaguguccucugcgucgcaagccuuguuagggcuggcgagaaagaaaaggaggacuucgcc	GLCHR05:3489956..3490018:+
GLCHR04_8448	2.0	0.79 ± 0.16		14	14	0	0	yes				blast	ggcgagagaucgaggcaggg	cugccucaucugugcaga	ggcgagagaucgaggcagggaagcgccagcucucuacugccucaucugugcaga	GLCHR04:536112..536166:+

mature miRBase miRNAs detected by miRDeep2

tag id	miRDeep2 score	estimated probability that the miRNA is a true positive	rfam alert	predicted mature seq. in accordance with miRBase mature seq.	total read count	mature read count	loop read count	star read count	significant randfold p-value	mature miRBase miRNA	example miRBase miRNA with the same seed	UCSC browser	NCBI blastn	consensus mature sequence	consensus star sequence	consensus precursor sequence	precursor coordinate

mature miRBase miRNAs not detected by miRDeep2

miRBase precursor id	total read count	mature read count(s)	star read count	remaining reads	UCSC browser	NCBI blastn	miRBase mature sequence(s)	miRBase star sequence(s)	miRBase precursor sequence
ppc-mir-9	6608	6608	0	0	-	blast	ucuuugguuaucuagcuguauga<br>ucuuugguuaucuagcuguauga<br>ucuuugguuaucuagcuguaug<br>ucuuugguuaucuagcuguauga<br>ucuuugguuaucuagcuguauga<br>ucuuugguuaucuagcuguauga<br>ucuuugguuaucuagcuguauga<br>cuuugguuaucuagcuguauga	-	uuccuucaaugggacagucauuaucgucucuuugguuaucuagcuguaugauuugaaauguaaaucauaaagcuggaaaacugaaggaacgaugugucucaugga
sme-mir-124c-2	9282	9282	0	0	-	blast	uaaggcacgcggugaaugcca<br>uaaggcacgcggugaaugcca<br>gcauuaacccuguugucuuagau<br>uaaggcacgcggugaaugcc<br>uaaggcacgcggugaaugc<br>uaaggcacgcggugaaugcca<br>uaaggcacgcggugaaugcc<br>uaaggcacgcggugaaugcca<br>uaaggcacgcggugaaugcca<br>uaaggcacgcggugaaugcca<br>uaaggcacgcggugaaugcca<br>uaaggcacgcggugaaugcca<br>uaaggcacgcggugaaugcca<br>uaaggcacgcggugaaugcca<br>uaaggcacgcggugaaugcca	-	ccauuauauucaugaagcauuaacccuguugucuuagauaguuuauuacaaaacauuuaaggcacgcggugaaugccaugugaaaugg
prd-let-7	6710	6710	0	0	-	blast	ugagguaguagguuguauaguuu<br>ugagguaguagguuguauaguu<br>ugagguaguagguuguauaguu<br>ugagguaguagguuguauaguu<br>ugagguaguagguuguauaguu<br>ugagguaguagguuguauaguu<br>ugagguaguagguuguauaguu<br>ugagguaguagguuguauagu<br>ugagguaguagguuguauaguu<br>ugagguaguagguuguauaguu<br>ugagguaguagguuguauaguu<br>acugucaaucuacuaacuuaac	-	ugagguaguagguuguauaguuuuugauuguauaaaaacugucaaucuacuaacuuaac
prd-mir-124	8670	8670	0	0	-	blast	uaaggcacgcggugaaugccaa<br>uaaggcacgcggugaaugcca<br>uaaggcacgcggugaaugcca<br>uaaggcacgcggugaaugcca<br>uaaggcacgcggugaaugcca<br>uaaggcacgcggugaaugcca<br>uaaggcacgcggugaaugcca<br>uaaggcacgcggugaaugcca<br>uaaggcacgcggugaaugccaau<br>uaaggcacgcggugaaugccaa<br>uaaggcacgcggugaaugc<br>uaaggcacgcggugaaugcc<br>uaaggcacgcggugaaugcca<br>uaaggcacgcggugaaugcca<br>uaaggcacgcggugaaugcca<br>uaaggcacgcggugaaugcca<br>uaaggcacgcggugaaugcc<br>ggguuuucacggugacuucagu	-	ggguuuucacggugacuucagugagaauuaaaucaacuaaggcacgcggugaaugccaau
cte-mir-125	466	466	0	0	-	blast	ucccugagacccuaacuuguga	-	uaaccacucggcaucucgcacucccugagacccuaacuugugaagcuuucuaaaaagaucgcgaguuagaaucuugggcucugcgaggagcucgauugccg
prd-mir-100	1880	1880	0	0	-	blast	aacccguagauccgaacuugug<br>aacccguagauccgaacuugug<br>aacccguagauccgaacuuguguu<br>cacaucuucgguaugggcgg<br>aacccguagauccgaacuugug<br>aacccguagauccgaacuuguguu	-	aacccguagauccgaacuuguguucugucaacaagaaacacaucuucgguaugggcgg
sme-mir-124a	448	448	0	0	-	blast	uaaggcacgcggugaaugcuu<br>uaaggcacgcggugaaugc<br>ugcuuuuaacgcggagcuuuagu	-	uucaaucagaugugcuuuuaacgcggagcuuuaguaauuuaacauguauacuaaggcacgcggugaaugcuuacuugauugag
lgi-mir-7	222	222	0	0	-	blast	uggaagacuagugauuuuguugu	-	auaaccuaucuacccuggucuggaagacuagugauuuuguuguuuuaauuucaguauuaacaauaaaucacauucuucccagauugguuguaucuuaaca
sme-mir-124b	442	442	0	0	-	blast	uaaggcacgcggugaaugc<br>uaaggcacgcggugaaugcuga<br>ugcauuuacaacgugucuuuagu	-	guauccaugaaaauuugcauuuacaacgugucuuuaguuucaugugacacuaaggcacgcggugaaugcugaauuaaaugguuua
str-mir-124	193	193	0	0	-	blast	uaaggcacgcggugaaugc<br>cgcuuucauccgugacuuuaga	-	cgcuuucauccgugacuuuagaagagucuuauaucacuaaggcacgcggugaaugc
sma-mir-124a	111	111	0	0	-	blast	ccauuuuccgcgauugccuugauga<br>uaaggcacgcggugaauguca<br>uuaaggcacgcggugaauguca<br>uaaggcacgcggugaauguca	-	uuuguaugccauuuuccgcgauugccuugaugaguuaauaaauauuuaucauaacaaaaauauuaaggcacgcggugaaugucauccacggagc
mle-mir-29a	105	105	0	0	-	blast	ccugaucucuccuggugcauaga<br>uagcaccauuugaaaucaguuu<br>uagcaccauuugaaaucaguuu<br>uagcaccauuugaaaucaguuu	-	ccugaucucuccuggugcauagauuugauccugcgucuagcaccauuugaaaucaguuu
bma-mir-7	35	35	0	0	-	blast	uggaagacuugugauuuuguugu	-	aaaguuaaagcugaccagguggaagacuugugauuuuguuguuuauacauuuucagcaacagcaaaucgagcucuccacccggaggcau
mle-mir-8	42	42	0	0	-	blast	uaauacugucagguaaagauguc<br>uaauacugucagguaaagauguc<br>uaauacugucagguaaagauguc<br>ggucuuaccuagcagcauugga	-	ggucuuaccuagcagcauuggauuguucugcucuaauacugucagguaaagauguc
cel-mir-8210	13	13	0	0	-	blast	cgacgaaaaaaagaaagaaagaaa<br>ugccuucuuuccuugugucgccgac	-	ugccuucuuuccuugugucgccgacaaaucgccacaauccgucgacgaaaaaaagaaagaaagaaa
gsa-mir-124a	36	36	0	0	-	blast	uaaggcacgcggugaauacc<br>uaaggcacgcggugaauacc<br>gguauuuuccacgaagucuuagg<br>uaaggcacgcggugaauacc	-	gguauuuuccacgaagucuuaggaaguugaaaaauaaaaauauuuaacuuaaggcacgcggugaauacc
mle-mir-153	36	36	0	0	-	blast	uugcauagucacaaaagugauc<br>uugcauagucacaaaagugauc<br>ccgcuuuugugguuuagcaauu<br>uugcauagucacaaaagugauc	-	ccgcuuuugugguuuagcaauuguacucugggcaaauugcauagucacaaaagugauc
mle-mir-92b	12	12	0	0	-	blast	uauugcacuuuucccggccugu<br>gggucgagauucuugcaaaag	-	gggucgagauucuugcaaaaguguuacaucgacucuuauugcacuuuucccggccugu
mle-mir-29b	24	24	0	0	-	blast	uagcaccauuugaaaucagug<br>cugauuuccagugguggauaga<br>uagcaccauuugaaaucagug	-	cugauuuccagugguggauagaugucccuuagggcucgagucuugcuagcaccauuugaaaucagug
gsa-mir-124b	12	12	0	0	-	blast	guguuuaccgugggcuugau<br>uaaggcacgcggugaauauc	-	guguuuaccgugggcuugauuugguuguuauuuuauuaaggcacgcggugaauauc
mco-mir-9	16	16	0	0	-	blast	ucuuugguuaucuagcugugug<br>ucuuugguuaucuagcugugug<br>caacgcuagauuuccaaacaa	-	ucuuugguuaucuagcugugugacagguagcuucaucuggcuucuuuuauuaucacaacgcuagauuuccaaacaaa
mle-bantam	8	8	0	0	-	blast	cugguuuucacugugauuuacaga<br>ugagaucauugugaaaacugauu	-	cugguuuucacugugauuuacagauauuuccaacaauucugagaucauugugaaaacugauu
mle-mir-184	15	15	0	0	-	blast	uggacggagaacugauaagggc<br>uggacggagaacugauaagggc<br>ccuuaucacuuuucccuccugu<br>uggacggagaacugauaagggc	-	ccuuaucacuuuucccuccuguuuuugauuugaauacuggacggagaacugauaagggc
hco-mir-5991	5	5	0	0	-	blast	ugagguaguagguggugucga	-	ucuucauaacauuuuauuuuuagcgugaucgagcuaggcuucuauuugccuuugauuucgcuucgucauugagguaguagguggugucgauugaugccau
bma-mir-153	5	5	0	0	-	blast	uugcauagucacaaaagugaug	-	uuccgcucgucgaucauuuuugguggauuagcaauuuuguucucgaauaauugcauagucacaaaagugauggacgugaagcgucguuugu
prd-mir-1	40	40	0	0	-	blast	uggaauguaaagaaguaugua<br>uggaauguaaagaaguauguag<br>uggaauguaaagaaguaugua<br>uggaauguaaagaaguaugua<br>uggaauguaaagaaguauguag<br>uggaauguaaagaaguauguag<br>uggaauguaaagaaguaugu<br>uggaauguaaagaaguaugua<br>uggaauguaaagaaguaugua<br>acgugcuucuuugcaagaccaua<br>uggaauguaaagaaguaugu	-	acgugcuucuuugcaagaccauagugaauacuguuuauggaauguaaagaaguauguag
mle-mir-219	8	8	0	0	-	blast	agaacuguggguggacaucagu<br>ugauuguccaaacgcaauucu<br>ugauuguccaaacgcaauucug	-	ugauuguccaaacgcaauucuuguuacaucucaaccaagaacugugggguggacaucagu
ppc-mir-235	16	16	0	0	-	blast	uauugcacucgucccggccuga<br>aggccgcggucggaugccauguu<br>uauugcacucgucccggccug<br>uauugcacucgucccggccuga<br>uauugcacucgucccggccu	-	aggccgcggucggaugccauguucgcacagucgaagcguaauauugcacucgucccggccuga
prd-mir-235	6	6	0	0	-	blast	uauugcacucgucccggccu<br>uauugcacucgucccggccug<br>aggaaggggcguuuggaaugu	-	aggaaggggcguuuggaauguuucuuuucuguuguaauauugcacucgucccggccug
sme-mir-7d	3	3	0	0	-	blast	caacauuauuuucuguuuuccaa<br>uggaagacuaguaauuuuguugu	-	aaaaacuauaaaacaaucuuggaagacuaguaauuuuguuguaugcgaaauuuuaucagacauucaguuugagaggauuuacaacauuauuuucuguuuuccaagaacuuauagucaaucaacg
mle-mir-1	18	18	0	0	-	blast	uggaauguaaagaaguaugua<br>uggaauguaaagaaguaugua<br>acauacuuccuugcuaucccaua<br>uggaauguaaagaaguaugua<br>uggaauguaaagaaguaugua<br>uggaauguaaagaaguaugua<br>uggaauguaaagaaguaugu<br>uggaauguaaagaaguauguau<br>uggaauguaaagaaguaugua<br>uggaauguaaagaaguauguau	-	acauacuuccuugcuaucccauauuuauggcaaccuauggaauguaaagaaguauguau
mle-mir-317	2	2	0	0	-	blast	ugaacacagcugguggauccuau<br>agggugccauccugugcucaca	-	agggugccauccugugcucacauguugauaugaacaaagugugugugaacacagcugguggauccuau
bma-mir-81	4	4	0	0	-	blast	ugagaucauugugaaagcuau<br>ugagaucauugugaaagcuauu	-	gguggggcaguaggccuucaccaugaucuacagaaugcuaucauuuugagucugagaucauugugaaagcuauuuggccuucuaccgucuc
cte-mir-317	2	2	0	0	-	blast	ugaacacagcugguggauucucuuu	-	cggaaggagaagcgucgagcgcaggcaagagaucacugcggugcucacauguagccacugugaacacagcugguggauucucuuuucuucgcucugaccug
mle-mir-375	4	4	0	0	-	blast	uuuguucguucggcucgcguua<br>acacgagcaguucguaacaaggc<br>uuuguucguucggcucgcguua	-	acacgagcaguucguaacaaggccucccauuuagguguuuuguucguucggcucgcguua
lgi-mir-133	4	4	0	0	-	blast	uugguccccuucaaccagcugu<br>uugguccccuucaaccagcugu<br>agcugguugaaaugggccaaau	-	ugucuguagcugguugaaaugggccaaauuagaaaaggucaagcauuuggucccccuucaaccagcuguaguug
egr-mir-10271	2	2	0	0	-	blast	ggggguaagccaguccuauuuc<br>cgugggggcuugugguacuucug	-	cgugggggcuugugguacuucugggugcggggugcagugcgugugugguugcgcaggguaagccaguccuauuuc
cte-bantam	2	2	0	0	-	blast	ugagaucauugugaaaacuauuc	-	ucagagcaagacugcgagacugguuuucucuuuggcucuccagauuuuauuggaauucugagaucauugugaaaacuauucucgcugacauuacgccag
lgi-mir-317	2	2	0	0	-	blast	ugaacacagcugguggauucuucu	-	aagagaagauaacucuguugguggaguaccaguugguguuuaacaguuuugaaacauuugugaacacagcugguggauucuucuauaagcagaguauuuca
sme-mir-71c	3	3	0	0	-	blast	ugaaagacauggguaguga<br>ugaaagacauggguagugagau<br>ucucauuauucaaguuuuucuua<br>ugaaagacauggguaguga	-	guaguuuucuugaaagacauggguagugagauuauuuaaauuaaauauuuaaaaucucauuauucaaguuuuucuuauaagc
mle-mir-87	5	5	0	0	-	blast	gugagcaaaguuucaggugu<br>gugagcaaaguuucaggugu<br>gugagcaaaguuucagguguuu<br>gugagcaaaguuucaggugu<br>gugccugaaaccuuggcucaggccu<br>gugagcaaaguuucaggugu	-	gugccugaaaccuuggcucaggccuggcuauacgacaaggugagcaaaguuucagguguuu
str-mir-34a	7	7	0	0	-	blast	uggcagugugguuagcugguugu<br>uggcagugugguuagcugguug<br>uggcagugugguuagcugguug<br>acagcucacucaacugccaag<br>uggcagugugguuagcugguugu<br>uggcagugugguuagcugguugu<br>uggcagugugguuagcugguugu<br>uggcagugugguuagcugguug	-	uggcagugugguuagcugguugugauuuuuuagacgcucaacagcucacucaacugccaag
str-mir-72	12	12	0	0	-	blast	aguuaugcuauauguugccaga<br>aggcaagauguuggcauagcuga<br>aggcaagauguuggcauagcug<br>aggcaagauguuggcauagcug<br>aggcaagauguuggcauagcuga<br>aggcaagauguuggcauagcuga<br>aggcaagauguuggcauagcu<br>aggcaagauguuggcauagcuga<br>aggcaagauguuggcauagcu<br>aggcaagauguuggcauagcug<br>aggcaagauguuggcauagcuga<br>aggcaagauguuggcauagcuga<br>aggcaagauguuggcauagcuga	-	aggcaagauguuggcauagcugauagucgaaaauuauucaguuaugcuauauguugccaga
str-mir-71	9	9	0	0	-	blast	ugaaagacauggguagugagacg<br>ugaaagacauggguagugagac<br>uaucacuauucuguuuuucucc<br>ugaaagacauggguaguga<br>ugaaagacauggguagugagacg<br>ugaaagacauggguaguga<br>ugaaagacauggguagugagacg<br>ugaaagacauggguagugagac<br>ugaaagacauggguagugagacg<br>ugaaagacauggguagugagac	-	ugaaagacauggguagugagacgaugaguuugaauuccguaucacuauucuguuuuucucc
mle-mir-71	5	5	0	0	-	blast	ugaaagacauggguagugagaug<br>ugaaagacauggguaguga<br>ccuuacuacucugucuuuugca<br>ugaaagacauggguagugagau<br>ugaaagacauggguagugagaug<br>ugaaagacauggguaguga	-	ugaaagacauggguagugagauguuugugugacucaccuuacuacucugucuuuugca
mle-mir-279	3	3	0	0	-	blast	ugacuagauccacacucaucca<br>ugacuagauccacacucaucc<br>ugacuagauccacacucaucca<br>ggugauuguuggucuggugcaug	-	ggugauuguuggucuggugcauguuguuauuaucgggucaugacuagauccacacucaucca
prd-mir-87	12	12	0	0	-	blast	gugagcaaaguuucaggugugc<br>gugagcaaaguuucaggugugc<br>gugagcaaaguuucaggugu<br>gugagcaaaguuucaggugugc<br>gugagcaaaguuucaggugugc<br>ggccugggacuuggacucaaccu<br>gugagcaaaguuucaggugu<br>gugagcaaaguuucaggugugc<br>gugagcaaaguuucaggugugc<br>gugagcaaaguuucaggugu<br>gugagcaaaguuucaggugugc<br>gugagcaaaguuucaggugugc<br>gugagcaaaguuucaggugugc	-	ggccugggacuuggacucaaccugcguaggcucgaaguucgaggugagcaaaguuucaggugugc
lgi-mir-10	1	1	0	0	-	blast	uacccuguagauccgaauuugu	-	gaauauugugccuuuacguuuacccuguagauccgaauuuguguaugaaacaaucuacaaauucguuuuugcgggugauugugucgagguuuucaaaauca
lgi-mir-87	5	5	0	0	-	blast	gugagcaaaguuucaggugu<br>gugagcaaaguuucaggugu<br>gugagcaaaguuucagguguau<br>gugagcaaaguuucaggugu<br>gugagcaaaguuucagguguau	-	uuauuaacuugagguuugucugaggugccugaaauuuuagcucaaaccuaauaauagaaggugagcaaaguuucagguguaucagcugaacuuauauucu
str-mir-34b	1	1	0	0	-	blast	acagcuuaccaaauugccauc<br>uggcaguguaguuagcugguug	-	uggcaguguaguuagcugguugauguuuuaaaaaucaacagcuuaccaaauugccauc
fhe-mir-87	4	4	0	0	-	blast	gugagcaaaguuucaggugu<br>gugagcaaaguuucaggugu<br>ggugagcaaaguuucagguguga<br>gugagcaaaguuucaggugu	-	acaacugauaccucuugcacauguauggaauggugagcaaaguuucagguguga
sme-mir-31b-3	1	1	0	0	-	blast	aggcaagaugcuggcauagcuga<br>agcuauucaggaucuuuccau	-	caaggcaagaugcuggcauagcugauaaacuauuuauuaccagcuauucaggaucuuuccauga
lgi-mir-33	2	2	0	0	-	blast	gugcauuguaguugcauugca<br>gcaaugcaucugcagugcaaaca<br>gugcauuguaguugcauugcau	-	cgaacuaagcaguuguaugggugcauuguaguugcauugcauuuguuuauaaaauuaugcaaugcaucugcagugcaaacauuacgauugaaacuaucaccua
sme-mir-124e	1	1	0	0	-	blast	gccauucucaguuggagucuu<br>uaaggcacgcggugaaugcca	-	uucaauaaauuauucuacugccauucucaguuggagucuugauaugaucuauuaaggcacgcgugaaugccaaggaauuuuauuucucuucu
fhe-mir-9	1	1	0	0	-	blast	ucuuugguuaucaagcaguaug	-	ucuuugguuaucaagcaguaugauugucacacuugaaacggccagugacacaacaacacgcgcacuuugugcucgcgugauuguuucugugucgaugacacucggcucgucuaacaucauaaagcuaggugaccaaagu
hpo-mir-1	14	14	0	0	-	blast	uggaauguaaagaaguaugua<br>uggaauguaaagaaguaugua<br>cgugcuucuuugcaugcccaua<br>uggaauguaaagaaguaugua<br>uggaauguaaagaaguaugu<br>uggaauguaaagaaguaugua<br>uggaauguaaagaaguaugua<br>uggaauguaaagaaguaugu	-	cgugcuucuuugcaugcccauacuguauuuugauauggaauguaaagaaguaugua
emu-mir-9	16	16	0	0	-	blast	ucuuugguuaucuagcugugug<br>caaggcuagauuuccaaacaaa<br>caaggcuagauuuccaaacaa<br>ucuuugguuaucuagcugugug	-	uaguugccauucuuugguuaucuagcugugugagguuuagcuucagucagcucuuauuuucacaaggcuagauuuccaaacaaacgcaucuaa
mle-mir-124	9100	9100	0	0	-	blast	uaaggcacgcggugaaugcc<br>uaaggcacgcggugaaugc<br>uaaggcacgcggugaaugcca<br>uaaggcacgcggugaaugcca<br>uaaggcacgcggugaaugcc<br>uaaggcacgcggugaaugcca<br>uaaggcacgcggugaaugcca<br>uaaggcacgcggugaaugcca<br>uaaggcacgcggugaaugcca<br>uaaggcacgcggugaaugcca<br>uaaggcacgcggugaaugcca<br>uaaggcacgcggugaaugcca<br>guguucacuguguaagccuuggu<br>uaaggcacgcggugaaugcca<br>uaaggcacgcggugaaugcca	-	guguucacuguguaagccuuggugugugcacgauuaaggcacgcggugaaugcca
sja-mir-124	111	111	0	0	-	blast	uaaggcacgcggugaauguca<br>ccauuuuccgcgauugccuugauuu<br>uuaaggcacgcggugaauguca<br>uaaggcacgcggugaauguca	-	uauauguaugccauuuuccgcgauugccuugauuuguuaaaagaaaaugauucacaacaaaauauuaaggcacgcggugaaugucauccacggugc
emu-mir-124b	6	6	0	0	-	blast	uaaggcacgcggugaauacc<br>uaaggcacgcggugaauacc<br>uaaggcacgcggugaauacc<br>guauucuacgcgaugucuuggua<br>guauucuacgcgaugucuuggu	-	gugaauuugguauucuacgcgaugucuugguacgagcugacacuaaggcacgcggugaauaccaaggucc
crm-mir-87	12	12	0	0	-	blast	gugagcaaaguuucaggugugc<br>gugagcaaaguuucaggugugc<br>gugagcaaaguuucaggugugc<br>gugagcaaaguuucaggugugc<br>gugagcaaaguuucaggugu<br>gugagcaaaguuucaggugugc<br>gugagcaaaguuucaggugu<br>gugagcaaaguuucaggugugc<br>gugagcaaaguuucaggugugc<br>gugagcaaaguuucaggugugc<br>gugagcaaaguuucaggugu<br>cgccugacacuuucgucucaaccu<br>gugagcaaaguuucaggugugc	-	cagguugugccuuccggccgccugacacuuucgucucaaccucgcugucugauauagucguaggugagcaaaguuucaggugugccggaacacauccu
hpo-mir-9	6608	6608	0	0	-	blast	ucuuugguuaucuagcuguauga<br>ucuuugguuaucuagcuguauga<br>ucuuugguuaucuagcuguauga<br>ucuuugguuaucuagcuguauga<br>cuuugguuaucuagcuguauga<br>ucuuugguuaucuagcuguauga<br>ucuuugguuaucuagcuguauga<br>auaaggcuggacgacuaaaggaa<br>ucuuugguuaucuagcuguaug	-	gucauugaucucuuugguuaucuagcuguaugauuuuucguacucauaaggcuggacgacuaaaggaaucaaucguc
gsa-mir-87	12	12	0	0	-	blast	gugagcaaaguuucaggugu<br>gugagcaaaguuucaggugugc<br>gugagcaaaguuucaggugugc<br>gugagcaaaguuucaggugugc<br>gugagcaaaguuucaggugugc<br>gugagcaaaguuucaggugugc<br>gugagcaaaguuucaggugu<br>gugagcaaaguuucaggugugc<br>gugagcaaaguuucaggugugc<br>gugagcaaaguuucaggugugc<br>gugagcaaaguuucaggugu<br>gugagcaaaguuucaggugugc<br>acgccugauacuaggcucuuacca	-	acgccugauacuaggcucuuaccaucagaguugauagaaagaauauuucuguaucgaaggaauugauuuucccgcaauacuaaaaucuuuuuacuuuuggugagcaaaguuucaggugugc
mle-mir-9	6608	6608	0	0	-	blast	cuuugguuaucuagcuguauga<br>ucuuugguuaucuagcuguauga<br>auaaagcuagguuaccaaaggca<br>auaaagcuagguuaccaaaggc<br>ucuuugguuaucuagcuguauga<br>ucuuugguuaucuagcuguauga<br>ucuuugguuaucuagcuguauga<br>ucuuugguuaucuagcuguaug<br>ucuuugguuaucuagcuguauga<br>ucuuugguuaucuagcuguauga	-	ucuuugguuaucuagcuguaugauugcccgaaaccaucauaaagcuagguuaccaaaggca
cel-mir-124	6930	6930	0	0	-	blast	uaaggcacgcggugaaugcca<br>uaaggcacgcggugaaugcca<br>uaaggcacgcggugaaugcca<br>uaaggcacgcggugaaugcca<br>uaaggcacgcggugaaugcca<br>uaaggcacgcggugaaugcca<br>uaaggcacgcggugaaugcca<br>uaaggcacgcggugaaugcc<br>uaaggcacgcggugaaugc<br>uaaggcacgcggugaaugcca<br>uaaggcacgcggugaaugcca<br>uaaggcacgcggugaaugcc<br>uaaggcacgcggugaaugcca<br>uaaggcacgcggugaaugcca<br>gcaugcacccuagugacuuuagu	-	gucccacuugucaucuggcaugcacccuagugacuuuaguggacaucuaagucuuccaacuaaggcacgcggugaaugccacguggccaugaugg
hpo-mir-34	3	3	0	0	-	blast	uggcagugugguuagcugguug<br>uggcagugugguuagcugguug<br>uggcagugugguuagcugguug<br>acggcuaucaacacugccgucc	-	uggcagugugguuagcugguugcagccggugacaacggcuaucaacacugccgucc
asu-mir-124	8670	8670	0	0	-	blast	uaaggcacgcggugaaugcca<br>uaaggcacgcggugaaugccaau<br>uaaggcacgcggugaaugcca<br>uaaggcacgcggugaaugcca<br>uaaggcacgcggugaaugcca<br>uaaggcacgcggugaaugcca<br>uaaggcacgcggugaaugccaa<br>uaaggcacgcggugaaugcca<br>cgccuucaccggugacuuuggu<br>uaaggcacgcggugaaugcca<br>uaaggcacgcggugaaugcca<br>uaaggcacgcggugaaugcca<br>uaaggcacgcggugaaugcc<br>uaaggcacgcggugaaugcca<br>uaaggcacgcggugaaugcca<br>uaaggcacgcggugaaugc<br>uaaggcacgcggugaaugccaa<br>uaaggcacgcggugaaugcc	-	uuugcgacacgccuucaccggugacuuuggugugaagcagccuacuaaggcacgcggugaaugccaaucgcucuug
bma-let-7	6000	6000	0	0	-	blast	ugagguaguagguuguauaguu<br>cuguccagccugcuagcuugcc<br>ugagguaguagguuguauaguu<br>ugagguaguagguuguauaguu<br>ugagguaguagguuguauagu<br>ugagguaguagguuguauaguu<br>ugagguaguagguuguauaguu<br>ugagguaguagguuguauaguu<br>ugagguaguagguuguauaguu<br>ugagguaguagguuguauaguu<br>ugagguaguagguuguauaguu	-	augcuaacgacccugaaugauagcuugcucgaugagguaguagguuguauaguuauguaguuguaccauaaucggagaaacuguccagccugcuagcuugccgugcgggucuaucauccgucccc
hpo-mir-100	1880	1880	0	0	-	blast	aacccguagauccgaacuugugu<br>aacccguagauccgaacuuguguu<br>acaggcucggcuacugguaga<br>aacccguagauccgaacuugug<br>aacccguagauccgaacuugug<br>aacccguagauccgaacuuguguu	-	aacccguagauccgaacuuguguucaucauugugcuacacaggcucggcuacugguaga
asu-mir-34	7	7	0	0	-	blast	uggcagugugguuagcugguugu<br>uggcagugugguuagcugguugu<br>uggcagugugguuagcugguug<br>uggcagugugguuagcugguugu<br>uggcagugugguuagcugguug<br>uggcagugugguuagcugguug<br>aacggcuaucaccacugccgucc<br>uggcagugugguuagcugguugu	-	gcggauaagucaccggcuggcaguguggguuagcugguugucagaugugagacaacggcuaucaccacugccguccugguggguucguccauccg
ppc-mir-63a	4	0	0	4	-	blast	uaugacacugaagcgagauggu	-	ucucuccccucuaaucccccuuagccguccuucucggcuuugguugucauugucuacuugaagagaacuaugacacugaagcgagaugguacggcgaggggaggaggaagagggagg
sme-mir-2152	1	0	0	1	-	blast	ggguucgaaguaugucucucuuu	-	cuuauucucaaacuuuaaaggguucgaaguaugucucucuuuauuggagauauauucgaauacgaaugacuagugagccauuuuu
hpo-mir-124	6930	6930	0	0	-	blast	uaaggcacgcggugaaugcca<br>uaaggcacgcggugaaugcc<br>uaaggcacgcggugaaugcca<br>uaaggcacgcggugaaugcc<br>uaaggcacgcggugaaugc<br>uaaggcacgcggugaaugcca<br>uaaggcacgcggugaaugcca<br>uaaggcacgcggugaaugcca<br>uaaggcacgcggugaaugcca<br>uaaggcacgcggugaaugcca<br>cgcuuucaucggugacuuuagu<br>uaaggcacgcggugaaugcca<br>uaaggcacgcggugaaugcca<br>uaaggcacgcggugaaugcca<br>uaaggcacgcggugaaugcca	-	cgcuuucaucggugacuuuagugugaacugcaacuaaggcacgcggugaaugcca
hpo-mir-71	9	9	0	0	-	blast	ugaaagacauggguagugagacg<br>ugaaagacauggguaguga<br>uaucacuauucuguuuuucgc<br>ugaaagacauggguagugagac<br>uaucacuauucuguuuuucgcc<br>uaucacuauucuguuuuucgcc<br>ugaaagacauggguagugagacg<br>ugaaagacauggguagugagac<br>ugaaagacauggguagugagacg<br>uaucacuauucuguuuuucgcc<br>ugaaagacauggguagugagacg<br>ugaaagacauggguagugagac<br>ugaaagacauggguaguga	-	ugaaagacauggguagugagacgcuuuguucuggccguaucacuauucuguuuuucgcc
cel-mir-87	12	12	0	0	-	blast	gugagcaaaguuucaggugu<br>gugagcaaaguuucaggugugc<br>gugagcaaaguuucaggugugc<br>gugagcaaaguuucaggugugc<br>gugagcaaaguuucaggugugc<br>cgccugauacuuucgucucaaccu<br>gugagcaaaguuucaggugugc<br>gugagcaaaguuucaggugu<br>gugagcaaaguuucaggugugc<br>gugagcaaaguuucaggugugc<br>gugagcaaaguuucaggugugc<br>gugagcaaaguuucaggugugc<br>gugagcaaaguuucaggugu	-	gguugugccauccggccgccugauacuuucgucucaaccucgcugucagauuggucguaggugagcaaaguuucaggugugccggaacacaccc
hpo-mir-10157	2	0	0	2	-	blast	cacacuccguggagacgggac<br>aucccgccccuaggugugugu	-	aucccgccccuaggugugugugagaaaggcaacacacuccguggagacgggac
crm-mir-1	40	40	0	0	-	blast	uggaauguaaagaaguaugua<br>uggaauguaaagaaguauguag<br>cauacuuccuuacaugcccau<br>uggaauguaaagaaguaugua<br>uggaauguaaagaaguaugua<br>uggaauguaaagaaguauguag<br>uggaauguaaagaaguaugu<br>uggaauguaaagaaguauguag<br>cauacuuccuuacaugcccaua<br>uggaauguaaagaaguaugu<br>uggaauguaaagaaguaugua<br>uggaauguaaagaaguaugua	-	gcugugccgagcugcauacuuccuuacaugcccauacuauaucaguaauggauauggaauguaaagaaguauguagaacgguuuugaaaacggcu
cel-mir-72	12	12	0	0	-	blast	aggcaagauguuggcauagcu<br>aggcaagauguuggcauagcuga<br>aggcaagauguuggcauagcug<br>aggcaagauguuggcauagcuga<br>aggcaagauguuggcauagcuga<br>aggcaagauguuggcauagcuga<br>aggcaagauguuggcauagcuga<br>aggcaagauguuggcauagcuga<br>aggcaagauguuggcauagcug<br>aggcaagauguuggcauagcu<br>agcuucgccacauucugccacg<br>aggcaagauguuggcauagcuga<br>aggcaagauguuggcauagcug	-	agguccgucagagcuaggcaagauguuggcauagcugaaugaucgcuauaacaacuaucagcuucgccacauucugccacgacugaugugaggaccu
asu-mir-100a-1	1890	1890	0	0	-	blast	aacccguagauccgaacuuguguu<br>cgcaauucgccucucgggugca<br>aacccguagauccgaacuugugu<br>aacccguagauccgaacuuguguu<br>aacccguagauccgaacuugug<br>aacccguagauccgaacuugug	-	ggcgguaaacccguagauccgaacuuguguuguguuauuugguaacgcaauucgccucucgggugcaucgcugauuag
asu-mir-1	40	40	0	0	-	blast	uggaauguaaagaaguaugu<br>uggaauguaaagaaguaugua<br>uggaauguaaagaaguaugua<br>cguacuucuuuacaucgccaua<br>uggaauguaaagaaguaugu<br>uggaauguaaagaaguauguag<br>uggaauguaaagaaguauguag<br>uggaauguaaagaaguaugua<br>uggaauguaaagaaguaugua<br>uggaauguaaagaaguauguag<br>uggaauguaaagaaguaugua	-	uucuguugcaucguacuucuuuacaucgccauacugguauuucaaagagcuauggaauguaaagaaguauguaguuacugaa
emu-mir-124a	10768	10768	0	0	-	blast	uaaggcacgcggugaaugcca<br>uaaggcacgcggugaaugcca<br>uaaggcacgcggugaaugccaa<br>uaaggcacgcggugaaugcca<br>uaaggcacgcggugaaugcca<br>uaaggcacgcggugaaugcca<br>uaaggcacgcggugaaugcca<br>gcguucaacccucggagccuuuguc<br>uaaggcacgcggugaaugcca<br>gcguucaacccucggagccuuuguc<br>uaaggcacgcggugaaugccaa<br>uaaggcacgcggugaaugc<br>uaaggcacgcggugaaugcc<br>uaaggcacgcggugaaugcca<br>uaaggcacgcggugaaugcca<br>uaaggcacgcggugaaugcca<br>uaaggcacgcggugaaugcca<br>uaaggcacgcggugaaugcc	-	ugucaagcacgugaaauggcguucaacccucggagccuuugucagcgauugaauuaaggcacgcggugaaugccaaaugcgaugcucc
asu-mir-72	12	12	0	0	-	blast	aggcaagauguuggcauagcuga<br>aggcaagauguuggcauagcu<br>aggcaagauguuggcauagcug<br>aggcaagauguuggcauagcuga<br>aggcaagauguuggcauagcuga<br>aggcaagauguuggcauagcuga<br>aggcaagauguuggcauagcuga<br>aggcaagauguuggcauagcuga<br>agccgugcuacauaaggccaugc<br>aggcaagauguuggcauagcu<br>aggcaagauguuggcauagcug<br>aggcaagauguuggcauagcug<br>aggcaagauguuggcauagcuga	-	cgugggaccaggcaagauguuggcauagcugaacgaagcuucggauucagccgugcuacauaaggccaugcucccgcg
hpo-mir-72	12	12	0	0	-	blast	aggcaagauguuggcauagcug<br>aggcaagauguuggcauagcu<br>agcuaugcuacaugaggccacg<br>aggcaagauguuggcauagcuga<br>aggcaagauguuggcauagcuga<br>aggcaagauguuggcauagcuga<br>aggcaagauguuggcauagcug<br>aggcaagauguuggcauagcuga<br>aggcaagauguuggcauagcuga<br>aggcaagauguuggcauagcu<br>aggcaagauguuggcauagcuga<br>aggcaagauguuggcauagcuga<br>aggcaagauguuggcauagcug	-	aggcaagauguuggcauagcugaagcacuguuucucagcuaugcuacaugaggccacg
mco-mir-87	12	12	0	0	-	blast	gugagcaaaguuucaggugu<br>gugagcaaaguuucaggugugc<br>ccaccugcuauuuugcucgaucc<br>gugagcaaaguuucaggugugc<br>gugagcaaaguuucaggugugc<br>gugagcaaaguuucaggugugc<br>gugagcaaaguuucaggugugc<br>gugagcaaaguuucaggugu<br>gugagcaaaguuucaggugugc<br>gugagcaaaguuucaggugugc<br>gugagcaaaguuucaggugugc<br>gugagcaaaguuucaggugu<br>gugagcaaaguuucaggugugc	-	ccaccugcuauuuugcucgauccagaguuuagccuagcgucuuucuucucaagaggaaugaacgcuggguuggugagcaaaguuucaggugugc
hpo-mir-87a	12	12	0	0	-	blast	gugagcaaaguuucaggugugc<br>gugagcaaaguuucaggugu<br>gugagcaaaguuucaggugugc<br>gugagcaaaguuucaggugugc<br>gugagcaaaguuucaggugugc<br>gugagcaaaguuucaggugugc<br>gugagcaaaguuucaggugu<br>gugagcaaaguuucaggugu<br>gugagcaaaguuucaggugugc<br>gugagcaaaguuucaggugugc<br>gugagcaaaguuucaggugugc<br>gugagcaaaguuucaggugugc<br>cgccugaaccuuucgucucaaccu	-	cgccugaaccuuucgucucaaccucuaugucaggcaggugagcaaaguuucaggugugc
mle-mir-33	1	0	0	1	-	blast	caaugucucugcaauguaauca	-	gugcauuguaguugcauugcgaggaauuuaauuuuugcaaugucucugcaauguaauca
cel-let-7	6710	6710	0	0	-	blast	ugagguaguagguuguauaguu<br>cuaugcaauuuucuaccuuacc<br>ugagguaguagguuguauaguu<br>ugagguaguagguuguauaguu<br>ugagguaguagguuguauaguuu<br>ugagguaguagguuguauaguu<br>ugagguaguagguuguauaguu<br>ugagguaguagguuguauaguu<br>ugagguaguagguuguauaguu<br>ugagguaguagguuguauaguu<br>ugagguaguagguuguauagu<br>ugagguaguagguuguauaguu	-	uacacuguggauccggugagguaguagguuguauaguuuggaauauuaccaccggugaacuaugcaauuuucuaccuuaccggagacagaacucuucga
asu-mir-100a-2	1885	1885	0	0	-	blast	aacccguagauccgaacuugugu<br>aacccguagauccgaacuuguguu<br>aacccguagauccgaacuugug<br>aacccguagauccgaacuugug<br>aacccguagauccgaacuuguguu<br>caugagcucggcacucggguaga	-	cgauucauuguguacugaagcaggggugugugcggucaacccguagauccgaacuuguguugggguuuugguaacaugagcucggcacucgggguagacugcgucaucccagcccgccacccaaugcccg
prd-mir-71	9	9	0	0	-	blast	ugaaagacauggguagugagacg<br>ugucacuauucuguuuuucgcc<br>ugaaagacauggguagugagac<br>ugaaagacauggguagugagacg<br>ugaaagacauggguaguga<br>ugaaagacauggguagugagacg<br>ugaaagacauggguagugagac<br>ugaaagacauggguaguga<br>ugaaagacauggguagugagacg<br>ugaaagacauggguagugagac	-	ugaaagacauggguagugagacgugugcucugcgaucgugucacuauucuguuuuucgcc
sme-mir-31b-1	1	1	0	0	-	blast	aggcaagaugcuggcauagcuga<br>cagcuauucaggaucuuuccccu	-	auugauaaugacaaggcaagaugcuggcauagcugauaaacuauuuauuaccagcuauucaggaucuuuccccugaauauaucaau
asu-mir-9	6584	6584	0	0	-	blast	cuuugguuaucuagcuguauga<br>auaaagcuggacgaccgaaguaa<br>ucuuugguuaucuagcuguauga<br>ucuuugguuaucuagcuguauga<br>ucuuugguuaucuagcuguauga<br>ucuuugguuaucuagcuguauga<br>ucuuugguuaucuagcuguaug<br>ucuuugguuaucuagcuguauga<br>ucuuugguuaucuagcuguauga	-	gcaauuucuuugguuaucuagcuguaugaguaccgcguuuagucgucauaaagcuggacgaccgaaguaauggccgcu
mco-mir-124b	12	0	0	12	-	blast	guauucuacgcggaugucuuggug	-	guauucuacgcggaugucuugguguggagcagacauuaaggcacgcggugaauac
							uaaggcacgcggugaaugcc<br>uaaggcacgcggugaaugc<br>uaaggcacgcggugaaugcca<br>uaaggcacgcggugaaugcca<br>uaaggcacgcggugaaugcca<br>uaaggcacgcggugaaugcca<br>uaaggcacgcggugaaugcc		

sme-mir-124c-1	6958	6958	0	0	-	blast	uaaggcacgcggugaaugcca<br>uaaggcacgcggugaaugcca<br>gcgcucaccucgugaccuuugu<br>uaaggcacgcggugaaugcca<br>uaaggcacgcggugaaugcca<br>uaaggcacgcggugaaugcca<br>uaaggcacgcggugaaugcca<br>uaaggcacgcggugaaugcca	-	ugauccuauucuuuacuagcgcucaccucgugaccuuugucaguguuggacuaaggcacgcggugaaugccagaugaaaggauga
prd-mir-34	7	7	0	0	-	blast	uggcagugugguuagcugguugu<br>uggcagugugguuagcugguug<br>uggcagugugguuagcugguug<br>uggcagugugguuagcugguugu<br>uggcagugugguuagcugguugu<br>uggcagugugguuagcugguugu<br>acggcuaucuggcacugcccgcc<br>uggcagugugguuagcugguug	-	uggcagugugguuagcugguuguuguuaaugaccaguucaacggcuaucuggcacugcccgcc
prd-mir-72	12	12	0	0	-	blast	aggcaagauguuggcauagcuga<br>aggcaagauguuggcauagcug<br>gccaugcuacauagggccaug<br>aggcaagauguuggcauagcu<br>aggcaagauguuggcauagcuga<br>aggcaagauguuggcauagcuga<br>aggcaagauguuggcauagcuga<br>aggcaagauguuggcauagcug<br>aggcaagauguuggcauagcuga<br>aggcaagauguuggcauagcuga<br>aggcaagauguuggcauagcuga<br>aggcaagauguuggcauagcug<br>aggcaagauguuggcauagcu	-	aggcaagauguuggcauagcugagucccauucaauauuuguaucucagccaugcuacauagggccaug
asu-mir-92	16	16	0	0	-	blast	uauugcacucgucccggccuga<br>uauugcacucgucccggccug<br>aggccgagucgucugcauuauu<br>uauugcacucgucccggccu<br>uauugcacucgucccggccuga	-	gacacagucaggccgagucgucugcauuauucggaagcagcaaacggcaauauugcacucgucccggccugagugcgcguuca
mle-mir-31	12	12	0	0	-	blast	aggcaagauguuggcauagcug<br>aggcaagauguuggcauagcuga<br>aggcaagauguuggcauagcuga<br>aggcaagauguuggcauagcu<br>aggcaagauguuggcauagcug<br>aggcaagauguuggcauagcuga<br>aggcaagauguuggcauagcuga<br>aggcaagauguuggcauagcuga<br>aggcaagauguuggcauagcuga<br>aggcaagauguuggcauagcuga<br>agcugugucauauguugccaau<br>aggcaagauguuggcauagcug<br>aggcaagauguuggcauagcu	-	aggcaagauguuggcauagcugaguauuguacacuucagcugugucauauguugccaau
hpo-let-7	5830	5830	0	0	-	blast	ugagguaguagguuguauaguu<br>ugagguaguagguuguauaguu<br>ugagguaguagguuguauagu<br>ugagguaguagguuguauaguu<br>cuauuacaacuugcuagcuugc<br>ugagguaguagguuguauaguu<br>ugagguaguagguuguauaguu<br>ugagguaguagguuguauaguu<br>ugagguaguagguuguauaguu<br>ugagguaguagguuguauaguu<br>ugagguaguagguuguauaguu	-	ugagguaguagguuguauaguucagcguggcaucaucaggugaaacuauuacaacuugcuagcuugc
bma-mir-750	1	0	0	1	-	blast	ccagaucuguccuucggcuaac	-	acaaguuugugucagucggagguagggucuuaggcauugcuaaugcgacguugccagaucuguccuucggcuaacauuggcuuau
mco-mir-124a	6930	6930	0	0	-	blast	uaaggcacgcggugaaugcca<br>uaaggcacgcggugaaugcca<br>uaaggcacgcggugaaugcca<br>uaaggcacgcggugaaugcca<br>uaaggcacgcggugaaugcca<br>uaaggcacgcggugaaugcca<br>uaaggcacgcggugaaugcca<br>uaaggcacgcggugaaugcc<br>uaaggcacgcggugaaugc<br>uaaggcacgcggugaaugcca<br>uaaggcacgcggugaaugcca<br>gcguuuauccgcgugacuuuaau<br>uaaggcacgcggugaaugcc<br>uaaggcacgcggugaaugcca<br>uaaggcacgcggugaaugcca	-	gcguuuauccgcgugacuuuaauucgaaccggaaauaaggcacgcggugaaugcca
mle-mir-34	7	7	0	0	-	blast	uggcagugugguuagcugguugu<br>uggcagugugguuagcugguug<br>uggcagugugguuagcugguug<br>aaccacugauuacacuuccuug<br>uggcagugugguuagcugguugu<br>uggcagugugguuagcugguugu<br>uggcagugugguuagcugguugu<br>uggcagugugguuagcugguug	-	uggcagugugguuagcugguuguguauccuuuaccauuacaaccacugauuacacuuccuug
crm-mir-72	12	12	0	0	-	blast	aggcaagauguuggcauagcug<br>aggcaagauguuggcauagcuga<br>aggcaagauguuggcauagcuga<br>aggcaagauguuggcauagcuga<br>aggcaagauguuggcauagcu<br>aggcaagauguuggcauagcu<br>aggcaagauguuggcauagcug<br>aggcaagauguuggcauagcuga<br>aggcaagauguuggcauagcuga<br>aggcaagauguuggcauagcuga<br>agcucggccacauacugccacg<br>aggcaagauguuggcauagcug<br>aggcaagauguuggcauagcuga	-	ccguaagacucgucaaagcuaggcaagauguuggcauagcugaaugcaauuuggcgcucagcucggccacauacugccacgcauuggcuagagucuaauuuuuaucccuaugg
