# Supplementary material for: A thermo‐resistant and RNase‐sensitive cargo from Giardia duodenalis extracellular vesicles modifies the behaviour of enterobacteria
Source: J Extracell Biol. 2023 Aug 30;2(9):e109. doi: 10.1002/jex2.109 (PMC11080815; doi:10.1002/jex2.109)
Supplement: Supplementary file 3 — Supplementary Information [file JEX2-2-e109-s008.pdf]

miRDeep2

Parameters used

miRDeep2 version	2.0.1.3
Program call	/home/users/allstaff/balan.b/.conda/envs/miRNA_New/bin/miRDeep2.pl ./LCS8251_AT_Bile_RawData_trim_collapsed.fa ./GiardiaDB-54_GintestinalisAssemblageAWB_Genome_Thibult_nowhitespace.fasta ./Bile_vs_genome.arf Mature.fa none Hairpin.fa
Reads	./LCS8251_AT_Bile_RawData_trim_collapsed.fa
Genome	./GiardiaDB-54_GintestinalisAssemblageAWB_Genome_Thibult_nowhitespace.fasta
Mappings	./Bile_vs_genome.arf
Reference mature miRNAs	Mature.fa
Other mature miRNAs	none

Survey of miRDeep2 performance for score cut-offs -10 to 10

	novel miRNAs			known miRBase miRNAs				
miRDeep2 score	predicted by miRDeep2	estimated false positives	estimated true positives	in species	in data	detected by miRDeep2	estimated signal-to-noise	excision gearing
10	3	0 ± 1	3 ± 1 (86 ± 23%)	4574	0	0 (0%)	7	1
9	3	0 ± 1	3 ± 1 (85 ± 25%)	4574	0	0 (0%)	6.5	1
8	3	0 ± 1	3 ± 1 (85 ± 25%)	4574	0	0 (0%)	6.5	1
7	3	0 ± 1	3 ± 1 (85 ± 25%)	4574	0	0 (0%)	6.5	1
6	3	0 ± 1	3 ± 1 (84 ± 25%)	4574	0	0 (0%)	6.4	1
5	3	1 ± 1	3 ± 1 (84 ± 25%)	4574	0	0 (0%)	6	1
4	3	1 ± 1	2 ± 1 (82 ± 26%)	4574	0	0 (0%)	5.6	1
3	3	1 ± 1	2 ± 1 (79 ± 30%)	4574	0	0 (0%)	4.7	1
2	6	1 ± 1	5 ± 1 (82 ± 20%)	4574	0	0 (0%)	5.5	1
1	7	3 ± 2	4 ± 2 (57 ± 25%)	4574	0	0 (0%)	2.3	1
0	8	11 ± 3	0 ± 1 (2 ± 7%)	4574	0	0 (0%)	0.7	1
-1	8	17 ± 4	0 ± 0 (0 ± 0%)	4574	0	0 (0%)	0.5	1
-2	14	22 ± 4	0 ± 0 (0 ± 0%)	4574	0	0 (0%)	0.6	1
-3	33	31 ± 5	3 ± 3 (9 ± 10%)	4574	0	0 (0%)	1.1	1
-4	68	70 ± 8	2 ± 4 (3 ± 6%)	4574	0	0 (0%)	1	1
-5	79	260 ± 13	0 ± 0 (0 ± 0%)	4574	0	0 (0%)	0.3	1
-6	97	424 ± 15	0 ± 0 (0 ± 0%)	4574	0	0 (0%)	0.2	1
-7	190	535 ± 16	0 ± 0 (0 ± 0%)	4574	0	0 (0%)	0.4	1
-8	406	610 ± 18	0 ± 0 (0 ± 0%)	4574	0	0 (0%)	0.7	1
-9	531	655 ± 19	0 ± 0 (0 ± 0%)	4574	0	0 (0%)	0.8	1
-10	622	683 ± 19	0 ± 0 (0 ± 0%)	4574	0	0 (0%)	0.9	1

novel miRNAs predicted by miRDeep2

provisional id	miRDeep2 score	estimated probability that the miRNA candidate is a true positive	rfam alert	total read count	mature read count	loop read count	star read count	significant randfold p-value	miRBase miRNA	example miRBase miRNA with the same seed	UCSC browser	NCBI blastn	consensus mature sequence	consensus star sequence	consensus precursor sequence	precursor coordinate
GLCHR01_392	6.2e+2	0.86 ± 0.23		1235	1232	0	3	no				blast	aggggggacccccgacacggc	acggagggggcggcccccgugcc	aggggggacccccgacacggcaugggcacggagggggcggcccccgugcc	GLCHR01:1469000..1469050:+
CH991838_9834	6.2e+2	0.86 ± 0.23		1235	1232	0	3	no				blast	aggggggacccccgacacggc	acggagggggcggcccccgugcc	aggggggacccccgacacggcaugggcacggagggggcggcccccgugcc	CH991838:1061..1111:+
AACB02000118_9113	6.2e+2	0.86 ± 0.23		1235	1232	0	3	no				blast	aggggggacccccgacacggc	acggagggggcggcccccgugcc	aggggggacccccgacacggcaugggcacggagggggcggcccccgugcc	AACB02000118:653..703:-
CH991778_9719	2.4	0.82 ± 0.20		203	164	34	5	yes				blast	uggacagcgaucgaggggg	uggcaugcugucccccac	uggacagcgaucgaggggggccgauggccaugcacacugccugccaccgagucacuccccuggcaugcugucccccac	CH991778:37664..37742:-
GLCHR05_8104	2.4	0.82 ± 0.20		151	151	0	0	yes				blast	aggaggaggaugaggaaggu	cugucuuucuagcugcuug	cugucuuucuagcugcuugucgcgcuucagcucgcagcaguggagacugauugugacgaggaggaggaugaggaaggu	GLCHR05:3997811..3997889:-
GLCHR04_3046	2.1	0.82 ± 0.20		24	24	0	0	yes				blast	guaggguagggguagggua	acuuaccucuagacuacga	guaggguaggguaggguaaacacuuaccucuagacuacga	GLCHR04:1031437..1031477:+
GLCHR03_2717	1.5	0.57 ± 0.25		141	141	0	0	yes				blast	ggaagaagaagaggagga	gucugugucagucagcucc	ggaagaagaagaggaggacgggguugucucuuucuggucgucugugucagucagcucc	GLCHR03:1550608..1550666:-
AACB02000160_9277	0	0.02 ± 0.07		6	4	0	2	no				blast	ggcuuccucugcuggugg	ucugucgggggaaggcgua	ggcuuccucugcugguggugguucaucugucgggggaaggcgua	AACB02000160:1402..1443:+

mature miRBase miRNAs detected by miRDeep2

tag id	miRDeep2 score	estimated probability that the miRNA is a true positive	rfam alert	predicted mature seq. in accordance with miRBase mature seq.	total read count	mature read count	loop read count	star read count	significant randfold p-value	mature miRBase miRNA	example miRBase miRNA with the same seed	UCSC browser	NCBI blastn	consensus mature sequence	consensus star sequence	consensus precursor sequence	precursor coordinate

mature miRBase miRNAs not detected by miRDeep2

miRBase precursor id	total read count	mature read count(s)	star read count	remaining reads	UCSC browser	NCBI blastn	miRBase mature sequence(s)	miRBase star sequence(s)	miRBase precursor sequence
prd-let-7	1606	1606	0	0	-	blast	ugagguaguagguuguauaguu<br>ugagguaguagguuguauaguu<br>ugagguaguagguuguauaguu<br>ugagguaguagguuguauagu<br>ugagguaguagguuguauaguu<br>acugucaaucuacuaacuuaac<br>ugagguaguagguuguauaguu<br>ugagguaguagguuguauaguu<br>ugagguaguagguuguauaguuu<br>ugagguaguagguuguauaguu<br>ugagguaguagguuguauaguu<br>ugagguaguagguuguauaguu	-	ugagguaguagguuguauaguuuuugauuguauaaaaacugucaaucuacuaacuuaac
ppc-mir-9	504	504	0	0	-	blast	ucuuugguuaucuagcuguauga<br>ucuuugguuaucuagcuguauga<br>ucuuugguuaucuagcuguauga<br>ucuuugguuaucuagcuguauga<br>ucuuugguuaucuagcuguauga<br>cuuugguuaucuagcuguauga<br>ucuuugguuaucuagcuguaug<br>ucuuugguuaucuagcuguauga	-	uuccuucaaugggacagucauuaucgucucuuugguuaucuagcuguaugauuugaaauguaaaucauaaagcuggaaaacugaaggaacgaugugucucaugga
sme-mir-124c-2	518	518	0	0	-	blast	uaaggcacgcggugaaugcc<br>uaaggcacgcggugaaugcca<br>uaaggcacgcggugaaugcca<br>uaaggcacgcggugaaugcca<br>uaaggcacgcggugaaugcca<br>uaaggcacgcggugaaugcca<br>uaaggcacgcggugaaugc<br>uaaggcacgcggugaaugcca<br>uaaggcacgcggugaaugcca<br>gcauuaacccuguugucuuagau<br>uaaggcacgcggugaaugcca<br>uaaggcacgcggugaaugcca<br>uaaggcacgcggugaaugcca<br>uaaggcacgcggugaaugcca<br>uaaggcacgcggugaaugcc	-	ccauuauauucaugaagcauuaacccuguugucuuagauaguuuauuacaaaacauuuaaggcacgcggugaaugccaugugaaaugg
prd-mir-100	140	140	0	0	-	blast	aacccguagauccgaacuugug<br>aacccguagauccgaacuuguguu<br>cacaucuucggguaugggucgg<br>aacccguagauccgaacuugug<br>aacccguagauccgaacuuguguu<br>aacccguagauccgaacuugugu	-	aacccguagauccgaacuuguguucugucaacaagaaacacaucuucggguaugggucgg
prd-mir-124	476	476	0	0	-	blast	ggguuuucacggugacuucagu<br>uaaggcacgcggugaaugcca<br>uaaggcacgcggugaaugcca<br>uaaggcacgcggugaaugcca<br>uaaggcacgcggugaaugccaa<br>uaaggcacgcggugaaugccaa<br>uaaggcacgcggugaaugcca<br>uaaggcacgcggugaaugcca<br>uaaggcacgcggugaaugcc<br>uaaggcacgcggugaaugcca<br>uaaggcacgcggugaaugcca<br>uaaggcacgcggugaaugcca<br>uaaggcacgcggugaaugcc<br>uaaggcacgcggugaaugc<br>uaaggcacgcggugaaugcca<br>uaaggcacgcggugaaugcca<br>uaaggcacgcggugaaugcca<br>uaaggcacgcggugaaugccaau	-	ggguuuucacggugacuucagugagaauuaaaucaacuaaggcacgcggugaaugccaau
cel-mir-8210	27	27	0	0	-	blast	ugccuucuuuccuugugucgccgac<br>cgacgaaaaaaagaaagaaagaaa	-	ugccuucuuuccuugugucgccgacaaaucgccacaauccgucgacgaaaaaaagaaagaaagaaa
lgi-mir-7	26	26	0	0	-	blast	uggaagacuagugauuuuguuguu	-	auaaccuaucuacccuggucuggaagacuagugauuuuguuguuuuaauuucaguauuaacaauaaaucacauucuucccagauugguugauucuuaaca
cte-mir-125	24	24	0	0	-	blast	ucccugagacccuaacuuguga	-	uaaccacucggcaucucgcacucccugagacccuaacuugugaagcuuucuaaaaagaucgcgaguuagaaucuugggcucugcgaggagcucgauugccg
sme-mir-124b	30	30	0	0	-	blast	uaaggcacgcggugaaugcuga<br>uaaggcacgcggugaaugc<br>ugcauuuacaacgugucuuuagu	-	guauccaugaaaauuugcauuuacaacgugucuuuaguucaugugacacuaaggcacgcggugaaugcugaauuaaaugguuua
sme-mir-124a	30	30	0	0	-	blast	uaaggcacgcggugaaugcuu<br>ugcuuuuuaacgcggagcuuuagu<br>uaaggcacgcggugaaugc	-	uucaaucagaugugcuuuuaacgcggagcuuuaguaauuuaacauguauacuaaggcacgcggugaaugcuuacuugauugag
str-mir-124	11	11	0	0	-	blast	cgcuuucauccgugacuuuaga<br>uaaggcacgcggugaaugc	-	cgcuuucauccgugacuuuagaagagucuuauaucacuaaggcacgcggugaaugc
mle-mir-8	30	30	0	0	-	blast	uaauacugucagguaaagauguc<br>uaauacugucagguaaagauguc<br>uaauacugucagguaaagauguc<br>ggucuuaccuagcagcauugga	-	ggucuuaccuagcagcauuggauuguucugcucuaauacugucagguaaagauguc
cte-mir-317	7	7	0	0	-	blast	ugaacacagcugguggguaucucuuu	-	cggaaggagaagcgucgagcgcaggcaagagaucacugcggugcucacauguagccacugugaacacagcugguggguaucucuuuucuucgcucugaccug
mle-bantam	6	6	0	0	-	blast	cugguuuucacugugauuuacaga<br>ugagaucauugugaaaacugauu	-	cugguuuucacugugauuuacagauauuuccaacaauucugagaucauugugaaaacugauu
lgi-mir-10	6	6	0	0	-	blast	uacccuguagauccgaauuugu	-	gaauauugugccuuuacguuuacccuguagauccgaauuuguguaugaaacaaucuacaaauucguuuuugcgggugauugugucgagguuuucaaaauca
mle-mir-184	15	15	0	0	-	blast	uggacggagaacugauaagggc<br>ccuuaucacuuuucccuccugu<br>uggacggagaacugauaagggc<br>uggacggagaacugauaagggc	-	ccuuaucacuuuucccuccuguuuuugauuugaauacuggacggagaacugauaagggc
prd-mir-1	50	50	0	0	-	blast	uggaauguaaagaaguaugua<br>uggaauguaaagaaguauguag<br>uggaauguaaagaaguaugua<br>uggaauguaaagaaguaugu<br>uggaauguaaagaaguaugu<br>acgugcuucuuugcaagaccaua<br>uggaauguaaagaaguaugua<br>uggaauguaaagaaguaugua<br>uggaauguaaagaaguauguag<br>uggaauguaaagaaguaugua<br>uggaauguaaagaaguauguag	-	acgugcuucuuugcaagaccauagugaauacuguuuauggaauguaaagaaguauguag
mle-mir-1	45	45	0	0	-	blast	uggaauguaaagaaguauguau<br>uggaauguaaagaaguaugua<br>uggaauguaaagaaguaugua<br>uggaauguaaagaaguauguau<br>uggaauguaaagaaguaugu<br>uggaauguaaagaaguaugua<br>acauacuuccuugcuaucccaua<br>uggaauguaaagaaguaugu<br>uggaauguaaagaaguaugua<br>uggaauguaaagaaguaugua	-	acauacuuccuugcuaucccauauuuauggcaaccuauggaauguaaagaaguauguau
str-mir-34a	28	28	0	0	-	blast	uggcagugugguuagcugguug<br>uggcagugugguuagcugguugu<br>acagcucacucaacugccaag<br>uggcagugugguuagcugguugu<br>uggcagugugguuagcugguugu<br>uggcagugugguuagcugguugu<br>uggcagugugguuagcugguug<br>uggcagugugguuagcugguug	-	uggcagugugguuagcugguugugauuuuuuagacgcucaacagcucacucaacugccaag
mle-mir-92b	4	4	0	0	-	blast	gggucgagauucuugcaaaag<br>uauugcacuuuucccggccugu	-	gggucgagauucuugcaaaaguuguacaucgacucuuauugcacuuuucccggccugu
sma-mir-124a	6	6	0	0	-	blast	ccauuuuccgcgauugccuugauga<br>uaaggcacgcggugaauguca<br>uuaaggcacgcggugaauguca<br>uaaggcacgcggugaauguca	-	uuuguaugccauuuuccgcgauugccuugaugaguuauaaaauauuauucauaacaaaaauauuaaggcacgcggugaaugucauccacggagc
mle-mir-263a	4	4	0	0	-	blast	aauggcacugguagaaucacgg<br>aauggcacugguagaaucacgg<br>guguucuuccgguggcaucua	-	aauggcacugguagaaucacgguuugcuuuaaaaacuccguguucuuccgguggcaucua
bma-mir-7	2	2	0	0	-	blast	uggaagacuugugauuuuguugu	-	aaaaguuaaagcugaccagguggaagacuugugauuuuguuguuuauacauuuucagcaacagcaaauccgagcucuccacccggaggcau
lgi-mir-317	2	2	0	0	-	blast	ugaacacagcugguggguaucuucu	-	aagagaagauaacucuguguggaggauaccauguuguguuuacaguuuugaaacauuugugaacacagcugguggguaucuucuauaagcagaguauuuca
cte-mir-137	2	2	0	0	-	blast	uauugcuugagaauacacguag<br>uuauugcuugagaauaca	-	aagaagaacgcuagguugaguacggguauucuuggguaaaauaauauaccaagaaggauguuauugcuugagaauacacguagcaaaccuaagcuuuaucga
mle-mir-219	2	2	0	0	-	blast	ugauuguccaaacgcaauucu<br>agaacugugggguggacaucagu<br>ugauuguccaaacgcaauucuug	-	ugauuguccaaacgcaauucuuguuacauccuaaccaagaacugugggguggacaucagu
cte-mir-252a	1	1	0	0	-	blast	cuaaguacuagugccgcaggag	-	augcucgcucauugccaaugcuaaguacuagugccgcaggagguuccuuguucaaccccuccugcuguucgagugcuuaauaucacauuugagcauuuccu
gsa-mir-124b	1	1	0	0	-	blast	uaaggcacgcggugaauauc<br>guguuuaccgugggggucuugau	-	guguuuaccgugggggucuugauuuugguuguauuuauuaaggcacgcggugaauauc
gsa-mir-124a	3	3	0	0	-	blast	uaaggcacgcggugaauacc<br>uaaggcacgcggugaauacc<br>gguauuuuccacgaagucuuagg<br>uaaggcacgcggugaauacc	-	gguauuuuccacgaagucuuaggaaguugaaaaauaaaaauauuuaacuuaaggcacgcggugaauacc
mco-mir-9	2	2	0	0	-	blast	caacgcuagauuuccaaacaaa<br>ucuuugguuaucuagcugugug<br>ucuuugguuaucuagcugugug	-	ucuuugguuaucuagcugugugacagguagcuucaucuggcuucuuuuauauucacaacgcuagauuuccaaacaaa
mle-mir-29b	2	2	0	0	-	blast	uagcaccauuugaaaucagug<br>uagcaccauuugaaaucagug<br>cugauuuccagugguggauaga	-	cugauuuccagugguggauagauguccuuagggcucgagucuugucuagcaccauuugaaaucagug
mle-mir-29a	3	3	0	0	-	blast	uagcaccauuugaaaucaguuu<br>ccugaucucuccuggugcauaga<br>uagcaccauuugaaaucaguuu<br>uagcaccauuugaaaucaguuu	-	ccugaucucuccuggugcauagauuugaucccugcgucuagcaccauuugaaaucaguuu
str-mir-1	1	1	0	0	-	blast	uggaauguaaagaaguaugca<br>caugcuucuuuauagugccaua	-	caugcuucuuuauagugccauauagcaagagaacuuauggaauguaaagaaguaugca
hpo-mir-236	2	2	0	0	-	blast	uaauacugucagguaaugaugcu<br>cuaauacugucagguaaugaugc<br>gucuuaccugaacaauauuaga	-	gucuuaccugaacaauauuagaagcuucgaucugucuaauacugucagguaaugaugcu
mle-mir-375	2	2	0	0	-	blast	acacgagcaguucguaacaaggc<br>uuuguucguucggcucgcguua<br>uuuguucguucggcucgcguua	-	acacgagcaguucguaacaaggccucccauuuagguguuuuguucguucggcucgcguua
mle-mir-317	1	1	0	0	-	blast	agggugccauccugugcucaca<br>ugaacacagcugguggguauccuau	-	agggugccauccugugcucacauguugauaugaacaaagugugugugaacacagcugguggguauccuau
mle-mir-2b	1	1	0	0	-	blast	uaucacagccagcuuugaugagc<br>gcaucaaugccgguguuauagu	-	gcaucaaugccgguguuauaguucgacugcauggcccuaucacagccagcuuugaugagc
hco-mir-5991	1	1	0	0	-	blast	ugagguaguaggugguguucga	-	ucuucauaacauuuuauuuuuagcguugaucgagcuaggcuucuauuugccuuugauuucgcuucgucauugagguaguaggugguguucgauugaugccau
cel-mir-8209	1	1	0	0	-	blast	aaaacgaagaaagaagaagaa<br>ucuucuucuccccuucguuuccc	-	aaaacgaagaaagaagaagaagcauaguguugucuucuucuccccuucguuuccc
asu-mir-1	50	50	0	0	-	blast	uggaauguaaagaaguaugua<br>uggaauguaaagaaguauguag<br>uggaauguaaagaaguaugua<br>uggaauguaaagaaguaugu<br>uggaauguaaagaaguaugu<br>uggaauguaaagaaguaugua<br>uggaauguaaagaaguaugua<br>uggaauguaaagaaguaugua<br>uggaauguaaagaaguauguag<br>uggaauguaaagaaguauguag<br>cguacuucuuuacaucgccaua	-	uucuguugcaucguacuucuuuacaucgccauacugguauuucaaagagcuauggaauguaaagaaguauguaguuacugaa
asu-mir-100a-2	140	140	0	0	-	blast	aacccguagauccgaacuugug<br>aacccguagauccgaacuuguguu<br>aacccguagauccgaacuugug<br>caugagcucggcacucggguaga<br>aacccguagauccgaacuuguguu<br>aacccguagauccgaacuugugu	-	cgauucauuguguacugaagcaggggugugugcggucaacccguagauccgaacuuguguugggguuugguaacaugagcucggcacucggguagacugcgucaucccagcccgccacccaaugcccg
gsa-mir-9393	1	0	0	1	-	blast	gugagggaaugguuuuucgauu<br>ucucuaaacucuuccuuccagu	-	gugagggaaugguuuuucgauugacauaauuucaguuuuauaauucuauaaaaagaaaagaaaacgauaaaaaugcaagaacccuuaaucucuaaacucuuccuuccagu
hpo-mir-1	14	14	0	0	-	blast	uggaauguaaagaaguaugua<br>cgugcuucuuugcaugcccaua<br>uggaauguaaagaaguaugu<br>uggaauguaaagaaguaugua<br>uggaauguaaagaaguaugua<br>uggaauguaaagaaguaugua<br>uggaauguaaagaaguaugu<br>uggaauguaaagaaguaugua	-	cgugcuucuuugcaugcccauacuguauuuugauauggaauguaaagaaguaugua
mle-mir-9	504	504	0	0	-	blast	ucuuugguuaucuagcuguauga<br>ucuuugguuaucuagcuguauga<br>auaaagcuagguuaccaaaggca<br>ucuuugguuaucuagcuguauga<br>ucuuugguuaucuagcuguauga<br>ucuuugguuaucuagcuguauga<br>ucuuugguuaucuagcuguaug<br>cuuugguuaucuagcuguauga<br>auaaagcuagguuaccaaaggc<br>ucuuugguuaucuagcuguauga	-	ucuuugguuaucuagcuguaugauugcccgaaaccaucauaaagcuagguuaccaaaggca
prd-mir-34	7	7	0	0	-	blast	uggcagugugguuagcugguugu<br>uggcagugugguuagcugguugu<br>uggcagugugguuagcugguugu<br>uggcagugugguuagcugguugu<br>uggcagugugguuagcugguug<br>acggcuaucuggcacugcccgcc<br>uggcagugugguuagcugguug<br>uggcagugugguuagcugguug	-	uggcagugugguuagcugguuguuguuaaugaccaguucaacggcuaucuggcacugcccgcc
crm-mir-1	50	50	0	0	-	blast	uggaauguaaagaaguaugua<br>uggaauguaaagaaguaugu<br>uggaauguaaagaaguauguag<br>uggaauguaaagaaguauguag<br>uggaauguaaagaaguaugua<br>uggaauguaaagaaguaugua<br>uggaauguaaagaaguauguag<br>uggaauguaaagaaguaugua<br>uggaauguaaagaaguaugua<br>uggaauguaaagaaguaugu<br>cauacuuccuuacaugcccaua<br>cauacuuccuuacaugcccau	-	gcugugccgagcugcauacuuccuuacaugcccauacuauaucaguaauggauauggaauguaaagaaguauguagaacgguuuugaaaaacggcu
hpo-mir-124	378	378	0	0	-	blast	cgcuuucaucggugacuuuagu<br>uaaggcacgcggugaaugcc<br>uaaggcacgcggugaaugcca<br>uaaggcacgcggugaaugcca<br>uaaggcacgcggugaaugcca<br>uaaggcacgcggugaaugcca<br>uaaggcacgcggugaaugcca<br>uaaggcacgcggugaaugcca<br>uaaggcacgcggugaaugc<br>uaaggcacgcggugaaugcca<br>uaaggcacgcggugaaugcca<br>uaaggcacgcggugaaugcca<br>uaaggcacgcggugaaugcca<br>uaaggcacgcggugaaugcca<br>uaaggcacgcggugaaugcc	-	cgcuuucaucggugacuuuagugugaacugcaacuaaggcacgcggugaaugcca
asu-mir-34	7	7	0	0	-	blast	uggcagugugguuagcugguugu<br>uggcagugugguuagcugguugu<br>uggcagugugguuagcugguugu<br>uggcagugugguuagcugguug<br>uggcagugugguuagcugguugu<br>aacggcuaucaccacugccgucc<br>uggcagugugguuagcugguug<br>uggcagugugguuagcugguug	-	gcggauaagucaccggcuggcagugugguuagcugguugucagaugugagacaacggcuaucaccacugccguccugguggguucguccauccg
asu-mir-124	476	476	0	0	-	blast	uaaggcacgcggugaaugcca<br>uaaggcacgcggugaaugcca<br>uaaggcacgcggugaaugcc<br>uaaggcacgcggugaaugccaa<br>uaaggcacgcggugaaugcca<br>uaaggcacgcggugaaugcca<br>uaaggcacgcggugaaugccaa<br>uaaggcacgcggugaaugcca<br>uaaggcacgcggugaaugcca<br>cgccuucaccggugacuuuggu<br>uaaggcacgcggugaaugcca<br>uaaggcacgcggugaaugcca<br>uaaggcacgcggugaaugccaau<br>uaaggcacgcggugaaugc<br>uaaggcacgcggugaaugcca<br>uaaggcacgcggugaaugcc<br>uaaggcacgcggugaaugcca<br>uaaggcacgcggugaaugcca	-	uuugcgacacgccuucaccggugacuuuggugugugaagcagccuacuaaggcacgcggugaaugccaaucgcucuug
hpo-mir-9	504	504	0	0	-	blast	ucuuugguuaucuagcuguauga<br>cuuugguuaucuagcuguauga<br>ucuuugguuaucuagcuguaug<br>ucuuugguuaucuagcuguauga<br>ucuuugguuaucuagcuguauga<br>ucuuugguuaucuagcuguauga<br>ucuuugguuaucuagcuguauga<br>auaaggcuggacgacuaaaggaa<br>ucuuugguuaucuagcuguauga	-	gucauugaucucuuugguuaucuagcuguaugauuuuucguacucauaaggcuggacgacuaaaggaaucaauucguc
emu-mir-124a	592	592	0	0	-	blast	uaaggcacgcggugaaugcca<br>uaaggcacgcggugaaugcc<br>uaaggcacgcggugaaugcca<br>uaaggcacgcggugaaugcca<br>uaaggcacgcggugaaugccaa<br>uaaggcacgcggugaaugcca<br>uaaggcacgcggugaaugcca<br>uaaggcacgcggugaaugccaa<br>uaaggcacgcggugaaugcca<br>uaaggcacgcggugaaugcca<br>uaaggcacgcggugaaugcca<br>uaaggcacgcggugaaugcca<br>gcguucaaccucggagccuuuguc<br>uaaggcacgcggugaaugc<br>uaaggcacgcggugaaugcca<br>uaaggcacgcggugaaugcc<br>gcguucaaccucggagccuuuguc<br>uaaggcacgcggugaaugcca	-	ugucaagcacgugaaauggcguucaaccucggagccuuugucagcgauugaauuaaggcacgcggugaaugccaaaugcgaugcucc
hpo-mir-100	140	140	0	0	-	blast	aacccguagauccgaacuuguguu<br>aacccguagauccgaacuugug<br>acaggcucggcuacugguaga<br>aacccguagauccgaacuuguguu<br>aacccguagauccgaacuugugu<br>aacccguagauccgaacuugug	-	aacccguagauccgaacuuguguucaucauugugcuacacaggcucggcuacugguaga
asu-mir-9	504	504	0	0	-	blast	ucuuugguuaucuagcuguauga<br>ucuuugguuaucuagcuguauga<br>ucuuugguuaucuagcuguauga<br>ucuuugguuaucuagcuguaug<br>cuuugguuaucuagcuguauga<br>ucuuugguuaucuagcuguauga<br>auaaagcuggacgaccgaaguaa<br>ucuuugguuaucuagcuguauga<br>ucuuugguuaucuagcuguauga	-	gcaauuucuuugguuaucuagcuguaugaguaccgcguuuagucgucauaaagcuggacgaccgaaguaauggccgcu
mle-mir-34	28	28	0	0	-	blast	uggcagugugguuagcugguugu<br>uggcagugugguuagcugguug<br>uggcagugugguuagcugguugu<br>uggcagugugguuagcugguugu<br>uggcagugugguuagcugguugu<br>uggcagugugguuagcugguug<br>uggcagugugguuagcugguug<br>aaccacugauuacacuuccuug	-	uggcagugugguuagcugguuguguauccuuuaccauuacaaccacugauuacacuuccuug
emu-mir-9	2	2	0	0	-	blast	caaggcuagauuuccaaacaaa<br>ucuuugguuaucuagcugugug<br>caaggcuagauuuccaaacaaa<br>ucuuugguuaucuagcugugug	-	uaguugccauucuuugguuaucuagcugugugagguuuagcuucagucagcucuuauuuucacaaggcuagauuuccaaacaaacgcaucuaa
mco-mir-124a	378	378	0	0	-	blast	uaaggcacgcggugaaugcca<br>uaaggcacgcggugaaugcca<br>uaaggcacgcggugaaugcca<br>uaaggcacgcggugaaugcca<br>gcguuuauccgcgugacuuuaau<br>uaaggcacgcggugaaugcca<br>uaaggcacgcggugaaugcca<br>uaaggcacgcggugaaugcc<br>uaaggcacgcggugaaugcc<br>uaaggcacgcggugaaugcca<br>uaaggcacgcggugaaugcca<br>uaaggcacgcggugaaugcca<br>uaaggcacgcggugaaugcca<br>uaaggcacgcggugaaugcca<br>uaaggcacgcggugaaugc	-	gcguuuauccgcgugacuuuaauucgaaccggaaauaaggcacgcggugaaugcca
mco-mir-124b	1	0	0	1	-	blast	guauucuacgcggaugucuuggug	-	guauucuacgcggaugucuugguguggagcagacauuaaggcacgcggugaauac
ppc-mir-63a	13	0	0	13	-	blast	uaugacacugaagcgagauggu	-	ucucuccccucuaaucccccuuagccguccccuucucggcuuugguugucauugucuacuugaagagaacuaugacacugaagcgagaugguacggcgaggggaggaggaagagggaggg
asu-mir-100a-1	140	140	0	0	-	blast	aacccguagauccgaacuugug<br>aacccguagauccgaacuugugu<br>aacccguagauccgaacuuguguu<br>aacccguagauccgaacuugug<br>aacccguagauccgaacuuguguu<br>cgcaauucgccucucgggugca	-	ggcgguaaacccguagauccgaacuuguguuguguauuugguaacgcaauucgccucucgggugcaucgcugauuag
sme-mir-124c-1	378	378	0	0	-	blast	uaaggcacgcggugaaugcca<br>uaaggcacgcggugaaugcca<br>uaaggcacgcggugaaugcca<br>uaaggcacgcggugaaugc<br>uaaggcacgcggugaaugcca<br>uaaggcacgcggugaaugcc<br>gcgcucaccucgugaccuuugu<br>uaaggcacgcggugaaugcca<br>uaaggcacgcggugaaugcca<br>uaaggcacgcggugaaugcca<br>uaaggcacgcggugaaugcc<br>uaaggcacgcggugaaugcca<br>uaaggcacgcggugaaugcca<br>uaaggcacgcggugaaugcca<br>uaaggcacgcggugaaugcca	-	ugauccuauucuuuacuagcgcucaccucgugaccuuugucaguguuggacuaaggcacgcggugaaugccagaugaaaggauga
bma-let-7	1390	1390	0	0	-	blast	ugagguaguagguuguauaguu<br>cuguccagccugcuagcuugcc<br>ugagguaguagguuguauaguu<br>ugagguaguagguuguauaguu<br>ugagguaguagguuguauaguu<br>ugagguaguagguuguauaguu<br>ugagguaguagguuguauaguu<br>ugagguaguagguuguauagu<br>ugagguaguagguuguauaguu<br>ugagguaguagguuguauaguu<br>ugagguaguagguuguauaguu	-	augcuaacgacccugaaugauagcuugcucgaugagguaguagguuguauaguuaugaguuguaccauaaucggagaaacuguccagccugcuagcuugccgugcgggucuaucauccguccccc
sja-mir-124	6	6	0	0	-	blast	uaaggcacgcggugaauguca<br>uuaaggcacgcggugaauguca<br>ccauuuuccgcgauugccuugauuu<br>uaaggcacgcggugaauguca	-	uauauguaugccauuuuccgcgauugccuugauuuguuaaaagaaaaugauucacaacaaaauauuaaggcacgcggugaaugucauccacggugc
cel-let-7	1595	1595	0	0	-	blast	ugagguaguagguuguauaguu<br>ugagguaguagguuguauaguu<br>ugagguaguagguuguauaguu<br>ugagguaguagguuguauaguu<br>ugagguaguagguuguauaguu<br>cuaugcaauuuucuaccuuacc<br>ugagguaguagguuguauaguu<br>ugagguaguagguuguauagu<br>ugagguaguagguuguauaguu<br>ugagguaguagguuguauaguuu<br>ugagguaguagguuguauaguu<br>ugagguaguagguuguauaguu	-	uacacuguggauccggugagguaguagguuguauaguuuggaauauuaccaccggugaacuaugcaauuuucuaccuuaccggagacagaacucuucga
mle-mir-124	518	518	0	0	-	blast	uaaggcacgcggugaaugcca<br>uaaggcacgcggugaaugcca<br>uaaggcacgcggugaaugc<br>uaaggcacgcggugaaugcca<br>uaaggcacgcggugaaugcca<br>uaaggcacgcggugaaugcc<br>uaaggcacgcggugaaugcca<br>uaaggcacgcggugaaugcca<br>uaaggcacgcggugaaugcca<br>uaaggcacgcggugaaugcc<br>uaaggcacgcggugaaugcca<br>guguucacuguguaagccuuggu<br>uaaggcacgcggugaaugcca<br>uaaggcacgcggugaaugcca<br>uaaggcacgcggugaaugcca	-	guguucacuguguaagccuuggugugugugcacgauuaaggcacgcggugaaugcca
cel-mir-124	378	378	0	0	-	blast	uaaggcacgcggugaaugcc<br>uaaggcacgcggugaaugcca<br>gcaugcacccuagugacuuuagu<br>uaaggcacgcggugaaugcca<br>uaaggcacgcggugaaugcca<br>uaaggcacgcggugaaugcca<br>uaaggcacgcggugaaugcca<br>uaaggcacgcggugaaugc<br>uaaggcacgcggugaaugcca<br>uaaggcacgcggugaaugcca<br>uaaggcacgcggugaaugcca<br>uaaggcacgcggugaaugcca<br>uaaggcacgcggugaaugcca<br>uaaggcacgcggugaaugcc<br>uaaggcacgcggugaaugcca	-	gucccacuugucaucuggcaugcacccuagugacuuuaguggacaucuaagucuuccaacuaaggcacgcggugaaugccacguggccaugauggg
hpo-let-7	1320	1320	0	0	-	blast	ugagguaguagguuguauaguu<br>ugagguaguagguuguauaguu<br>ugagguaguagguuguauaguu<br>ugagguaguagguuguauaguu<br>cuauuacaacuugcuagcuugc<br>ugagguaguagguuguauaguu<br>ugagguaguagguuguauaguu<br>ugagguaguagguuguauaguu<br>ugagguaguagguuguauagu<br>ugagguaguagguuguauaguu<br>ugagguaguagguuguauaguu	-	ugagguaguagguuguauaguucagcguggcaucaucaggugaaacuauuacaacuugcuagcuugc
